# Instruments to assess quality of life in people with mental health problems: a systematic review and dimension analysis of generic, domain- and disease-specific instruments

**DOI:** 10.1186/s12955-021-01883-w

**Published:** 2021-11-02

**Authors:** F. C. W. van Krugten, K. Feskens, J. J. V. Busschbach, L. Hakkaart-van Roijen, W. B. F. Brouwer

**Affiliations:** 1 Erasmus School of Health Policy and Management, Erasmus University Rotterdam, PO Box 1738, 3000 DR Rotterdam, The Netherlands; 2 Department of Psychiatry, Erasmus Medical Center, Rotterdam, The Netherlands

**Keywords:** Quality of life, Patient-reported outcome measures, Mental health, Systematic review, Cost-effectiveness, QALY

## Abstract

**Objectives:**

The importance of economic evaluations of mental healthcare interventions is increasingly recognized. Despite the multitude of available quality of life instruments, concerns have been raised regarding the content validity of these instruments, and hence suitability for use in mental health. The aim of this paper, therefore, was to assess the content validity and the suitability of existing quality of life instruments for use in economic evaluations in mental health problems.

**Methods:**

In order to identify available quality of life instruments used in people with mental health problems, a systematic review was performed using the Embase, Medline and PsycINFO databases (time period January 2012 to January 2018). Two reviewers independently assessed study eligibility and executed data extraction. The evaluation framework of Connell and colleagues was used to assess whether the identified quality of life instruments cover the dimensions valued highly by people with mental health problems. Two reviewers independently mapped the content of each identified instrument onto the evaluation framework and indicated the extent to which the instrument covered each of the dimensions of the evaluation framework.

**Results:**

Searches of databases yielded a total of 5727 references. Following duplicate removal and double-independent screening, 949 studies were included in the qualitative synthesis. A total of 44 unique quality of life instruments were identified, of which 12 were adapted versions of original instruments. The best coverage of the dimensions of the evaluation framework of Connell and colleagues was by the WHOQOL-100, S-QoL, SQLS, EDQoL, QLI and the IMHQOL, but none fully covered all dimensions of the evaluation framework.

**Conclusions:**

The results of this study highlight the multitude of available quality of life instruments used in people with mental health problems and indicate that none of the available quality of life instruments fully cover the dimensions previously found to be important in people with mental health problems. Future research should explore the possibilities of refining or expanding existing instruments as well as the development and testing of new quality of life instruments to ensure that all relevant quality of life dimensions for people with mental health problems are covered in evaluations.

**Supplementary Information:**

The online version contains supplementary material available at 10.1186/s12955-021-01883-w.

## Introduction

In the context of scarce resources and rising demands for healthcare, the importance of economic evaluations of healthcare interventions to aid decision makers in allocating healthcare resources is increasingly recognized [1, 2]. Such compare the costs and benefits of healthcare interventions, relative to a relevant comparator, in order to assess their value for money. While costs are typically expressed in monetary terms in such evaluations, benefits are usually expressed in quality-adjusted life-years (QALYs). QALYs comprise changes in both length and quality of life, with the latter typically being measured by generic health-related quality of life instruments, which facilitates comparisons across conditions and interventions [3, 4]. Given the importance of quality of life measurement and valuation in economic evaluations, it is vital to ensure that the instruments used are comprehensive and psychometrically sound.

In the mental health field, the need to assess the relative value for money of different interventions, to inform healthcare resource allocation decisions at different levels, has also been recognised. However, in that context, there is an ongoing debate about how and with which instruments the benefits of mental healthcare interventions could be adequately measured and valued [5, 6]. This topic is particularly relevant for mental health interventions, since alleviating symptoms and improving quality of life are common goals of mental health interventions, rather than prolonging length of life. The adequacy of often used generic health-related quality of life instruments, such as the EuroQol five-dimensional (EQ-5D) questionnaire and the 36-item Short-Form Health Survey (SF-36), has been questioned in the context of (parts of) mental healthcare [5, 6]. More specifically, some have suggested that these instruments, in certain situations, lack the sensitivity to sufficiently reflect the impact of mental health problems on quality of life [7], which is obviously problematic. The EQ-5D, for example, appears to perform well in mild to moderate mental health conditions [8, 9], but showed weak correlations with severe mental health problems such as schizophrenia [6]. Some argue that this may be due to the fact that these commonly used quality of life instruments have been developed top-down by clinicians or other experts and primarily for people with a physical illness, thereby limiting the coverage of dimensions perceived important to the quality of life of people with mental health problems [10]. Hence, the debate in this area relates both to the sensitivity of existing health-related quality of life instruments, but also to the scope of relevant outcomes (i.e. potentially broadening the evaluative space). The latter is analogous to discussions related to outcome measurements in economic evaluations in elderly care [11]. Another explanation could be that generic instruments by definition focus on the most important quality of life dimensions across diseases, and hence may focus less on particular dimensions relevant in specific diseases. This highlights the tension between the use of generic instruments and more domain or disease specific instruments, which is characterized by a trade-off between comparability between diseases and sensitivity within a disease.

In order to adequately measure and value the benefits of mental healthcare interventions, the use of a multidimensional, preference-based instrument that comprehensively captures the benefits of mental healthcare interventions is required. Based on previous work by Connell and colleagues [12, 13] that identified seven dimensions known to be important to the quality of life of people with mental health problems, the aim of this paper was to assess the content validity of quality of life instruments used in the mental health field. In addition, it was evaluated whether the available instruments are suitable or, on the basis of the content validity, can be made suitable for use in economic evaluations. The results of this study may then enhance the selection of the most suitable instruments in terms of their coverage of dimensions and benefit the development of adequate outcome instruments to measure and value the benefits of mental healthcare interventions.

## Methods

### Data sources and search strategy

In order to identify available quality of life instruments used in people with mental health problems, a systematic literature search was conducted on the Embase, Medline and PsycINFO databases in accordance with the Preferred Reporting Items for Systematic Reviews and Meta-Analyses (PRISMA) statement [14]. The search was conducted on January 3, 2018 and was restricted to studies published between January 1, 2012, to January 3, 2018. The search strategy combined terms related to quality of life (e.g. 'quality of life', 'quality of life assessment') and terms related to a broad range of clinical and subclinical mental health problems. See Additional file 1 for the search strategies. We did not register a protocol for the review.

### Eligibility criteria

Studies were selected for inclusion if they met all of the following criteria:

1. The study population consisted of patients 18 years or older with a clinical or subclinical primary mental health problem;
2. Quality of life was an explicit outcome measure;
3. Quality of life was measured as a multidimensional construct through a generic, domain (i.e. mental health), or disease-specific quality of life instrument with established psychometric properties;
4. The study was a randomized controlled trial, case–control study, cross-sectional study, or cohort study;
5. Published in English and full text available.

Exclusion was based on not meeting all eligibility criteria. Hence, studies that did not meet one or more of the above-listed eligibility criteria were excluded from the review. We emphasise that our review was not restricted to preference-based instruments, but also included ‘non-preference-based’ instruments. For preference-based or preference-accompanied instruments a value set is available of ‘utility scores’ that reflect the relative importance of or preference for the states described with such instruments. Such ‘utility scores’ are typically obtained in a representative sample of the general population and, if derived appropriately, enable the generation of health state utility values for the states described with the instrument. Health state utility values are used to calculate quality-adjusted life-years (QALYs) in economic evaluations of (mental) healthcare interventions. The outcomes of such evaluations can be used in funding and allocation decisions in healthcare. The most frequently used preference-based instrument is the EQ-5D. Other well-known preference-based instruments are the SF-36 and the Health Utility Index (HUI).

The inclusion of both preference-based instruments and non-preference-based instruments in this review was motivated by the fact that this allows the identification of the outcome instruments currently used in the mental health field, and whether those instruments cover the seven relevant dimensions. This was deemed important since comprehensive non-preference-based instruments could potentially be made suitable for use in economic evaluations by deriving utility scores for the states described with such instruments. Indeed, for instruments to be used in economic evaluations the availability of utility scores that indicate the value of health states are a main requirement, so that the value of changes therein can also be assessed (which can subsequently be compared to the costs required to produce these changes in order to judge value for money).

### Study selection and data abstraction

Search results were compiled and deduplicated using RefWorks (http://www.refworks.com), a web-based, bibliographic citation manager. Prior to the eligibility assessment of all identified references, two reviewers (FK, KF) independently screened a random sample of 166 titles and abstracts, and reached strong agreement (Cohen’s κ = 0.83). Blinded to journal titles and authors, the two reviewers then independently screened titles and abstracts of all identified references for potential eligibility using a standardized Excel workbook. For all references that were potentially eligible, a full-text version was retrieved and independently assessed by the two reviewers. Disagreements were resolved by discussion or through third-party adjudication (LH). Data abstraction was performed in duplicate and independently using a standardized, Excel-based data abstraction form. The following data were extracted from the included studies: (1) general study characteristics (year of publication, continent of study origin); (2) sample size; (3) (sub)clinical diagnosis of study population; (4) quality of life instrument(s) used. Following the data abstraction of included studies, the development papers and original instruments of identified quality of life instruments were retrieved online or requested from the author. A detailed risk of bias assessment of the included studies was not performed as the primary objective of the review was to compile a list of quality of life instruments used in people with mental health problems.

### Evaluation of identified instruments

The following aspects of each of the identified instruments were evaluated: (1) type (generic, domain, or disease-/subgroup-specific); (2) number of items; (3) number of dimensions; (4) region of development; (5) availability of preferences weights (yes/no). The availability of preferences weights was evaluated in order to assess the instruments' suitability for use in cost-effectiveness studies. Such preference-based weights may also help to get an idea about the relative importance of (changes in) different domains and levels. Adapted versions of original instruments were analysed separately as their number of items as well as their number and type of dimensions covered could differ from the original instrument.

An evaluation framework of dimensions was established in order to assess whether the identified quality of life instruments cover the dimensions valued highly by people with mental health problems. The evaluation framework was established based on previous work of Connell and colleagues [12, 13] who identified seven dimensions known to be important elements of the quality of life of people with mental health problems: well-being and ill-being; relationships and belonging; activity; self-perception; autonomy; hope and hopelessness; physical health. The work by Connell and colleagues [12, 13] was selected as the basis for our evaluation framework, given that it specifically aimed to identify the dimensions of quality of life important to people with mental health problems by using a rigorous mixed-methods approach, i.e. combining a systematic review of qualitative research [12] with complementary interviews [13].

Two reviewers (FK, KF) independently mapped the content of each quality of life instrument onto the evaluation framework and indicated the extent to which the instrument covered each of the dimensions of the evaluation framework: fully covered, partially covered, not covered. A dimension of the evaluation framework was scored as 'fully covered' when the content of the identified quality of life instrument covered more than 75% of the underlying themes of a dimension of the evaluation framework of Connell and colleagues [12, 13] and can be also be found in Additional file 2. Likewise, a dimension was scored as 'partially covered' when the dimensions covered less than 75% of the underlying themes of the dimensions of the evaluation framework. A dimension was scored as ‘not covered' when the dimensions covered none of the underlying themes of the dimensions of the evaluation framework. Disagreements were resolved by discussion or through third-party adjudication (LH).

## Results

### Study selection and study characteristics

**Fig. 1:**
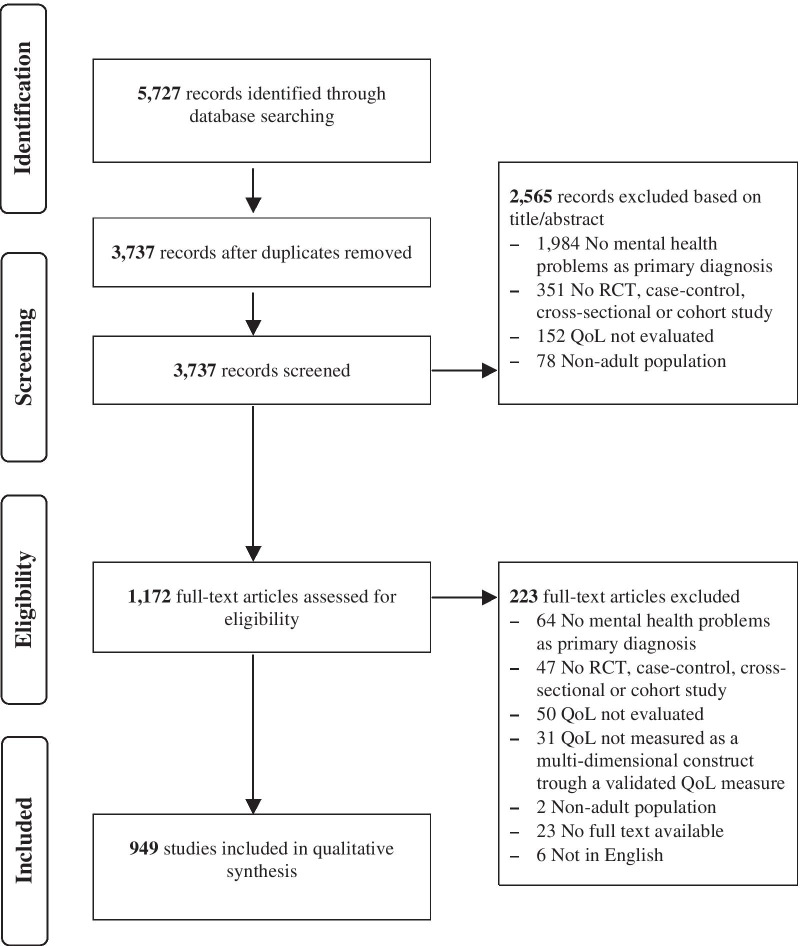
Flow chart of study selection process. RCT, Randomized controlled trial; QoL, quality of life

The primary search of databases yielded 5727 references. After duplicate removal and subsequent title and abstract screening, 1172 papers were obtained for full-text review. Following full-text review, 949 studies met the inclusion criteria and were included in the qualitative synthesis. See Fig. 1 for the flow chart of the study selection process and Additional file 3 for the reference list of the included studies.

An overview of the general characteristics of the included studies is shown in Table 1. Most of the studies were conducted in Europe (35.6%), followed North America (24.9%), and Asia (23.0%). The most frequently studied diagnosis was schizophrenia spectrum and other psychotic disorders, which was the primary diagnosis of the patient population in 31.3% of the included studies.

**Table 1:** General characteristics of the included studies (N = 949)

	N	%
*Year of publication*		
2012	140	14.8
2013	158	16.6
2014	132	13.9
2015	188	19.8
2016	184	19.4
2017	147	15.5
*Study region*		
Africa	13	1.4
Asia	218	23.0
Oceania	39	4.1
Europe	338	35.6
Middle east	49	5.2
North America	236	24.9
South America	53	5.6
Covering various regions	3	0.3
*Mental health disorder(s) of the study population*^a,b^		
Anxiety disorders	41	4.3
Bipolar or related disorders	53	5.6
Depressive disorders	128	13.5
Feeding and eating disorders	42	4.4
Gender dysphoria	2	0.2
Neurodevelopmental disorders	23	2.4
Obsessive–compulsive and related disorders	25	2.6
Personality disorders	4	0.4
Schizophrenia spectrum and other psychotic disorders	297	31.3
Sleep–wake disorders	5	0.5
Somatic symptom and related disorders	7	0.7
Substance-related and addictive disorders	101	10.6
Trauma and stressor-related disorders	55	5.8
Various disorders	166	17.5

^a^Disorders were grouped according to the diagnostic and statistical manual (DSM)-5 categories

^b^Disorder in this case refers to both clinical and subclinical mental health disorders

### Characteristics of identified instruments

A total of 44 quality of life instruments were identified in the primary search, of which 12 were adapted versions of original instruments. Of all instruments, the World Health Organization's Quality of Life Instrument-Short Version (WHOQOL-BREF) [15] was used most frequently (n = 240, 23.9%), followed by the 36-item Short-Form Health Survey (SF-36) [16] (n = 181, 18.0%). Of the 44 identified instruments, 16 were generic instruments, 9 were domain-specific (i.e. mental health specific) instruments and 19 were disease- or subgroup-specific instruments. Generic instruments were the most commonly used (65.0%), followed by domain-specific instruments (20.3%), and disease- and subgroup-specific instruments (14.7%). Of the disease- and subgroup-specific instruments, six were developed for schizophrenia, five for eating disorders, two for veterans, while one was developed for attention-deficit/hyperactivity disorder (ADHD), bipolar depression, Gilles de la Tourette syndrome, forensic inpatients, older people, and patients under neuroleptic treatments. On average, the identified instruments included 35 items (median = 23, range = 5–143), and covered and average of 7 dimensions (median = 7, range = 2–17). Five instruments allowed for utility score calculations: the Short Form-6 Dimensions (SF-6D) [17], the EuroQol five-dimensional questionnaire (EQ-5D) [18], the Assessment of Quality of life-4 Dimensions (AQoL-4D) [19], the Assessment of Quality of life-8 Dimensions (AQoL-8D) [20], and the 15 Dimensional (15D) [21]. See Table 2 for the general characteristics of the identified instruments and Tables 4, 5 and 6 for the complete list of identified quality of life instruments, their frequency of use, and number of items and dimensions.

**Table 2:** General characteristics of identified instruments

	All instruments (N = 44)	Generic instruments (N = 16)	Domain-specific instruments (N = 9)	Disease- and subgroup-specific instruments (N = 19)
Frequency of use (N, %)	1004 (100.0)	653 (65.0)	204 (20.3)	147 (14.7)
*Number of items*				
Mean (SD)	37.2 (35.5)	23.3 (25.8)	66.2 (50.7)	35.1 (26.8)
Median	23	14	78	27
Range	4–143	4–100	15–143	12–131
*Number of dimensions*				
Mean (SD)	7.6 (3.8)	6.1 (3.0)	10.8 (3.7)	7.4 (3.7)
Median	8	6	9	8
Range	2–17	3–15	8–17	2–15
Number of adapted versions (N, %)	12 (27.3)	9 (56.3)	1 (11.1)	4 (21.1)
Utility score available, Yes (N, %)	5 (11.4)	5 (31.3)	0 (0.0)	0 (0.0)

SD, standard deviation

### Instruments' coverage of dimensions of the evaluation framework

The identified instruments differed in the extent to which they covered the dimensions of the evaluation framework (Tables 3, 4, 5, 6). The "Relationships and belonging" dimension was the most frequently covered (93%), followed by the "Activity" (89%), and "Physical health" (86%) dimensions. The least covered dimensions were the "Self-perception" and "Hope and hopelessness" dimensions, which were included in only 57% and 32% of all instruments, respectively. Compared to the generic instruments and disease- and subgroup-specific instruments, the quality of life instruments specially designed for use in the mental health field covered the "Relationships and belonging", "Activity", and "Autonomy" dimensions most frequently. Of all identified instruments, the World Health Organization's Quality of Life Instrument (WHOQOL-100) [22], the Schizophrenia Quality of Life Questionnaire 41 (S-QoL 41) [23], the Schizophrenia Quality of Life Scale (SQLS) [24], the Eating Disorder Quality of Life (EDQoL) [25], the Quality of Life Index (QLI) [26], and the Internet Mental Health Quality of Life scale (IMHQOL) [27] covered the dimensions of the evaluation framework best. None of the identified instruments fully covered all dimensions of the evaluation framework.

**Table 3:** Frequency (N (%)) with which the identified quality of life instruments (fully or partially) cover the dimensions of the evaluation framework

Quality of life dimension	All instruments (N = 44)	Generic instruments (N = 16)	Domain-specific instruments (N = 9)	Disease- and subgroup-specific instruments (N = 19)
Well-being and ill-being	36 (82)	13 (81)	6 (67)	17 (89)
Relationships and belonging	41 (93)	13 (81)	9 (100)	19 (100)
Activity	39 (89)	13 (81)	9 (100)	17 (89)
Self-perception	25 (57)	8 (50)	5 (56)	12 (63)
Autonomy	28 (64)	7 (44)	9 (100)	12 (63)
Hope and hopelessness	14 (32)	3 (19)	2 (22)	9 (47)
Physical health	38 (86)	16 (100)	9 (100)	13 (68)

**Table 4:**
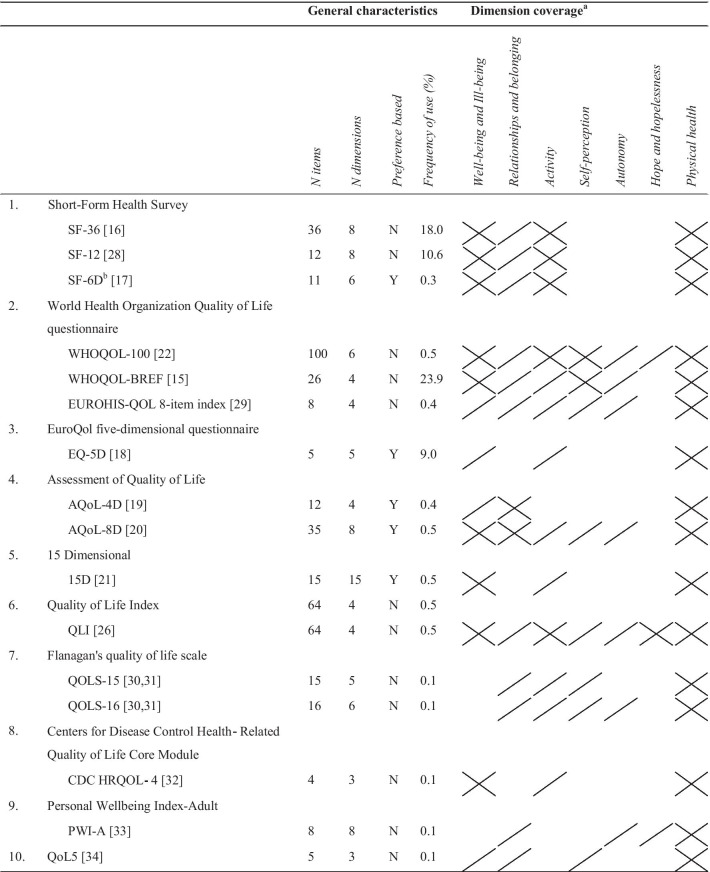
General characteristics and dimension coverage of identified generic instruments

Y, Yes; N, No

^a^  indicates the dimension is fully covered;  indicates the dimension is partially covered

^b^The SF-6D was the reported instrument in 3 studies (0.3%); these studies did not report the actual administered instrument (i.e. SF-36 or SF-12)

**Table 5:**
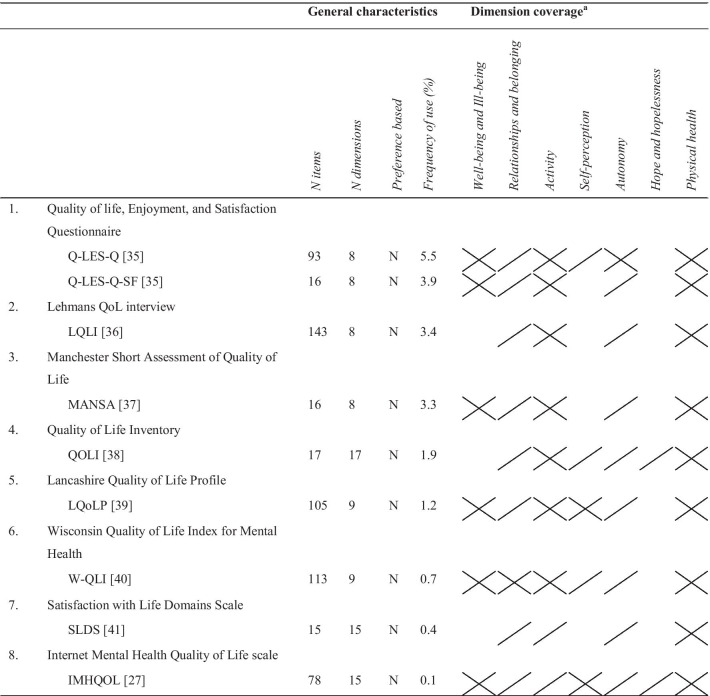
General characteristics and dimension coverage of identified domain-specific instruments

Y, Yes; N, No

^a^  indicates the dimension is fully covered;  indicates the dimension is partially covered

^b^The mapping of the content onto the evaluation framework was based on the description of the items and dimensions in the development paper, since the instrument itself could not be retrieved (online or from the author)

**Table 6:**
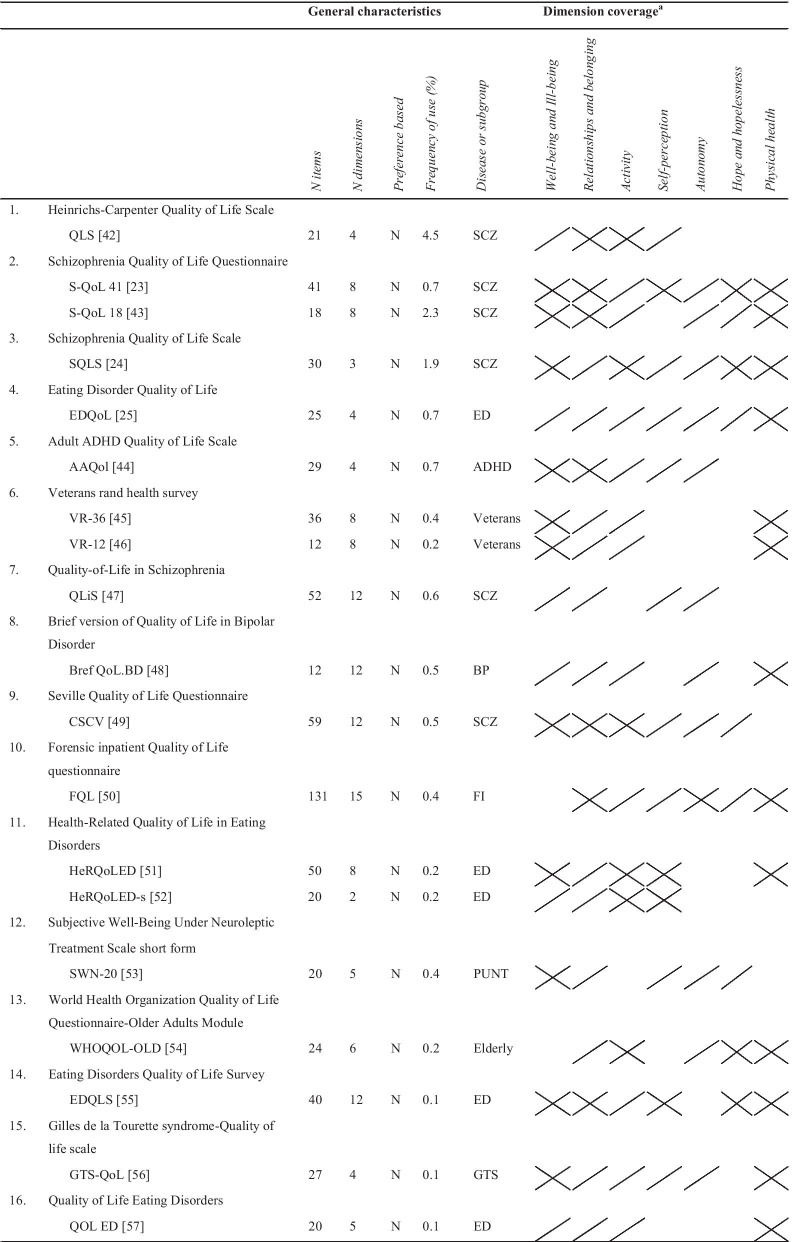
General characteristics and dimension coverage of identified disease- and subgroup-specific instruments

ADHD, Attention-Deficit/Hyperactivity Disorder; BP, Bipolar Depression; ED, Eating Disorders; FI, Forensic Inpatients; GTS, Gilles de la Tourette; N, No; PUNT, Patients Under Neuroleptic Treatment; SCZ, Schizophrenia; Y, Yes

^a^  indicates the dimension is fully covered;  indicates the dimension is partially covered

^b^The mapping of the content onto the evaluation framework was based on the description of the items and dimensions in the development paper, since the instrument itself could not be retrieved (online or from the author)

## Discussion

The aim of this systematic review was to assess the content validity and suitability for use in economic evaluations of quality of life instruments used in people with mental health problems. A total of 44 unique instruments were identified, of which 12 were adapted versions of original instruments. The evaluation framework of Connell and colleagues was used to assess whether the identified quality of life instruments cover the dimensions valued highly by people with mental health problems. The best coverage of the dimensions of the evaluation framework was by the WHOQOL-100, S-QoL 41, SQLS, EDQoL, QLI and IMHQOL, but none fully covered the dimensions of the evaluation framework. The instruments with the best coverage of the dimensions of the evaluation framework lack a preference-based scoring algorithm, at present. In line with the study of Touré and colleagues [58], it was found that all identified preference-based instruments, which were all generic, cover the dimension “Physical health”, but generally lack coverage of mental health related (sub)dimensions. Of the five instruments that were found to have a preference-based scoring algorithm, the AQoL-8D had the most overlap with the framework of Connell and colleagues [12, 13].

The results of this study highlight the multitude of available quality of life instruments and support previous research questioning the ability of commonly used instruments to adequately measure and value the benefits of mental healthcare interventions [10]. The findings of this review suggest that this inability might be related to the content validity of the available quality of life instruments, since none of the identified preference-based instruments was found to fully cover the dimensions known valued highly by people with mental health problems. Noteworthy was the lack of coverage of the "Hope and hopelessness" and "Self-perception" dimensions, which were covered in only 14% and 34% of the identified instruments, respectively. Note that the low coverage of the “Hope and Hopelessness” dimension may be explained by the fact that this dimension may be, to a certain degree, transversal to depression and distress, which were underlying themes of the “Well-being” dimension. It is important to recognize differences in the coverage of dimensions in selecting the quality of life instruments of choice for evaluating the effectiveness of interventions, as they implicitly define the maximand of interventions. Another noteworthy finding was that the majority of identified instruments are non-preference-based and are, therefore, not directly useful for inclusion in cost-utility studies. In order to make available instruments suitable for use in cost-utility studies, health state utility values should be generated by use of utility-elicitation procedures or, as a second-best option, predicted by statistical association [59]. However, given that none of the identified instruments fully cover the dimensions valued highly by people with mental health problems, it seems advisable to first refine existing instruments or develop new quality of life instruments that cover all of the relevant dimensions. In the refinement or development of such instruments, next to their content validity, other elements of validity and reliability require much attention. Even more so, as, particularly in the mental health field, self-completion instruments may be less reliable in certain disease areas, as affected by the illness itself, and may be prone to bias due to effects of social desirability and stigma. In addition, in order to sufficiently reflect the impact of mental health problems on quality of life, but simultaneously prevent a loss of comparability of utility values across mental health diagnoses, such new instruments should preferably be domain-specific (i.e. mental health) in nature. It needs noting that such a strategy does raise numerous questions about the desired scope of such instruments and the subsequent comparability of outcomes across sectors. In other words, optimisation per domain may compromise the optimisation over domains. These issues are beyond the scope of the current review but require attention in future research.

This systematic review is strengthened by its use of a comprehensive search strategy, the bias protection measures taken (e.g. the independent and duplicate screening and reviewing of identified studies), the executed dimension analysis of identified instruments based on a scientifically founded evaluation framework, and the inclusion of studies focusing on populations with clinical and subclinical primary mental health problems.

Despite the strengths of this review, some limitations should be noted. First, the review was restricted to peer-reviewed studies published in the Embase, Medline and PsycINFO databases. Expanding the search strategy by, for instance, including grey literature, using snowballing or including other databases such as the Cochrane Central Register of Controlled Trials, might have produced (even) more results. Hence, some relevant studies may have been missed in the current review. Second, most of the included studies were conducted in Europe, North America, and Asia. Future research could explore the reasons for the relatively low frequency of use of quality of life instruments in mental health research in other continents. Third, given our focus on published studies up to 2018, we may have missed recent developments in the field of quality of life assessment. One important quality of life instrument, specifically designed for use in the mental health field, that has become available since the completion of our review is the Recovering Quality of Life (ReQoL) measure [60]. The ReQoL measure is a preference-based [61] patient reported outcome measure that was explicitly designed to cover all seven dimensions of the evaluation framework used in the current study. The development of the ReQoL measure highlights the need and search for outcome instruments that adequately measure and value the benefits of mental healthcare interventions. Further work is required to assess how the ReQoL performs in various contexts, especially in the contexts in which existing quality of life measures lack the sensitivity to sufficiently reflect the impact of mental health problems on quality of life, and in relation to other outcome measures identified in this study. Fourth, given the focus on the identification of quality of life instruments used in people with mental health problems, we might have missed relatively new instruments that were available but not used in studies published in the reference period of our search. The Clinical Outcomes in Routine Evaluation (CORE)-6D [62] is an example of such a measure. Given the rapid developments in this field, it is advisable that studies like the present one are repeated in the future. Fifth, since the aim of the review was to identify available quality of life instruments used in people with mental health problems and assess whether these instruments cover the dimensions found to be important in people with mental health problems (content validity), the analysis does not take anything regarding the other psychometric performance of the identified instruments into account. Inclusion of quality of life instruments in studies on the (cost-)effectiveness of mental health interventions should be based on and motivated by evidence on all psychometric properties of the instruments, as for example assessed by the COnsensus-based Standards for the selection of health Measurement INstruments (COSMIN) [63]. Hence, even if instruments cover most of the dimensions of the evaluation framework, it does not imply that these instruments are recommended over others, nor does it imply that these instruments are the best available for use in people with mental health problems. In addition, failure to meet the criteria of the evaluation framework is not a disqualification of the instrument as such, but it raises questions about the suitability of the instruments when used in the context of mental health. The findings of this study could, however, enhance the selection of the most suitable instruments in terms of their coverage of dimensions and practical characteristics such as number of items and the availability of preference-based utility values. Sixth, the study population of one of the studies underlying the evaluation framework [13] only included mental healthcare service users, not a wider population of people with mental health problems. This may have influenced the dimensions of quality of life in the framework. However, in the absence of studies examining the important quality of life dimensions in a broader, mixed population with people with mental health problems, the study carried out by Connell and colleagues [13] was considered the best available to base the framework on. Seventh, the adoption of the framework by Connell and colleagues [12, 13] implicitly implies that life domains considered important by the relevant population should determine the evaluative scope of an economic evaluation. This matter can be debated and relates to normative questions of what should be maximized (health or more general well-being), whether outcome measures should be generic or may be domain-specific, and the appropriate source for domains and their relative valuations. These are crucial questions that fall outside the scope of the current study. Eighth, the mapping of the dimensions of the identified instruments onto the evaluation framework was inherently subjective. In order to minimise the subjective nature of the mapping procedure, the dimensions of each identified instrument were assessed and mapped onto the evaluation framework by two reviewers in a structured, independent manner using standardized criteria.

## Conclusions

The results of this study highlight the multitude of available quality of life instruments and lack of consensus regarding the choice of instruments used in people with mental health problems. In addition, the results could enhance the selection of the most suitable instruments in terms of their coverage of dimensions and practical characteristics. At the same time, the increasing importance of quality of life measurement in clinical and research settings emphasizes the need for more methodological studies on quality of life measurement in the mental health field. More specifically, future research could evaluate and compare the psychometric properties of promising instruments, and obtain preference-based utility values for these instruments to make them suitable for use in cost-effectiveness studies. In addition, since the results of this study suggest that none of the identified instruments cover all the dimensions found to be important in people with mental health problems, future research could explore the possibilities of refining existing instruments or the development of a new quality of life instrument that covers all of the dimensions valued highly by people with mental health problems. Future research on these issues remains crucial to capture the benefits of interventions targeted at people with mental health problems and facilitate the comparison of the clinical and cost-effectiveness of mental healthcare interventions, which in turn could improve the allocation of scarce resources in the mental health field.

## Supplementary Information

**Additional file 1**. Search strategies.

**Additional file 2**. Underlying themes. 

**Additional file 3**. References of included studies. 

## Data Availability

The dataset used and/or analysed during the current study are available from the corresponding author on reasonable request.

## Abbreviations

15D: 15 Dimensional
AAQol: Adult ADHD quality of life scale
ADHD: Attention-deficit/hyperactivity disorder
AQoL-4D: Assessment of quality of life-4 dimensions
AQoL-8D: Assessment of quality of life-8 dimensions
Bref QoL.BD: Brief version of quality of life in bipolar disorder
CDC HRQOL‐4: Centers for Disease Control Health‐Related Quality of Life Core Module
COSMIN: COnsensus-based Standards for the selection of health Measurement INstruments
CSCV: Seville Quality of Life Questionnaire
DSM: Diagnostic and statistical manual
EDQLS: Eating disorders quality of life survey
EDQoL: Eating disorder quality of life
EDQoL: Eating disorder quality of life
EQ-5D: EuroQol five-dimensional
EUROHIS-QOL: European health interview survey-quality of life
FQL: Forensic Inpatient Quality of Life Questionnaire
GTS-QoL: Gilles de la Tourette syndrome-quality of life scale
HeRQoLED: Health-related quality of life in eating disorders
IMHQOL: Internet mental health quality of life scale
LQoLP: Lancashire quality of life profile
MANSA: Manchaster short assessment of quality of life
PRISMA: Preferred reporting items for systematic reviews and meta-analyses
PWI-A: Personal wellbeing index-adult
Q-LES-Q-SF: Quality of Life, Enjoyment, and Satisfaction Questionnaire-Short Form
Q-LES-Q: Quality of Life, Enjoyment, and Satisfaction Questionnaire
QALYs: Quality-adjusted life-years
QLI: Quality of life index
QLiS: Quality-of-life in schizophrenia
QLS: Heinrichs-carpenter quality of life scale
QOL ED: Quality of life eating disorders
QoL: Quality of life
QOLI: Quality of life inventory
QOLS: Quality of life scale
RCT: RCT Randomized controlled trial
ReQoL: Recovering quality of life
S-QoL 41: Schizophrenia Quality of Life Questionnaire 41
S-QoL: Schizophrenia Quality of Life Questionnaire
SD: Standard deviation
SF-12: 12-Item short-form health survey
SF-36: 36-Item short-form health survey
SF-6D: Short form-6 dimensions
SLDS: Satisfaction with life domains scale
SQLS: Schizophrenia quality of life scale
SQLS: Schizophrenia quality of life scale
SWN-20: Subjective well-being under neuroleptic treatment scale short form
VR: Veterans rand health survey
W-QLI: Wisconsin quality of life index for mental health
WHOQOL-100: World Health Organization's Quality of Life Instrument
WHOQOL-BREF: World Health Organization's Quality of Life Instrument-Short Version
WHOQOL-OLD: World Health Organization Quality of Life Questionnaire-Older Adults Module
