# Supplementary material for: Instruments to assess quality of life in people with mental health problems: a systematic review and dimension analysis of generic, domain- and disease-specific instruments
Source: Health Qual Life Outcomes. 2021 Nov 2;19:249. doi: 10.1186/s12955-021-01883-w (PMC8561965; doi:10.1186/s12955-021-01883-w)
Supplement: Supplementary file 1 — Additional file 1. Search strategies. [file 12955_2021_1883_MOESM1_ESM.pdf]

Additional file 1 - Search strategies

Embase

('quality of life'/mj/exp OR 'quality of life assessment'/mj/exp OR ((quality NEAR/3 life) OR qol):ti) AND ('mental health'/mj/exp OR 'psychiatry'/mj/exp OR 'Diagnostic and Statistical Manual of Mental Disorders'/mj/exp OR 'mental disease'/exp/mj OR 'sexual deviation'/mj OR (((mental* OR psychic*) NEAR/3 (health* OR disorder* OR ill*)) OR (mood NEAR/3 disorder*) OR (obsessi* NEAR/3 compuls*) OR dsm OR psychiatr* OR schizo* OR bipolar OR unipolar OR ((posttrauma* OR trauma*) NEAR/3 stress) OR ptsd OR ((dissociati* OR somatoform* OR eating OR personalit* OR psychosexual* OR behav* OR psychosocial*) NEAR/3 disorder*) OR (multiple NEAR/3 personalit*) OR anorex* OR bulemi* OR addict* OR ((drug* OR alcohol* OR substance*) NEAR/3 (depend* OR misuse OR abuse)) OR borderlin* OR depressi* OR anxi* OR panic OR (Diagnos* NEAR/3 Statistic* NEAR/3 Manual*) OR 'sexual* deviat*' OR paraphil* OR psychosis OR psychoses OR psychotic OR phobia*):ti) NOT ([Conference Abstract]/lim OR [Letter]/lim OR [Note]/lim OR [Editorial]/lim) AND [english]/lim NOT (cancer* OR malign* OR neoplas* OR hiv OR aids OR diabet* OR dement* OR cardiac* OR myocard* OR surg* OR postsurg* OR postoperat* OR stroke* OR cva):ti NOT ((j uvenile/exp OR (child* OR infan* OR adolescen*):ab,ti) NOT (adult/exp OR (adult* OR elderl*):ab,ti))

Medline (Ovid)

(* quality of life/ OR ((quality ADJ3 life) OR qol).ti.) AND (* exp mental health/ OR * exp psychiatry/ OR * Diagnostic and Statistical Manual of Mental Disorders/ OR * exp Mental Disorders/ OR (((mental* OR psychic*) ADJ3 (health* OR disorder* OR ill*)) OR (mood ADJ3 disorder*) OR (obsessi* ADJ3 compuls*) OR dsm OR psychiatr* OR schizo* OR bipolar OR unipolar OR ((posttrauma* OR trauma*) ADJ3 stress) OR ptsd OR ((dissociati* OR somatoform* OR eating OR personalit* OR psychosexual* OR behav* OR psychosocial*) ADJ3 disorder*) OR (multiple ADJ3 personalit*) OR anorex* OR bulemi* OR addict* OR ((drug* OR alcohol* OR substance*) ADJ3 (depend* OR misuse OR abuse)) OR borderlin* OR depressi* OR anxi* OR panic OR (Diagnos* ADJ3 Statistic* ADJ3 Manual*) OR sexual* deviat* OR paraphil* OR psychosis OR psychoses OR psychotic OR phobia*).ti.) NOT (letter OR news OR comment OR editorial OR congresses OR abstracts).pt. AND english.la. NOT (cancer* OR malign* OR neoplas* OR hiv OR aids OR diabet* OR dement* OR cardiac* OR myocard* OR surg* OR postsurg* OR postoperat* OR stroke* OR

cva).ti. NOT ((exp child/ OR exp infant/ OR adolescent/ OR (child* OR infan* OR adolescen*).ab,ti.) NOT (exp adult/ OR (adult* OR elderl*).ab,ti.))

PsycINFO Ovid

(* "quality of life"/ OR ((quality ADJ3 life) OR qol).ti.) AND (* exp mental health/ OR * exp psychiatry/ OR * exp Mental Disorders/ OR (((mental* OR psychic*) ADJ3 (health* OR disorder* OR ill*)) OR (mood ADJ3 disorder*) OR (obsessi* ADJ3 compuls*) OR dsm OR psychiatr* OR schizo* OR bipolar OR unipolar OR ((posttrauma* OR trauma*) ADJ3 stress) OR ptsd OR ((dissociati* OR somatoform* OR eating OR personalit* OR psychosexual* OR behav* OR psychosocial*) ADJ3 disorder*) OR (multiple ADJ3 personalit*) OR anorex* OR bulemi* OR addict* OR ((drug* OR alcohol* OR substance*) ADJ3 (depend* OR misuse OR abuse)) OR borderlin* OR depressi* OR anxi* OR panic OR (Diagnos* ADJ3 Statistic* ADJ3 Manual*) OR sexual* deviat* OR paraphil* OR psychosis OR psychoses OR psychotic OR phobia*).ti.) NOT (letter OR news OR comment OR editorial OR congresses OR abstracts).pt. AND english.la. NOT (cancer* OR malign* OR neoplas* OR hiv OR aids OR diabet* OR dement* OR cardiac* OR myocard* OR surg* OR postsurg* OR postoperat* OR stroke* OR cva).ti. NOT ((100.ag. OR 200.ag. OR (child* OR infan* OR adolescen*).ab,ti.) NOT (300.ag. OR (adult* OR elderl*).ab,ti.))
