# Supplementary material for: Instruments to assess quality of life in people with mental health problems: a systematic review and dimension analysis of generic, domain- and disease-specific instruments
Source: Health Qual Life Outcomes. 2021 Nov 2;19:249. doi: 10.1186/s12955-021-01883-w (PMC8561965; doi:10.1186/s12955-021-01883-w)
Supplement: Supplementary file 2 — Additional file 2. Underlying themes. [file 12955_2021_1883_MOESM2_ESM.pdf]

Additional file 2 – Dimensions and underlying themes of quality of life that are important to people with mental health problems (Connell et al. 2014)

1. **WELL-BEING/ILL-BEING**

   - Distress/subjective experience of symptoms
   - Experience of psychosis/mania
   - Fear/Anxiety/Worry
   - Energy/motivation
   - Well-Being (positive concepts e.g. enjoyment/ relaxation/stability)

2. **RELATIONSHIPS AND BELONGING**

   - Belonging/being part of the community
   - Good relationships
     - Support
     - Acceptance/ understanding
     - Company/camaraderie/shared interests
     - Love, care and affection
   - Difficulties forming and maintaining relationships
   - Stigma
   - Feeling normal
   - Loneliness/Isolation, alienation

3. **ACTIVITY**

   - Activity general
   - Employment
   - Meaningful/enjoyable/suited to needs
   - Routine and structure

4. **SELF-PERCEPTION**

   - Self-identity/sense of self
   - Self-efficacy
   - Self-esteem
   - Self-acceptance/self-stigma

5. **AUTONOMY**

   - Control (general)
   - Symptom control/management
   - Information and understanding of illness
   - Choice (general)
   - Choice - related to limited finances
   - Choice - related to job opportunities
   - Independence/dependence
   - Personal strength, determination, self sufficiency

6. HOPE AND HOPELESSNESS

- Hope/hopelessness
- Goals/personal achievement
- Hope and Hopelessness
- Loss and effect of past experiences

7. PHYSICAL HEALTH
