# Supplementary material for: Instruments to assess quality of life in people with mental health problems: a systematic review and dimension analysis of generic, domain- and disease-specific instruments
Source: Health Qual Life Outcomes. 2021 Nov 2;19:249. doi: 10.1186/s12955-021-01883-w (PMC8561965; doi:10.1186/s12955-021-01883-w)
Supplement: Supplementary file 3 — Additional file 3. References of included studies. [file 12955_2021_1883_MOESM3_ESM.pdf]

Additional file 3 - References of included papers

1. Aardoom, J., Dingemans, A., van Ginkel, J., Spinhoven, P., Van Furth, E., & Van den Akker-van Marle, M. (2016). Cost-utility of an internet-based intervention with or without therapist support in comparison with a waiting list for individuals with eating disorder symptoms: A randomized controlled trial.*49*, 1068-1076.
2. Abbaszadeh, F., Kafaei Atrian, M., Masoudi Alavi, N., Bagheri, A., Sadat, Z., & Karimian, Z. (2013). *Relationship between quality of life and depression in pregnant women.*
3. Abbate-Daga, G., Facchini, F., Marzola, E., Delsedime, N., Giovannone, C., Amianto, F., & Fassino, S. (2014). Health-related quality of life in adult inpatients affected by anorexia nervosa.*22*, 285-291.
4. Abedi Shargh, N., Rostami, B., Kosari, B., Toosi, Z., & Majelan, G. A. (2015). Study of relationship between depression and quality of life in patients with chronic schizophrenia.*8*, 224-229.
5. Abraham, K. M., Miller, C. J., Birgenheir, D. G., Lai, Z., & Kilbourne, A. M. (2014). Self-efficacy and quality of life among people with bipolar disorder.*202*, 583-588.
6. Ackard, D. M., Richter, S., Egan, A., Engel, S., & Cronemeyer, C. L. (2014). The meaning of (quality of) life in patients with eating disorders: A comparison of generic and disease-specific measures across diagnosis and outcome.*47*, 259-267.
7. Ackard, D. M., Richter, S. A., Egan, A. M., & Cronemeyer, C. L. (2014). What does remission tell us about women with eating disorders? investigating applications of various remission definitions and their associations with quality of life.*76*, 12-18.
8. Adams, R. E., Ritter, C., & Bonfine, N. (2015). Epidemiology of trauma: Childhood adversities, neighborhood problems, discrimination, chronic strains, life events, and daily hassles among people with a severe mental illness.*230*, 609-615.
9. Addington, D. E., McKenzie, E., & Wang, J. (2012). Validity of hospital admission as an outcome measure of services for first-episode psychosis.*63*, 280-282.
10. Adelufosi, A. O., Adebowale, T. O., Abayomi, O., & Mosanya, J. T. (2012). Medication adherence and quality of life among nigerian outpatients with schizophrenia.*34*, 72-79.

11. Adelufosi, A. O., Ogunwale, A., Abayomi, O., & Mosanya, J. T. (2013). Socio-demographic and clinical correlates of subjective quality of life among nigerian outpatients with schizophrenia.*209*, 320-325.

12. Adler, L. A., Dirks, B., Deas, P., Raychaudhuri, A., Dauphin, M., Saylor, K., & Weisler, R. (2013). Self-reported quality of life in adults with attention-deficit/hyperactivity disorder and executive function impairment treated with lisdexamfetamine dimesylate: A randomized, double-blind, multicenter, placebo-controlled, parallel-group study.*13*

13. Afe, T. O., Bello-Mojeed, M., & Ogunsemi, O. (2016). Perception of service satisfaction and quality of life of patients living with schizophrenia in lagos, nigeria.*7*, 216-222.

14. Agdal, M. L., Raadal, M., √ñst, L. G., & Skaret, E. (2012). Quality-of-life before and after cognitive behavioral therapy (CBT) in patients with intra-oral injection phobia.*70*, 463-470.

15. Aggarwal, S., Kataria, D., & Prasad, S. (2017). *A comparative study of quality of life and marital satisfaction in patients with depression and their spouses*

16. Agibalova, T. V., & Kozin, V. A. (2016). Emotional burnout syndrome in narcologists and its effects on the quality of life of patients with alcohol dependence.*46*, 758-761.

17. Agorastos, A., Pittman, J. O. E., Angkaw, A. C., Nievergelt, C. M., Hansen, C. J., Aversa, L. H., . . . Baker, D. G. (2014). The cumulative effect of different childhood trauma types on self-reported symptoms of adult male depression and PTSD, substance abuse and health-related quality of life in a large active-duty military cohort.*58*, 46-54.

18. Ahmadi, K., Shahidi, S., Nejati, V., Karami, G., & Masoomi, M. (2013). Effects of chronic illness on the quality of life in psychiatric out patients of the iraq-iran war.*8*, 7-13.

19. Ahmadizadeh, M., Ahmadi, K., Anisi, J., & Ahmadi, A. B. (2013). Assessment of cognitive behavioral therapy on quality of life of patients with chronic war-related post-traumatic stress disorder.*35*, 341-345.

20. Akinsulore, A., Aloba, O. O., Mapayi, B. M., Oloniniyi, I. O., Fatoye, F. O., & Makanjuola, R. O. (2014). Relationship between depressive symptoms and quality of life in nigerian patients with schizophrenia.*49*, 1191-1198.

21. Akosile, C. O., Mgbeojedo, U. G., Maruf, F. A., Okoye, E. C., Umeonwuka, I. C., & Ogunniyi, A. (2018). Depression, functional disability and quality of life among nigerian older adults: Prevalences and relationships.*74*, 39-43.

22. Alessandrini, M., LanÃ§on, C., Fond, G., Faget-Agius, C., Richieri, R., Faugere, M., . . . Boyer, L. (2016). A structural equation modelling approach to explore the determinants of quality of life in schizophrenia.*171*, 27-34.

23. Alexinschi, O., Chirita, R., Manuela, P., Ciobica, A., Dobrin, R., Petrariu, F. D., . . . Chirita, V. (2015). Additional demographic and clinical evidences on the relevance of the systemic therapy in alcohol dependence.*119*, 1120-1127.

24. Alm, C., Eriksson, A., Durbeej, N., Palmstierna, T., Berman, A. H., Kristiansson, M., & Gumpert, C. H. (2014). Classification of offenders with mental health problems and problematic substance use using the addiction severity index version 6: Analysis of three-year follow-up data and predictive validity.*7*, 431-445.

25. Alminhana, L. O., Farias, M., Claridge, G., Cloninger, C. R., & Moreira-Almeida, A. (2017). Self-directedness predicts quality of life in individuals with psychotic experiences: A 1-year follow-up study.*50*, 239-245.

26. Al-Smadi, A. M., Tawalbeh, L. I., Gammoh, O. S., Ashour, A. F., Alshraifeen, A., & Gougazeh, Y. M. (2017). Anxiety, stress, and quality of life among iraqi refugees in jordan: A cross sectional survey.*19*, 100-104.

27. Amini, H., & Sharifi, V. (2012). Quality of life in bipolar type I disorder in a one-year followup.*2012*, 860745.

28. An, F. R., Yang, R., Wang, Z. M., Ungvari, G. S., Ng, C. H., Chiu, H. F. K., . . . Xiang, Y. T. (2016). Hyperprolactinemia, prolactin-related side effects and quality of life in chinese psychiatric patients.*71*, 71-76.

29. An, F. R., Zhang, L., Zhang, Q. E., Ungvari, G. S., Ng, C. H., Chiu, H. F. K., . . . Xiang, Y. T. (2016). Electroconvulsive therapy and its relationships with clinical characteristics and quality of life in chinese psychiatric patients.*246*, 246-249.

30. An, S. Y., Kim, G. H., & Kim, J. Y. (2017). Effectiveness of shared decision-making training program in people with schizophrenia in south korea.*53*, 111-118.

31. Anaya, C., Torrent, C., Caballero, F. F., Vieta, E., Bonnin, C. d. M., Ayuso-Mateos, J. L., . . . Vega, P. (2016). Cognitive reserve in bipolar disorder: Relation to cognition, psychosocial functioning and quality of life.*133*, 386-398.

32. Andrade, L. F., Alessi, S. M., & Petry, N. M. (2012). The impact of contingency management on quality of life among cocaine abusers with and without alcohol dependence.*21*, 47-54.

33. Andreou, C., Treszl, A., Roesch-Ely, D., Kother, U., Veckenstedt, R., & Moritz, S. (2014). Investigation of the role of the jumping-to-conclusions bias for short-term functional outcome in schizophrenia.*218*, 341-347.

34. Andrianarisoa, M., Boyer, L., Godin, O., Brunel, L., Bulzacka, E., Aouizerate, B., . . . Zinetti-Bertschy, A. (2017). Childhood trauma, depression and negative symptoms are independently associated with impaired quality of life in schizophrenia. results from the national FACE-SZ cohort.*185*, 173-181.

35. Angelovski, A., Sattel, H., Henningsen, P., & Sack, M. (2016). Heart rate variability predicts therapy outcome in pain-predominant multisomatoform disorder.*83*, 16-21.

36. Angkaw, A. C., Haller, M., Pittman, J. O., Nunnink, S. E., Norman, S. B., Lemmer, J. A., . . . Baker, D. G. (2015). Alcohol-related consequences mediating PTSD symptoms and mental health-related quality of life in OEF/OIF combat veterans.*180*, 670-675.

37. Antshel, K. M., Kaul, P., Biederman, J., Spencer, T. J., Hier, B. O., Hendricks, K., & Faraone, S. V. (2013). Posttraumatic stress disorder in adult attention-deficit/hyperactivity disorder: Clinical features and familial transmission.*74*, e197-e204.

38. ÃœÃ§ok, A., Gorwood, P., & Karadayi, G. (2012). Employment and its relationship with functionality and quality of life in patients with schizophrenia: EGOFORS study.*27*, 422-425.

39. ÃœÃ§ok, A., Karadayi, G., EmiroÇ§lu, B., & Sartorius, N. (2013). Anticipated discrimination is related to symptom severity, functionality and quality of life in schizophrenia.*209*, 333-339.

40. ÃœÃ§ok, A., Tihan, A. K., Karadayi, G., & TÃ□kel, R. (2014). Obsessive compulsive symptoms are related to lower quality of life in patients with schizophrenia.*18*, 243-247.

41. AraÃºjo, A. X., Berger, W., Coutinho, E. S. F., Marques-Portella, C., Luz, M. P., Cabizuca, M., . . . Mendlowicz, M. V. (2014). Comorbid depressive symptoms in treatment-seeking PTSD outpatients affect multiple domains of quality of life.*55*, 56-63.

42. Arab, M., Kohan, M., Ranjbar, H., Arab, N., Rayani, M., Mirrashidi, S. S., . . . Amiri, M. (2014). Quality of life, social desirability and their relationship in opium addicted persons in southeast of iran.*6*, 97-103.

43. Aras, H. I., Yazar, M. S., & AltinbaÅŸ, K. (2013). Quality of life among dually diagnosed and non-substance-using male schizophrenia outpatients.*19*, 35-39.

44. Arch, J. J., Eifert, G. H., Davies, C., Plumb Vilardaga, J. C., Rose, R. D., & Craske, M. G. (2012). Randomized clinical trial of cognitive behavioral therapy (CBT) versus acceptance and commitment therapy (ACT) for mixed anxiety disorders.*80*, 750-765.

45. Årdal, G., Lund, A., & Hammar, Ã. (2013). Health-related quality of life in recurrent major depressive disorder - A 10-year follow-up study.*67*, 339-343.

46. Areberg, C., & Bejerholm, U. (2013). The effect of IPS on participants' engagement, quality of life, empowerment, and motivation: A randomized controlled trial.*20*, 420-428.

47. Arvidsdotter, T., Marklund, B., Taft, C., & KylÃ©n, S. (2015). Quality of life, sense of coherence and experiences with three different treatments in patients with psychological distress in primary care: A mixed-methods study.*15*

48. Ascher-Svanum, H., Novick, D., Haro, J. M., Aguado, J., & Cui, Z. (2013). Empirically driven definitions of "good," "moderate," and "poor" levels of functioning in the treatment of schizophrenia.*22*, 2085-2094.

49. Asnaani, A., Kaczkurkin, A. N., Alpert, E., McLean, C. P., Simpson, H. B., & Foa, E. B. (2017). The effect of treatment on quality of life and functioning in OCD.*73*, 7-14.

50. Aspis, I., Feingold, D., Weiser, M., Rehm, J., Shoval, G., & Lev-Ran, S. (2015). Cannabis use and mental health-related quality of life among individuals with depressive disorders.*230*, 341-349.

51. Å□witaj, P., Grygiel, P., Chrostek, A., Nowak, I., WciÃ□rka, J., & Anczewska, M. (2017). The relationship between internalized stigma and quality of life among people with mental illness: Are self-esteem and sense of coherence sequential mediators?*26*, 2471-2478.

52. Aubry, T., Goering, P., Veldhuizen, S., Adair, C. E., Bourque, J., Distasio, J., . . . Tsemberis, S. (2016). A multiple-city RCT of housing first with assertive community treatment for homeless canadians with serious mental illness.*67*, 275-281.

53. Auquier, P., Tinland, A., Fortanier, C., Loundou, A., Baumstarck, K., Lancon, C., & Boyer, L. (2013). Toward meeting the needs of homeless people with schizophrenia: The validity of quality of life measurement.*8*

54. Aversa, L. H., Stoddard, J. A., Doran, N. M., Au, S., Chow, B., McFall, M., . . . Baker, D. G. (2012). PTSD and depression as predictors of physical health-related quality of life in tobacco-dependent veterans.*73*, 185-190.

55. Aversa, L. H., Stoddard, J. A., Doran, N. M., Au, S., Chow, B., McFall, M., . . . Baker, D. G. (2013). Longitudinal analysis of the relationship between PTSD symptom clusters, cigarette use, and physical health-related quality of life.*22*, 1381-1389.

56. Avery, J. C., Stocks, N. P., Duggan, P., Braunack-Mayer, A. J., Taylor, A. W., Goldney, R. D., & Maclennan, A. H. (2013). Identifying the quality of life effects of urinary incontinence with depression in an australian population.*13*

57. Awad, G., Hassan, M., Loebel, A., Hsu, J., Pikalov, A., & Rajagopalan, K. (2014). Health-related quality of life among patients treated with lurasidone: Results from a switch trial in patients with schizophrenia.*14*

58. Awad, G., Ng-Mak, D., Rajagopalan, K., Hsu, J., Pikalov, A., & Loebel, A. (2016). Long-term health-related quality of life improvements among patients treated with lurasidone: Results from the open-label extension of a switch trial in schizophrenia.*16*

59. BaÅŸar, K., Ã–z, G., & Karakaya, J. (2016). Perceived discrimination, social support, and quality of life in gender dysphoria.*13*, 1133-1141.

60. Babaie, E., & Razeghi, N. (2013). Comparing the effects of methadone maintenance treatment, therapeutic community, and residential rehabilitation on quality of life and mental health of drug addicts.*5*, 16-20.

61. Bagheri, M., Mokri, A., Khosravi, A., & Kabir, K. (2015). Effect of abstinence on depression, anxiety, and quality of life in chronic methamphetamine users in a therapeutic community.*4*, e23903.

62. Baharom, N., Hassan, M. R., Ali, N., & Shah, S. A. (2012). Improvement of quality of life following 6 months of methadone maintenance therapy in malaysia.*7*, 32.

63. Baiano, M., Salvo, P., Righetti, P., Cereser, L., Baldissera, E., Camponogara, I., & Balestrieri, M. (2014). Exploring health-related quality of life in eating disorders by a cross-sectional study and a comprehensive review review.*14*, 165.

64. Bajor, L. A., Lai, Z., Goodrich, D. E., Miller, C. J., Penfold, R. B., Myra Kim, H., . . . Kilbourne, A. M. (2013). Posttraumatic stress disorder, depression, and health-related quality of life in patients with bipolar disorder: Review and new data from a multi-site community clinic sample.*145*, 232-239.

65. Bambini, V., Arcara, G., Bechi, M., Buonocore, M., Cavallaro, R., & Bosia, M. (2016). The communicative impairment as a core feature of schizophrenia: Frequency of pragmatic deficit, cognitive substrates, and relation with quality of life.*71*, 106-120.

66. Bamford, B., Barras, C., Sly, R., Stiles-Shields, C., Touyz, S., Le Grange, D., . . . Lacey, H. (2015). Eating disorder symptoms and quality of life: Where should clinicians place their focus in severe and enduring anorexia nervosa?*48*, 133-138.

67. Barahmand, U., Tavakolian, E., & Alaei, S. (2014). Association of metacognitive beliefs, obsessive beliefs and symptom severity with quality of life in obsessive-compulsive patients.*28*, 345-351.

68. Barnes, A. L., Murphy, M. E., Fowler, C. A., & Rempfer, M. V. (2012). Health-related quality of life and overall life satisfaction in people with serious mental illness.

69. Barrera, T. L., Hiatt, E. L., Dunn, N. J., & Teng, E. J. (2013). Impact of panic disorder on quality of life among veterans in a primary care pilot study.*54*, 256-261.

70. Battersby, M. W., Beattie, J., Pols, R. G., Smith, D. P., Condon, J., & Blunden, S. (2013). A randomised controlled trial of the flinders ProgramTM of chronic condition management in vietnam veterans with co-morbid alcohol misuse, and psychiatric and medical conditions.*47*, 451-462.

71. Baumgartner, J. N., & Herman, D. B. (2012). Community integration of formerly homeless men and women with severe mental illness after hospital discharge.*63*, 435-437.

72. Baumstarck, K., Boyer, L., Boucekine, M., Aghababian, V., Parola, N., LanÃ§on, C., & Auquier, P. (2013). Self-reported quality of life measure is reliable and valid in adult patients suffering from schizophrenia with executive impairment.*147*, 58-67.

73. Benaiges, I., Prat, G., & Adan, A. (2012). Health-related quality of life in patients with dual diagnosis: Clinical correlates.*10*

74. Ben-Zur, H., Duvdevany, I., & Saffoury Issa, D. (2014). Ethnicity moderates the effects of resources on quality of life for persons with mental illness living in community settings.*37*, 309-315.

75. Berghoff, C. R., Forsyth, J. P., Ritzert, T. R., & Sheppard, S. C. (2014). Comparing paths to quality of life: Contributions of ACT and cognitive therapy intervention targets in two highly anxious samples.*3*, 89-97.

76. Bernardi, S., Faraone, S. V., Cortese, S., Kerridge, B. T., Pallanti, S., Wang, S., & Blanco, C. (2012). The lifetime impact of attention deficit hyperactivity disorder: Results from the national epidemiologic survey on alcohol and related conditions (NESARC).*42*, 875-887.

77. Bevanda, D., TomiÄ‡, I., Bevanda, M., SkoÄ□ibuÅ¡iÄ‡, S., Palameta, N., & Martinac, M. (2017). The differences in quality of life between the heroin addicts treated in methadone program and addicts treated in the frame of therapeutic community program.*53*, 17-26.

78. Biesheuvel-Leliefeld, K. E., Kok, G. D., Bockting, C. L., de Graaf, R., ten Have, M., van der Horst, Henriette E., . . . Smit, F. (2016). Non-fatal disease burden for subtypes of depressive disorder: Population-based epidemiological study.*16*

79. Biggs, Q. M., Fullerton, C. S., McCarroll, J. E., Liu, X., Wang, L., Dacuyan, N. M., . . . Ursano, R. J. (2016). Early intervention for post-traumatic stress disorder, depression, and quality of life in mortuary affairs soldiers postdeployment.*181*, e1553-e1560.

80. Bilbao, A., Las Hayas, C., Forero, C. G., Padierna, A., Martin, J., & Quintana, J. M. (2014). Cross-validation study using item response theory: The health-related quality of life for eating disorders questionnaire-short version.*21*, 477-493.

81. Bishop-Fitzpatrick, L., Smith DaWalt, L., Greenberg, J. S., & Mailick, M. R. (2017). Participation in recreational activities buffers the impact of perceived stress on quality of life in adults with autism spectrum disorder.*10*, 973-982.

82. Black, D. W., Shaw, M., McCormick, B., & Allen, J. (2013). Pathological gambling: Relationship to obesity, self-reported chronic medical conditions, poor lifestyle choices, and impaired quality of life.*54*, 97-104.

83. Bokhari, S. Q., Bokhari, Q. M., Mariam, A., & Majeed, R. (2015). Correlation between quality of life and positive and negative symptoms of schizophrenia.*9*, 367-370.

84. Bold, K. W., Epstein, E. E., & McCrady, B. S. (2017). Baseline health status and quality of life after alcohol treatment for women with alcohol dependence.*64*, 35-41.

85. Bolek, S., Yargic, I., & Ekinci, O. (2016). The effects of buprenorphine/naloxane maintenance treatment on the quality of life, substance use and functionality in opiate dependence: A follow-up study.*26*, 141-151.

86. Bonn√≠n, C. M., S√°nchez-Moreno, J., Mart√≠nez-Ar√°n, A., Sol√©, B., Reinares, M., Rosa, A. R., . . . Torrent, C. (2012). Subthreshold symptoms in bipolar disorder: Impact on neurocognition, quality of life and disability.*136*, 650-659.

87. Bonsaksen, T., & Lerdal, A. (2012). Relationships between physical activity, symptoms and quality of life among inpatients with severe mental illness.*75*, 69-75.

88. Borges, T. L., Miasso, A. I., Reisdofer, E., Dos Santos, M. A., Vedana, K. G. G., & Hegadoren, K. M. (2016). Common mental disorders in primary health care units: Associated factors and impact on quality of life.*22*, 378-386.

89. Boucekine, M., Boyer, L., Baumstarck, K., Millier, A., Ghattas, B., Auquier, P., & Toumi, M. (2015). Exploring the response shift effect on the quality of life of patients with schizophrenia: An application of the random forest method.*35*, 388-397.

90. Bourion-BÃ©dÃ¨s, S., Schwan, R., Epstein, J., Laprevote, V., BÃ©dÃ¨s, A., Bonnet, J. L., & Baumann, C. (2015). Combination of classical test theory (CTT) and item response theory (IRT) analysis to study the psychometric properties of the french version of the quality of life enjoyment and satisfaction questionnaire-short form (Q-LES-Q-SF).*24*, 287-293.

91. Bouwmans, C. A. M., Vemer, P., Van Straten, A., Tan, S. S., & Hakkaart-Van Roijen. (2014). Health-related quality of life and productivity losses in patients with depression and anxiety disorders.*56*, 420-424.

92. Bowersox, N. W., Lai, Z., & Kilbourne, A. M. (2012). Integrated care, recovery-consistent care features, and quality of life for patientswith serious mental illness.*63*, 1142-1145.

93. Boyer, L., Aghababian, V., Richieri, R., Loundou, A., Padovani, R., Simeoni, M. C., . . . Lan√ßon, C. (2012). Insight into illness, neurocognition and quality of life in schizophrenia.*36*, 271-276.

94. Boyer, L., LancoÌ§n, C., Baumstarck, K., Parola, N., Berbis, J., & Auquier, P. (2013). Evaluating the impact of a quality of life assessment with feedback to clinicians in patients with schizophrenia: Randomised controlled trial.*202*, 447-453.

95. Boyer, L., Millier, A., Perthame, E., Aballea, S., Auquier, P., & Toumi, M. (2013). Quality of life is predictive of relapse in schizophrenia.*13*

96. Boyer, L., Richieri, R., Faget, C., Padovani, R., Vaillant, F., Mundler, O., . . . Guedj, E. (2012). Functional involvement of superior temporal sulcus in quality of life of patients with schizophrenia.*202*, 155-160.

97. Boyer, L., Richieri, R., Guedj, E., Faget-Agius, C., Loundou, A., Llorca, P. M., . . . Lancon, C. (2013). Validation of a functional remission threshold for the functional remission of general schizophrenia (FROGS) scale.*54*, 1016-1022.

98. Boyette, L. L., Korver-Nieberg, N., Meijer, C., De Haan, L., Kahn, R. S., Van Os, J., . . . Myin-Germeys, I. (2014). Quality of life in patients with psychotic disorders: Impact of symptoms, personality, and attachment.*202*, 64-69.

99. Boyette, L. L., van Dam, D., Meijer, C., Velthorst, E., Cahn, W., de Haan, L., . . . Myin-Germeys, I. (2014). Personality compensates for impaired quality of life and social functioning in patients with psychotic disorders who experienced traumatic events.*40*, 1356-1365.

100. Breitborde, N. J. K., Kleinlein, P., & Srihari, V. H. (2012). Self-determination and first-episode psychosis: Associations with symptomatology, social and vocational functioning, and quality of life.*137*, 132-136.

101. Brenes, G. A., Danhauer, S. C., Lyles, M. F., Anderson, A., & Miller, M. E. (2016). Effects of telephone-delivered cognitive-behavioral therapy and nondirective supportive therapy on sleep, health-related quality of life, and disability.*24*, 846-854.

102. Bressington, D., Mui, J., Tse, M. L., Gray, R., Cheung, E. F. C., & Chien, W. T. (2016). Cardiometabolic health, prescribed antipsychotics and health-related quality of life in people with schizophrenia-spectrum disorders: A cross-sectional study.*16*

103. Briki, M., Monnin, J., Haffen, E., Sechter, D., Favrod, J., Netillard, C., . . . Vandel, P. (2014). Metacognitive training for schizophrenia: A multicentre randomised controlled trial.*157*, 99-106.

104. Brod, M., Adler, L. A., Lipsius, S., Tanaka, Y., Heinloth, A. N., & Upadhyaya, H. (2015). Validation of the adult attention-deficit/hyperactivity disorder quality-of-life scale in european patients: Comparison with patients from the USA.*7*, 141-150.

105. Brod, M., Schmitt, E., Goodwin, M., Hodgkins, P., & Niebler, G. (2012). ADHD burden of illness in older adults: A life course perspective.*21*, 795-799.

106. Brosey, E., & Woodward, N. D. (2015). Schizotypy and clinical symptoms, cognitive function, and quality of life in individuals with a psychotic disorder.*166*, 92-97.

107. Brown, J. L., Eubanks, C., & Keating, A. (2017). Yoga, quality of life, anxiety, and trauma in low-income adults with mental illness: A mixed-methods study.*15*, 308-330.

108. Brown, S., Jun, M. K., Min, M. O., & Tracy, E. M. (2013). Impact of dual disorders, trauma, and social support on quality of life among women in treatment for substance dependence.*9*, 61-71.

109. Brown, S., Victor, B., Hicks, L. M., & Tracy, E. M. (2017). Recovery support mediates the relationship between parental warmth and quality of life among women with substance use disorders.*26*, 1327-1335.

110. Bryant, R. A., McFarlane, A. C., Silove, D., O'Donnell, M. L., Forbes, D., & Creamer, M. (2016). The lingering impact of resolved PTSD on subsequent functioning.*4*, 493-498.

111. Bueno, V. F., Kozasa, E. H., Da Silva, M. A., Alves, T. M., LouzÃ£, M. R., & PompeiÃ¡, S. (2015). Mindfulness meditation improves mood, quality of life, and attention in adults with attention deficit hyperactivity disorder.*2015*

112. Buhmann, C., Mortensen, E. L., Nordentoft, M., Ryberg, J., & EkstrÃ¸m, M. (2015). Follow-up study of the treatment outcomes at a psychiatric trauma clinic for refugees.*25*, 1-16.

113. Buonocore, M., Bosia, M., Bechi, M., Spangaro, M., Cavedoni, S., Cocchi, F., . . . Cavallaro, R. (2017). Targeting anxiety to improve quality of life in patients with schizophrenia.*45*, 129-135.

114. Buonocore, M., Spangaro, M., Bechi, M., Baraldi, M. A., Cocchi, F., Guglielmino, C., . . . Cavallaro, R. (2017). Integrated cognitive remediation and standard rehabilitation therapy in patients of schizophrenia: Persistence after 5years.

115. Buonocore, M., Bosia, M., Bechi, M., Spangaro, M., Cavedoni, S., Cocchi, F., . . . Cavallaro, R. (2017). Is longer treatment better? A comparison study of 3 versus 6 months cognitive remediation in schizophrenia.*31*, 467-473.

116. Burger, P. H. M., Neumann, C., Ropohl, A., Paulsen, F., & Scholz, M. (2016). Development of depression and deterioration in quality of life in german dental medical students in preclinical semesters.*208*, 183-186.

117. Burns-Lynch, B., Brusilovskiy, E., & Salzer, M. S. (2016). An empirical study of the relationship between community participation, recovery, and quality of life of individuals with serious mental illnesses.*53*, 46-54.

118. Cai, C., & Yu, L. (2017). Quality of life in patients with schizophrenia in china: Relationships among demographic characteristics, psychosocial variables, and symptom severity.*55*, 48-54.

119.Calabrese, J., Rajagopalan, K., Ng-Mak, D., Bacci, E. D., Wyrwich, K., Pikalov, A., & Loebel, A. (2016). Effect of lurasidone on meaningful change in health-related quality of life in patients with bipolar depression.*31*, 147-154.

120.Caldirola, D., Grassi, M., Riva, A., DaccÃ□, S., De Berardis, D., Dal Santo, B., & Perna, G. (2014). Self-reported quality of life and clinician-rated functioning in mood and anxiety disorders: Relationships and neuropsychological correlates.*55*, 979-988.

121.Callegari, C., Berto, E., Caselli, I., Bressani, R., & Vender, S. (2016). Multidisciplinary integrated approach to mental illness: Semi-residential setting and quality of life.*57*, 34-41.

122.Cao, X. L., Chiu, H. F. K., Yim, L. C. L., & Lin, Y. Q. (2017). Comparison of quality of life in homeless and non-homeless chinese patients with psychiatric disorders.*249*, 115-119.

123.Cao, Y., Li, W., Shen, J., Malison, R. T., Zhang, Y., & Luo, X. (2013). Health-related quality of life and symptom severity in chinese patients with major depressive disorder.*5*, 276-283.

124.Caqueo-UrÃzar, A., Boyer, L., Baumstarck, K., & Gilman, S. E. (2015). Subjective perceptions of cognitive deficits and their influences on quality of life among patients with schizophrenia.*24*, 2753-2760.

125.Caqueo-UrÃzar, A., Boyer, L., Boucekine, M., & Auquier, P. (2014). Spanish cross-cultural adaptation and psychometric properties of the schizophrenia quality of life short-version questionnaire (SQoL18) in 3 middle-income countries: Bolivia, chile and peru.

126.Caqueo-UrÃzar, A., GutiÃ©rrez-Maldonado, J., Ferrer-GarcÃa, M., Morales, A. U., & FernÃ¡ndez-DÃ¡vila, P. (2013). Typology of schizophrenic symptoms and quality of life in patients and their main caregivers in northern chile.*59*, 93-100.

127.Caqueo-UrÃzar, A., UrzÃºa, A., Boyer, L., & Williams, D. R. (2016). Religion involvement and quality of life in patients with schizophrenia in latin america.*51*, 521-528.

128.Caqueo-Urizar, A., Gutierrez-Maldonado, J., Ferrer-Garcia, M., & Fernandez-Davila, P. (2012). Quality of life of schizophrenia patients of aymaran ethnic background in the north of chile.*5*, 121-126.

129.Cardoso, T. D. A., Farias, C. D. A., Mondin, T. C., da Silva, G. D. G., Souza, L. D. D. M., da Silva, R. A., . . . Jansen, K. (2014). Brief psychoeducation for bipolar disorder: Impact on quality of life in young adults in a 6-month follow-up of a randomized controlled trial.*220*, 896-902.

130. Carlier, M., Mainguet, B., & Delevoye-Turrell, Y. (2016). Cognitive exercise through body movement: Using a fun and short neuropsychological tool to adapt physical activity and enhance pleasure in individuals suffering from mental illnesses.*61*, 349-359.

131. CarrÃ , G., Johnson, S., Crocamo, C., Angermeyer, M. C., Brugha, T., Azorin, J. M., . . . Bebbington, P. E. (2016). Psychosocial functioning, quality of life and clinical correlates of comorbid alcohol and drug dependence syndromes in people with schizophrenia across europe.*239*, 301-307.

132. Carrera, I., SÃ¡nchez, L., Sabater, E., Pereiro, C., FlÃ□rez, G., Conde, M., . . . Casado, M. Ã. (2016). Study on usersâ€™ perception of agonist opioid treatment in the galician network of drug addiction.*18*, 5-8.

133. Carta, M. G., Maggiani, F., Pilutzu, L., Moro, M. F., Mura, G., Sancassiani, F., . . . Preti, A. (2014). Sailing can improve quality of life of people with severe mental disorders: Results of a cross over randomized controlled trial.*10*, 80-86.

134. Carta, M. G., Moro, M. F., Aguglia, E., Balestrieri, M., Caraci, F., Dell'Osso, L., . . . Faravelli, C. (2015). The attributable burden of panic disorder in the impairment of quality of life in a national survey in italy.*61*, 693-699.

135. Carta, M. G., Norcini-Pala, A., Moro, M. F., Balestrieri, M., Caraci, F., Dell'Osso, L., . . . Drago, F. (2015). Does mood disorder questionnaire identify sub-threshold bipolarity? evidence studying worsening of quality of life.*183*, 173-178.

136. Carta, M. G., Petretto, D., Adamo, S., Bhat, K. M., Lecca, M. E., Mura, G., . . . Moro, M. F. (2012). Counseling in primary care improves depression and quality of life.*8*, 152-157.

137. Carta, M. G., Preti, A., Moro, M. F., Aguglia, E., Balestrieri, M., Caraci, F., . . . Bhugra, D. (2014). Eating disorders as a public health issue: Prevalence and attributable impairment of quality of life in an italian community sample.*26*, 486-492.

138. Castellini, G., Fioravanti, G., Ravaldi, C., Masetti, S., Vannacci, A., Mannucci, E., . . . Ricca, V. (2013). The eating disorders well being questionnaire (EDwell): A new measure of quality of life in eating disorders.*18*, 11-22.

139. Cerne, A., Rifel, J., Rotar-Pavlic, D., Svab, I., Selic, P., & Kersnik, J. (2013). Quality of life in patients with depression, panic syndrome, other anxiety syndrome, alcoholism and chronic somatic diseases: A longitudinal study in slovenian primary care patients.*125*, 1-7.

140. Chamberlain, S. R., Leppink, E. W., Redden, S. A., & Grant, J. E. (2016). Are obsessive-compulsive symptoms impulsive, compulsive or both?*68*, 111-118.

141. Chang, K. C., & Lin, C. Y. (2015). Effects of publicly funded and quality of life on attendance rate among methadone maintenance treatment patients in taiwan: An 18-month follow-up study.*12*

142. Chang, K. J., Kim, K., Fava, M., Mischoulon, D., Hong, J. P., Kim, D. J., . . . Jeon, H. J. (2016). Cross-national differences in hypochondriasis symptoms between korean and american outpatients with major depressive disorder.*245*, 127-132.

143. Chang, L. R., Lin, Y. H., Wu Chang, H. C., Chen, Y. Z., Huang, W. L., Liu, C. M., . . . Hwu, H. G. (2013). Psychopathology, rehospitalization and quality of life among patients with schizophrenia under home care case management in taiwan.*112*, 208-215.

144. Chang, W. C., Chan, T. C. W., Chen, E. S. M., Hui, C. L. M., Wong, G. H. Y., Chan, S. K. W., . . . Chen, E. Y. H. (2013). The concurrent and predictive validity of symptomatic remission criteria in first-episode schizophrenia.*143*, 107-115.

145. Chang, W. C., Cheung, R., Hui, C. L. M., Lin, J., Chan, S. K. W., Lee, E. H. M., & Chen, E. Y. H. (2015). Rate and risk factors of depressive symptoms in chinese patients presenting with first-episode non-affective psychosis in hong kong.*168*, 99-105.

146. Chang, Y. C., Ouyang, W. C., Lu, M. C., Wang, J. D., & Hu, S. C. (2016). Levels of depressive symptoms may modify the relationship between the WHOQOL-BREF and its determining factors in community-dwelling older adults.*28*, 591-601.

147. Chatterjee, S. S., Mitra, S., Guha, P., & Chakraborty, K. (2015). Prevalence of restless legs syndrome in somatoform pain disorder and its effect on quality of life.*6*, 160-164.

148. Chaves, K. M., Serrano-Blanco, A., Ribeiro, S. B., Soares, L. A. L., Guerra, G. C. B., Do Socorro Costa Feitosa Alves, M., . . . De AraÃºjo, A. A. (2013). Quality of life and adverse effects of olanzapine versus risperidone therapy in patients with schizophrenia.*84*, 125-135.

149. Chen, E. S. M., Chang, W. C., Hui, C. L. M., Chan, S. K. W., Lee, E. H. M., & Chen, E. Y. H. (2016). Self-stigma and affiliate stigma in first-episode psychosis patients and their caregivers.*51*, 1225-1231.

150. Chen, M. T., Li, C. Y., Lin, H. C., Shen, W. W., Hsieh, P. C., & Chen, C. C. (2013). Health-seeking behavior, alternative medicine, and quality of life in taiwanese panic disorder patients.*17*, 206-215.

151. Chen, V. C. H., Ting, H., Wu, M. H., Lin, T. Y., & Gossop, M. (2017). Sleep disturbance and its associations with severity of dependence, depression and quality of life among heroin-dependent patients: A cross-sectional descriptive study.*12*, 16.

152. Chen, Y. L., Pan, A. W., Hsiung, P. C., & Chung, L. (2015). Quality of life enhancement programme for individuals with mood disorder: A randomized controlled pilot study.*25*, 23-31.

153. Chen, Y. Z., Huang, W. L., Shan, J. C., Lin, Y. H., Chang, H. C. W., & Chang, L. R. (2012). Self-reported psychopathology and health-related quality of life in heroin users treated with methadone.*9*, 41-48.

154. Chin, W. Y., Chan, K. T. Y., Lam, C. L. K., Wan, E. Y. F., & Lam, T. P. (2015). 12-month naturalistic outcomes of depressive disorders in hong kong's primary care.*32*, 288-296.

155. Choi, J., Choi, K., Reddy, L., & Fiszdon, J. M. (2014). Measuring motivation in schizophrenia: Is a general state of motivation necessary for task-specific motivation?*153*, 209-213.

156. Chopra, M. P., Zhang, H., Kaiser, A. P., Moye, J. A., Llorente, M. D., Oslin, D. W., & Spiro, I. A. (2014). PTSD is a chronic, fluctuating disorder affecting the mental quality of life in older adults.*22*, 86-97.

157. Chou, C. Y., Ma, M. C., & Yang, T. T. (2014). Determinants of subjective health-related quality of life (HRQoL) for patients with schizophrenia.*154*, 83-88.

158. Chou, C. Y., Yang, T. T., Ma, M. C., Teng, P. R., & Cheng, T. C. (2015). Psychometric validations and comparisons of schizophrenia-specific health-related quality of life measures.*226*, 257-263.

159. Chou, K. R., Shih, Y. W., Chang, C., Chou, Y. Y., Hu, W. H., Cheng, J. S., . . . Hsieh, C. J. (2012). Psychosocial rehabilitation activities, empowerment, and quality of community-based life for people with schizophrenia.*26*, 285-294.

160. Christl, B., Reilly, N., Yin, C., & Austin, M. (2015). Clinical profile and outcomes of women admitted to a psychiatric mother-baby unit.*18*, 805-816.

161. Chronister, J., Chou, C., & Liao, H. (2013). The role of stigma coping and social support in mediating the effect of societal stigma on internalized stigma, mental health recovery, and quality of life among people with serious mental illness.*41*, 582-600.

162. Chugh, P. K., Rehan, H. S., Unni, K. E. S., & Sah, R. K. (2013). Predictive value of symptoms for quality of life in first-episode schizophrenia.*67*, 153-158.

163. Chum, A., Skosireva, A., Tobon, J., & Hwang, S. (2016). Construct validity of the SF-12v2 for the homeless population with mental illness: An instrument to measure self-reported mental and physical health.*11*

164. Chung, K. F., Tso, K. C., Yeung, W. F., & Li, W. H. (2012). Quality of life in major depressive disorder: The role of pain and pain catastrophizing cognition.*53*, 387-395.

165. Cicek, E., Cicek, I. E., Kayhan, F., Uguz, F., & Kaya, N. (2013). Quality of life, family burden and associated factors in relatives with obsessive-compulsive disorder.*35*, 253-258.

166. Cichocki, Å., Cechnicki, A., Franczyk-Glita, J., BÅ‚Ä…dziÅ„ski, P., Kalisz, A., & WroÅ„ski, K. (2015). Quality of life in a 20-year follow-up study of people suffering from schizophrenia.*56*, 133-140.

167. Ciketic, S., McKetin, R., Doran, C. M., Najman, J. M., Veerman, J. L., & Hayatbakhsh, R. M. (2013). Health-related quality of life (HRQL) among methamphetamine users in treatment.*6*, 250-261.

168. Clausen, H., Landheim, A., Odden, S., Heiervang, K. S., Stuen, H. K., Killaspy, H., . . . Ruud, T. (2015). Associations between quality of life and functioning in an assertive community treatment population.*66*, 1249-1252.

169. Clayton, A., O'Connell, M. J., Bellamy, C., Benedict, P., & Rowe, M. (2013). The citizenship project part II: Impact of a citizenship intervention on clinical and community outcomes for persons with mental illness and criminal justice involvement.*51*, 114-122.

170. Cohen, A. S., Auster, T. L., MacAulay, R. K., & McGovern, J. E. (2014). The paradox of schizotypy: Resemblance to prolonged severe mental illness in subjective but not objective quality of life.*217*, 185-190.

171. Cohen, C. I., Vengassery, A., & Garcia Aracena, E. F. (2017). A longitudinal analysis of quality of life and associated factors in older adults with schizophrenia spectrum disorder.*25*, 755-765.

172. Cohen, R. M., Greenberg, J. M., & IsHak, W. W. (2013). Incorporating multidimensional patient-reported outcomes of symptom severity, functioning, and quality of life in the individual burden of illness index for depression to measure treatment impact and recovery in MDD.*70*, 343-350.

173. Coker, E. L., Mitchell-Wong, L. A., & Abraham, S. F. (2013). Is pregnancy a trigger for recovery from an eating disorder?*92*, 1407-1413.

174. Coleman, J. A., Harper, L. A., Perrin, P. B., Landa, L. O., Olivera, S. L., Perdomo, J. L., . . . Arango-Lasprilla, J. C. (2015). Mind and body: Mental health and health related quality of life in SCI caregivers from neiva, colombia.*36*, 223-232.

175. Colpaert, K., De Maeyer, J., Broekaert, E., & Vanderplasschen, W. (2013). Impact of addiction severity and psychiatric comorbidity on the quality of life of alcohol-, drug-and dual-dependent persons in residential treatment.*19*, 173-183.

176. Cook, B., Engel, S., Crosby, R., Hausenblas, H., Wonderlich, S., & Mitchell, J. (2014). Pathological motivations for exercise and eating disorder specific health-related quality of life.*47*, 268-272.

177. Cortesi, P. A., Mencacci, C., Luigi, F., Pirfo, E., Berto, P., Sturkenboom, M. C. J. M., . . . Scalone, L. (2013). Compliance, persistence, costs and quality of life in young patients treated with antipsychotic drugs: Results from the COMETA study.*13*

178. Costa, R. T., Cheniaux, E., Rang√©, B. P., Versiani, M., & Nardi, A. E. (2012). Group cognitive behavior therapy for bipolar disorder can improve the quality of life.*45*, 862-868.

179. Cotrena, C., Branco, L. D., Kochhann, R., Shansis, F. M., & Fonseca, R. P. (2016). Quality of life, functioning and cognition in bipolar disorder and major depression: A latent profile analysis.*241*, 289-296.

180. Cotrena, C., Branco, L. D., Shansis, F. M., & Fonseca, R. P. (2016). Executive function impairments in depression and bipolar disorder: Association with functional impairment and quality of life.*190*, 744-753.

181. Cougle, J. R., Hakes, J. K., Macatee, R. J., Chavarria, J., & Zvolensky, M. J. (2015). Quality of life and risk of psychiatric disorders among regular users of alcohol, nicotine, and cannabis: An analysis of the national epidemiological survey on alcohol and related conditions (NESARC).*66-67*, 135-141.

182. Crempien, C., Grez, M., ValdÃ©s, C., LÃ□pez, M. J., de la Parra, G., & Krause, M. (2017). Role of personality functioning in the quality of life of patients with depression.

183.Crisp, D., Griffiths, K., Mackinnon, A., Bennett, K., & Christensen, H. (2014). An online intervention for reducing depressive symptoms: Secondary benefits for self-esteem, empowerment and quality of life.*216*, 60-66.

184.Cruz, B. F., de Resende, C. B., Carvalhaes, C. F., Cardoso, C. S., Teixeira, A. L., Keefe, R. S., . . . Salgado, J. V. (2016). Interview-based assessment of cognition is a strong predictor of quality of life in patients with schizophrenia and severe negative symptoms.*38*, 216-221.

185.Cruz, D. L., Lai, Z., Goodrich, D. E., & Kilbourne, A. M. (2013). Gender differences in health-related quality of life in patients with bipolar disorder.*16*, 317-323.

186.Cruz-Feliciano, M. A., Miranda-DÃaz, C., FernÃ¡ndez-Santos, D. M., Orobitg-Brenes, D., Hunter-Mellado, R. F., & CarriÃ□n-GonzÃ¡lez, I. S. (2017). Quality of life improvement in latinas receiving combined substance use disorders and trauma-specific treatment: A cohort evaluation report.*15*

187.Cudney, L. E., Frey, B. N., Streiner, D. L., Minuzzi, L., & Sassi, R. B. (2016). Biological rhythms are independently associated with quality of life in bipolar disorder.*4*

188.Cullen, B. A., La Flair, L. N., Storr, C. L., Green, K. M., Alvanzo, A. A. H., Mojtabai, R., . . . Crum, R. M. (2013). Association of comorbid generalized anxiety disorder and alcohol use disorder symptoms with health-related quality of life: Results from the national epidemiological survey on alcohol and related conditions.*7*, 394-400.

189.Currier, J. M., Drescher, K. D., Holland, J. M., Lisman, R., & Foy, D. W. (2016). Spirituality, forgiveness, and quality of life: Testing a mediational model with military veterans with PTSD.*26*, 167-179.

190.Cyr, K. S., McIntyre-Smith, A., Contractor, A. A., Elhai, J. D., & Richardson, J. D. (2014). Somatic symptoms and health-related quality of life among treatment-seeking canadian forces personnel with PTSD.*218*, 148-152.

191.Da Costa, R. T., De Carvalho, M. R., Cantini, J., Da Rocha Freire, R. C., & Nardi, A. E. (2014). Demographics, clinical characteristics and quality of life of brazilian women with driving phobia.*55*, 374-379.

192.Da Silva, J. P., & Pereira, A. M. (2017). Perceived spirituality, mindfulness and quality of life in psychiatric patients.*56*, 130-140.

193. Daeppen, J. B., Faouzi, M., Sanchez, N., Rahhali, N., Bineau, S., & Bertholet, N. (2014). Quality of life depends on the drinking pattern in alcohol-dependent patients.*49*, 457-465.

194. Daigre, C., Grau-LÃ□pez, L., RodrÃguez-Cintas, L., Ros-Cucurull, E., Sorribes-Puertas, M., Esculies, O., . . . Roncero, C. (2017). The role of dual diagnosis in health-related quality of life among treatment-seeking patients in spain., 1-9.

195. Dalky, H. F., Qandil, A. M., Natour, A. S., & Janet, M. C. (2017). Quality of life, stigma and burden perception among family caregivers and patients with psychiatric illnesses in jordan.*53*, 266-274.

196. Danovitch, I., Steiner, A. J., Kazdan, A., Goldenberg, M., Haglund, M., Mirocha, J., . . . Ishak, W. W. (2017). Analysis of patient-reported outcomes of quality of life and functioning before and after treatment of major depressive disorder comorbid with alcohol use disorders.*11*, 47-54.

197. Datto, C., SvedsÃ□ter, H., Locklear, J. C., & Endicott, J. (2013). Effect of extended-release quetiapine fumarate on quality of life and sleep in elderly patients with generalized anxiety disorder.*3*, 577-585.

198. De Abreu, L. N., Nery, F. G., Harkavy-Friedman, J. M., De Almeida, K. M., Gomes, B. C., Oquendo, M. A., & Lafer, B. (2012). Suicide attempts are associated with worse quality of life in patients with bipolar disorder type i.*53*, 125-129.

199. de Almeida, J. G., Braga, P. E., Neto, F. L., & Pimenta, C. A. M. (2013). Chronic pain and quality of life in schizophrenic patients.*35*, 13-20.

200. De AraÃºjo, A. A., De AraÃºjo Dantas, D., Do Nascimento, G. G., Ribeiro, S. B., Chaves, K. M., De Lima Silva, V., . . . De Medeiros, C. A. C. X. (2014). Quality of life in patients with schizophrenia: The impact of socio-economic factors and adverse effects of atypical antipsychotics drugs.*85*, 357-367.

201. de AraÃºjo, A. A., RebouÃ§as Barbosa, R. A. S., de Menezes, M. S. S., de Medeiros, I. I. F., de AraÃºjo, R. F., & de Medeiros, C. A. C. X. (2016). Quality of life, family support, and comorbidities in institutionalized elders with and without symptoms of depression.*87*, 281-291.

202. De Barros Pellegrinelli, K., de, O. C. L. F., Silval, K. I. D., Dias, V. V., Roso, M. C., Bandeira, M., . . . Moreno, R. A. (2013). Efficacy of psychoeducation on symptomatic and functional recovery in bipolar disorder.*127*, 153-158.

203.de Haan, L., Sterk, B., & van der Valk, R. (2013). Presence of obsessive compulsive symptoms in first-episode schizophrenia or related disorders is associated with subjective well-being and quality of life.*7*, 285-290.

204.de Lima Silva, V., de Medeiros, C. A. C. X., Guerra, G. C. B., Ferreira, P. H. A., de AraÃºjo JÃºnior, R. F., de AraÃºjo Barbosa, S. J., & de AraÃºjo, A. A. (2017). Quality of life, integrative community therapy, family support, and satisfaction with health services among elderly adults with and without symptoms of depression.*88*, 359-369.

205.De Maeyer, J., van Nieuwenhuizen, C., Bongers, I. L., Broekaert, E., & Vanderplasschen, W. (2013). Profiles of quality of life in opiate-dependent individuals after starting methadone treatment: A latent class analysis.*24*, 342-350.

206.Deckersbach, T., Nierenberg, A. A., McInnis, M. G., Salcedo, S., Bernstein, E. E., Kemp, D. E., . . . Kamali, M. (2016). Baseline disability and poor functioning in bipolar disorder predict worse outcomes: Results from the bipolar CHOICE study.*77*, 100-108.

207.Deenik, J., Kruisdijk, F., Tenback, D., Braakman-Jansen, A., Taal, E., Hopman-Rock, M., . . . van Harten, P. (2017). Physical activity and quality of life in long-term hospitalized patients with severe mental illness: A cross-sectional study.*17*

208.Demant, K. M., Vinberg, M., Kessing, L. V., & Miskowiak, K. W. (2015). Effects of short-term cognitive remediation on cognitive dysfunction in partially or fully remitted individuals with bipolar disorder: Results of a randomised controlled trial.*10*

209.Demir, M. O., YÄ±ldÄ±z, M., Batmaz, S., Semiz, M., Songur, E., Ã‡akmak, S., & Demir, O. (2016). Expressed emotion in panic disorder: Relationship with demographic and clinical variables and quality of life.*62*, 394-399.

210.Deng, H., Wang, J., Zhang, X., Ma, M., Domingo, C., Sun, H., & Kosten, T. (2016). International perspective: Smoking reduction and quality of life in chronic patients with schizophrenia in a chinese population-a pilot study.*25*, 86-90.

211.Derks, E., Cahn, W., Kahn, R. S., Linszen, D. H., Van Os, J., Wiersma, D., . . . Fett, A. K. (2012). Social cognition and quality of life in schizophrenia.*137*, 212-218.

212. Detweiler, M. B., Self, J. A., Lane, S., Spencer, L., Lutgens, B., Kim, D. Y., . . . Lehmann, L. P. (2015). Horticultural therapy: A pilot study on modulating cortisol levels and indices of substance craving, posttraumatic stress disorder, depression, and quality of life in veterans.*21*, 36-41.

213. DiÅŸsiz, M., Beji, N., & Oskay, Ã. (2015). The effects of alcohol dependence on the quality of life and sex life of women.*50*, 1373-1382.

214. Diefenbach, G. J., Wootton, B. M., Bragdon, L. B., Moshier, S. J., & Tolin, D. F. (2015). Treatment outcome and predictors of internet guided self-help for obsessive-compulsive disorder.*46*, 764-774.

215. Dijkhuis, R. R., Ziermans, T. B., Van Rijn, S., Staal, W. G., & Swaab, H. (2017). Self-regulation and quality of life in high-functioning young adults with autism.*21*, 896-906.

216. Dingemans, A. E., Danner, U. N., Donker, J. M., Aardoom, J. J., van Meer, F., Tobias, K., . . . van Furth, E. F. (2014). The effectiveness of cognitive remediation therapy in patients with a severe or enduring eating disorder: A randomized controlled trial.*83*, 29-36.

217. DomÃnguez-MartÃnez, T., Kwapil, T. R., & Barrantes-Vidal, N. (2015). Subjective quality of life in at-risk mental state for psychosis patients: Relationship with symptom severity and functional impairment.*9*, 292-299.

218. Donald, S., Chartrand, H., & Bolton, J. M. (2013). The relationship between nicotine cessation and mental disorders in a nationally representative sample.*47*, 1673-1679.

219. Dryman, M. T., Gardner, S., Weeks, J. W., & Heimberg, R. G. (2016). Social anxiety disorder and quality of life: How fears of negative and positive evaluation relate to specific domains of life satisfaction.*38*, 1-8.

220. Dumas, R., Richieri, R., Guedj, E., Auquier, P., Lancon, C., & Boyer, L. (2012). Improvement of health-related quality of life in depression after transcranial magnetic stimulation in a naturalistic trial is associated with decreased perfusion in precuneus.*10*

221. Egede, L. E., Acierno, R., Knapp, R. G., Walker, R. J., Payne, E. H., & Christopher Frueh, B. (2016). Psychotherapy for depression in older veterans via telemedicine: Effect on quality of life, satisfaction, treatment credibility, and service delivery perception.*77*, 1704-1711.

222. Eklund, M., Gunnarsson, A. B., Sandlund, M., & Leufstadius, C. (2014). Effectiveness of an intervention to improve day centre services for people with psychiatric disabilities.*61*, 268-275.

223.Eklund, M., & Sandlund, M. (2012). The life situation of people with persistent mental illness visiting day centers: A comparative study.*48*, 592-597.

224.El Alaoui, S., Hedman, E., Ljotsson, B., & Lindefors, N. (2015). Long-term effectiveness and outcome predictors of therapist-guided internet-based cognitive-behavioural therapy for social anxiety disorder in routine psychiatric care.*5*, e007902.

225.Elisabeth, A., Carina, T., & Mona, E. (2017). Quality of life among people with psychiatric disabilities: Does day centre attendance make a difference?

226.Endicott, J., Lam, R. W., Hsu, M. A., Fayyad, R., Boucher, M., & Guico-Pabia, C. J. (2014). Improvements in quality of life with desvenlafaxine 50 mg/d vs placebo in employed adults with major depressive disorder.*166*, 307-314.

227.Engel, C. C., Jaycox, L. H., Freed, M. C., Bray, R. M., Brambilla, D., Zatzick, D., . . . Katon, W. J. (2016). Centrally assisted collaborative telecare for posttraumatic stress disorder and depression among military personnel attending primary care: A randomized clinical trial.*176*, 948-956.

228.Engel-Yeger, B., Gonda, X., Muzio, C., Rinosi, G., Pompili, M., Amore, M., & Serafini, G. (2016). Sensory processing patterns, coping strategies, and quality of life among patients with unipolar and bipolar disorders.*38*, 207-215.

229.Ertekin, H., Er, M., Yardim Ozayhan, H., Yayla, S., Akyol, E., & Sahin, B. (2015). Quality of life and depression in schizophrenia patients living in a nursing home.*28*, 213-221.

230.Erten, E., Funda Uney, A., SaatÃ§ioÇ§lu, Ã., Ã–zdemir, A., FistikÃ§i, N., & Ã‡akmak, D. (2014). Effects of childhood trauma and clinical features on determining quality of life in patients with bipolar i disorder.*162*, 107-113.

231.Eshagh Afkari, M., Ghasemi, A., Shojaeizadeh, D., Tol, A., Rahimi Foroshani, A., & Taghdisi, M. H. (2013). Comparison between family function dimensions and quality of life among amphetamine addicts and non- addicts.*15*, 356-362.

232.Eustis, E. H., Hayes-Skelton, S. A., Roemer, L., & Orsillo, S. M. (2016). Reductions in experiential avoidance as a mediator of change in symptom outcome and quality of life in acceptance-based behavior therapy and applied relaxation for generalized anxiety disorder.*87*, 188-195.

233. Evensen, J., Rossberg, J. I., Barder, H., Haahr, U., ten Velden Hegelstad, W., Joa, I., . . . McGlashan, T. (2012). Apathy in first episode psychosis patients: A ten year longitudinal follow-up study.*136*, 19-24.
234. Faget-Agius, C., Boyer, L., Richieri, R., Auquier, P., LanÃ§on, C., & Guedj, E. (2016). Functional brain substrate of quality of life in patients with schizophrenia: A brain SPECT multidimensional analysis.*249*, 67-75.
235. Faget-Agius, C., Catherine, F. A., Boyer, L., Wirsich, J., Jonathan, W., Ranjeva, J. P., . . . Christophe, L. (2015). Neural substrate of quality of life in patients with schizophrenia: A magnetisation transfer imaging study.*5*, 17650.
236. Fan, L., Fu, W., Chen, Z., Xu, N., Liu, J., LÃ□, A., . . . Ou, A. (2016). Curative effect of acupuncture on quality of life in patient with depression: A clinical randomized single-blind placebo-controlled study.*36*, 151-159.
237. Fang, S. C., Schnurr, P. P., Kulish, A. L., Holowka, D. W., Marx, B. P., Keane, T. M., & Rosen, R. (2015). Psychosocial functioning and health-related quality of life associated with posttraumatic stress disorder in male and female iraq and afghanistan war veterans: The VALOR registry.*24*, 1038-1046.
238. Farholm, A., Sorensen, M., & Halvari, H. (2016). Motivational factors associated with physical activity and quality of life in people with severe mental illness.
239. Faridhosseini, F., Baniasadi, M., Fayyazi Bordbar, M. R., Pourgholami, M., Ahrari, S., & Asgharipour, N. (2017). Effectiveness of psychoeducational group training on quality of life and recurrence of patients with bipolar disorder.*12*, 21-28.
240. Faries, D., Ascher-Svanum, H., Phillips, G., Nyhuis, A. W., Sugihara, T., Stauffer, V., & Kinon, B. J. (2012). Construct validity of 2 measures to assess reasons for antipsychotic discontinuation and continuation from patients' and clinicians' perspectives in a clinical trial.*12*, 142.
241. Farrer, L., Christensen, H., Griffiths, K. M., & Mackinnon, A. (2012). Web-based cognitive behavior therapy for depression with and without telephone tracking in a national helpline: Secondary outcomes from a randomized controlled trial.*14*, 64-73.
242. Fatehi, F., Monajemi, A., Sadeghi, A., Mojtahedzadeh, R., & Mirzazadeh, A. (2016). Quality of life in medical students with internet addiction.*54*, 663-667.

243.Fattori, A., Neri, L., Bellomo, A., Vaggi, M., & Mencacci, C. (2017). Depression severity and concentration difficulties are independently associated with HRQOL in patients with unipolar depressive disorders.*26*, 2459-2469.

244.Faugere, M., Micoulaud-Franchi, J. A., Alessandrini, M., Richieri, R., Faget-Agius, C., Auquier, P., . . . Boyer, L. (2015). Quality of life is associated with chronic inflammation in schizophrenia: A cross-sectional study.*5*, 10793.

245.Fei, J. T. B., Yee, A., & Habil, M. H. B. (2016). Psychiatric comorbidity among patients on methadone maintenance therapy and its influence on quality of life.*25*, 49-55.

246.Ferre, F., Cambra, J., Ovejero, M., & Basurte-Villamor, I. (2017). Influence of attention deficit hyperactivity disorder symptoms on quality of life and functionality in adults with eating disorders.*45*, 98-107.

247.Fervaha, G., Foussias, G., Agid, O., & Remington, G. (2014). Motivational and neurocognitive deficits are central to the prediction of longitudinal functional outcome in schizophrenia.*130*, 290-299.

248.Fervaha, G., Foussias, G., Siddiqui, I., Agid, O., & Remington, G. (2014). Abbreviated quality of life scales for schizophrenia: Comparison and utility of two brief community functioning measures.*154*, 89-92.

249.Fervaha, G., Foussias, G., Takeuchi, H., Agid, O., & Remington, G. (2016). Motivational deficits in major depressive disorder: Cross-sectional and longitudinal relationships with functional impairment and subjective well-being.*66*, 31-38.

250.Fervaha, G., & Remington, G. (2013). Validation of an abbreviated quality of life scale for schizophrenia.*23*, 1072-1077.

251.Fervaha, G., Agid, O., Takeuchi, H., Foussias, G., & Remington, G. (2013). Clinical determinants of life satisfaction in chronic schizophrenia: Data from the CATIE study.*151*, 203-208.

252.FilipÄ□iÄ‡, I., Å imunoviÄ‡ FilipÄ□iÄ‡, I., MatiÄ‡, K., LovretiÄ‡, V., IveziÄ‡, E., BajiÄ‡, Z., . . . VÄ□ev, A. (2016). Somatic comorbidities are independently associated with the poor health-related quality of life in psychiatric patients.*28*, 284-292.

253.Fleury, M. J., Grenier, G., & Bamvita, J. M. (2015). Predictive typology of subjective quality of life among participants with severe mental disorders after a five-year follow-up: A longitudinal two-step cluster analysis.*13*

254.Fleury, M. J., Grenier, G., Bamvita, J. M., Tremblay, J., Schmitz, N., & Caron, J. (2013). Predictors of quality of life in a longitudinal study of users with severe mental disorders.*11*

255.Florea, I., Danchenko, N., Brignone, M., Loft, H., Rive, B., & Abetz-Webb, L. (2015). The effect of vortioxetine on health-related quality of life in patients with major depressive disorder.*37*, 2309-2323.e6.

256.Foldemo, A., WÃ□rdig, R., Bachrach-LindstrÃ¶m, M., Edman, G., Holmberg, T., LindstrÃ¶m, T., . . . Ã–sby, U. (2014). Health-related quality of life and metabolic risk in patients with psychosis.*152*, 295-299.

257.Fontanil-Gomez, Y., Alcedo Rodriguez, M. A., & Gutierrez Lopez, M. I. (2017). Personal and macro-systemic factors as predictors of quality of life in chronic schizophrenia.*29*, 160-165.

258.Fowler, J. C., Madan, A., Allen, J. G., Ellis, T., Mahoney, J., Hardesty, S., . . . Oldham, J. M. (2015). Improvement in health-related quality of life among adults with serious mental illness receiving inpatient treatment: A prospective cohort study.*76*, e632-e638.

259.FrÃas, Ã., Palma, C., Farriols, N., Salvador, A., Bonet, J., & BernÃ¡ldez, I. (2014). Psychopathology and quality of life among patients with comorbidity between schizophrenia spectrum disorder and obsessive-compulsive disorder: No evidence for a "schizo-obsessive" subtype.*55*, 1165-1173.

260.FranÃ§ois, C., Rahhali, N., Chalem, Y., SÃ¸rensen, P., Luquiens, A., & Aubin, H. J. (2015). The effects of as-needed nalmefene on patient-reported outcomes and quality of life in relation to a reduction in alcohol consumption in alcohol-dependent patients.*10*

261.Franz, M., Fritz, M., Gallhofer, B., & Meyer, T. (2012). QLiS--development of a schizophrenia-specific quality-of-life scale.*10*, 61.

262.Franz, M., Fritz, M., & Meyer, T. (2013). Discriminant and convergent validity of a subjective quality-of-life instrument aimed at high content validity for schizophrenic persons.*22*, 1113-1122.

263.Frischknecht, U., Sabo, T., & Mann, K. (2013). Improved drinking behaviour improves quality of life: A follow-up in alcohol-dependent subjects 7 years after treatment.*48*, 579-584.

264.Fu, C. K. J., Chow, P. L. P., Lam, W. S. J., Tung, C. K., & Cheung, Y. L. F. (2013). Validation of the chinese version of perception of care in an acute psychiatric ward in hong kong.*5*, 322-330.

265.Fujimaki, K., Morinobu, S., Yamashita, H., Takahashi, T., & Yamawaki, S. (2012). Predictors of quality of life in inpatients with schizophrenia.*197*, 199-205.

266.Fujimaki, K., Takahashi, T., & Morinobu, S. (2012). Association of typical versus atypical antipsychotics with symptoms and quality of life in schizophrenia.*7*

267.Fujino, H., Sumiyoshi, C., Sumiyoshi, T., Yasuda, Y., Yamamori, H., Ohi, K., . . . Imura, O. (2016). Predicting employment status and subjective quality of life in patients with schizophrenia.*3*, 20-25.

268.Fulford, D., Peckham, A. D., Johnson, K., & Johnson, S. L. (2014). Emotion perception and quality of life in bipolar i disorder.*152-154*, 491-497.

269.Furrer, M., Jakob, N. J., Cattapan-Ludewing, K., Seixas, A., Huber, C. G., & Schneeberger, A. R. (2017). Patient satisfaction and quality of life in people with schizophrenia-spectrum disorders in a rural area.

270.GÃ¡lvez, V., Li, A., Oxley, C., Waite, S., De Felice, N., Hadzi-Pavlovic, D., . . . Loo, C. K. (2016). Health related quality of life after ECT for depression: A study exploring the role of different electrode-placements and pulse-widths.*206*, 268-272.

271.GÃ□mez-de-Regil, L. (2016). Psychometric properties of the seville quality of life questionnaire in mexican patients with psychosis.*16*

272.Gal, E., Selanikyo, E., Erez, A. B. H., & Katz, N. (2015). Integration in the vocational world:How does it affect quality of life and subjective well-being of young adults with ASD.*12*, 10820-10832.

273.Gao, K., Sweet, J., Su, M., & Calabrese, J. R. (2017). Depression severity and quality of life of qualified and unqualified patients with a mood disorder for a research study targeting anhedonia in a clinical sample.*27*, 40-47.

274.Gardsjord, E. S., Romm, K. L., Friis, S., Barder, H. E., Evensen, J., Haahr, U., . . . RÃ¸ssberg, J. I. (2016). Subjective quality of life in first-episode psychosis. A ten year follow-up study.*172*, 23-28.

275.Gardsjord, E. S., Romm, K. L., RÃ¸ssberg, J. I., Friis, S., Barder, H. E., Evensen, J., . . . Melle, I. (2017). Is going into stable symptomatic remission associated with a more positive development of life satisfaction? A 10-year follow-up study of first episode psychosis.

276.Garrido, G., Barrios, M., PenadÃ©s, R., EnrÃquez, M., Garolera, M., Aragay, N., . . . Vendrell, J. M. (2013). Computer-assisted cognitive remediation therapy: Cognition, self-esteem and quality of life in schizophrenia.*150*, 563-569.

277.Gassmann, W., Christ, O., Lampert, J., & Berger, H. (2013). The influence of antonovsky's sense of coherence (SOC) and psychoeducational family intervention (PEFI) on schizophrenic outpatients' perceived quality of life: A longitudinal field study.*13*

278.Gattaz, W. F., De Oliveira Campos, J. A., Lacerda, A. L. T., Henna, E., Ruschel, S. I., Bressan, R. A., . . . AppolinÃ¡rio, J. C. (2014). Switching from oral risperidone to flexibly dosed oral paliperidone extended-release: Core symptoms, satisfaction, and quality of life in patients with stable but symptomatic schizophrenia: The RISPALI study.*30*, 695-709.

279.Gauhar, Y. W. M. (2016). The efficacy of EMDR in the treatment of depression.*10*, 59-69.

280.Gelkopf, M., Pagorek-Eshel, S., Trauer, T., & Roe, D. (2015). Routine outcome measurement in mental health service consumers: Who should provide support for the self-assessments?*50*, 43-46.

281.Gerritsen, C. J., Goldberg, J. O., & Eastwood, J. D. (2015). Boredom proneness predicts quality of life in outpatients diagnosed with schizophrenia-spectrum disorders.*61*, 781-787.

282.Ghasemi, A., Rahimi Foroshani, A., Kheibar, N., Latifi, M., Khanjani, N., Eshagh Afkari, M., . . . Dastoorpour, M. (2014). Effects of family-centered empowerment model based education program on quality of life in methamphetamine users and their families.*16*

283.Giacco, D., Matanov, A., & Priebe, S. (2013). Symptoms and subjective quality of life in post-traumatic stress disorder: A longitudinal study.*8*

284.Giacco, D., McCabe, R., Kallert, T., Hansson, L., Fiorillo, A., & Priebe, S. (2012). Friends and symptom dimensions in patients with psychosis: A pooled analysis.*7*

285.Giesen, E. S., Zimmer, P., & Bloch, W. (2016). Effects of an exercise program on physical activity level and quality of life in patients with severe alcohol dependence.*34*, 63-78.

286.Gigaux, J., Le Gall, D., Jollant, F., Lhuillier, J. P., & Richard-Devantoy, S. (2013). Cognitive inhibition and quality of life in schizophrenia: A pilot study.*143*, 297-300.

287.Gili, M., MagallÃ□n, R., LÃ□pez-Navarro, E., Roca, M., Moreno, S., BauzÃ¡, N., & GarcÃa-Cammpayo, J. (2014). Health related quality of life changes in somatising patients after individual versus group cognitive behavioural therapy: A randomized clinical trial.*76*, 89-93.

288.Gill, J. M., Saligan, L., Lee, H., Rotolo, S., & Szanton, S. (2013). Women in recovery from PTSD have similar inflammation and quality of life as non-traumatized controls.*74*, 301-306.

289.GimÃ©nez-Meseguer, J., Tortosa-MartÃnez, J., & Remedios FernÃ¡ndez-Valenciano, M. D. L. (2015). Benefits of exercise for the quality of life of drug-dependent patients.*47*, 409-416.

290.Girard, V., Tinland, A., Bonin, J. P., Olive, F., Poule, J., Lancon, C., . . . Simeoni, M. C. (2017). Relevance of a subjective quality of life questionnaire for long-term homeless persons with schizophrenia.*17*

291.Girard, V., Tinland, A., Boucekine, M., LoubiÃ¨re, S., Lancon, C., Boyer, L., & Auquier, P. (2016). Validity of a common quality of life measurement in homeless individuals with bipolar disorder and schizophrenia.*204*, 131-137.

292.Giri, O. P., Srivastava, M., & Shankar, R. (2014). Quality of life and health of opioid-dependent subjects in india.*5*, 363-368.

293.Gjervan, B., Torgersen, T., Rasmussen, K., & Nordahl, H. M. (2014). ADHD symptoms are differentially related to specific aspects of quality of life.*18*, 598-606.

294.Gold, P. B. (2013). Quality of life and competitive work among adults with severe mental illness: Moderating effects of family contact.*64*, 1218-1224.

295.Gold, P. B., Macias, C., & Rodican, C. F. (2016). Does competitive work improve quality of life for adults with severe mental illness? evidence from a randomized trial of supported employment.*43*, 155-171.

296.Gomes, E., Bastos, T., Probst, M., Ribeiro, J. C., Silva, G., & Corredeira, R. (2014). Effects of a group physical activity program on physical fitness and quality of life in individuals with schizophrenia.*7*, 155-162.

297.Gomes, E., Bastos, T., Probst, M., Ribeiro, J. C., Silva, G., & Corredeira, R. (2016). Quality of life and physical activity levels in outpatients with schizophrenia.*38*, 157-160.

298.Gomez-de-Regil, L. (2015). Illness course and quality of life in mexican patients with psychosis.*8*, 218-223.

299.Gomez-de-Regil, L., Kwapil, T. R., & Barrantes-Vidal, N. (2014). Illness perception mediates the effect of illness course on the quality of life of mexican patients with psychosis.*9*, 99-112.

300.Goodson, J. T., Lefkowitz, C. M., Helstrom, A. W., & Gawrysiak, M. J. (2013). Outcomes of prolonged exposure therapy for veterans with posttraumatic stress disorder.*26*, 419-425.

301. Goorden, M., Huijbregts, K. M., van Marwijk, H. W., Beekman, A. T., van der Feltz-Cornelis, Christina M., & Hakkaart-van Roijen. (2015). Cost-utility of collaborative care for major depressive disorder in primary care in the netherlands.*79*, 316-323.

302. Gordon, J. S., Staples, J. K., He, D. Y., & Atti, J. A. A. (2016). Mind-body skills groups for posttraumatic stress disorder in palestinian adults in gaza.*22*, 155-164.

303. Gorin-Lazard, A., Baumstarck, K., Boyer, L., Maquigneau, A., Penochet, J. C., Pringuey, D., . . . Auquier, P. (2013). Hormonal therapy is associated with better self-esteem, mood, and quality of life in transsexuals.*201*, 996-1000.

304. GrÃ□nder, G., Heinze, M., Cordes, J., MÃ□hlbauer, B., Juckel, G., Schulz, C., . . . Timm, J. (2016). Effects of first-generation antipsychotics versus second-generation antipsychotics on quality of life in schizophrenia: A double-blind, randomised study.*3*, 717-729.

305. Grambal, A., Prasko, J., Kamaradova, D., Latalova, K., Holubova, M., SedlÃ¡Ä□kovÃ¡, Z., & Hruby, R. (2016). Quality of life in borderline patients comorbid with anxiety spectrum disorders - A cross-sectional study.*10*, 1421-1433.

306. Greenberg, T., Bertocci, M. A., Chase, H. W., Stiffler, R., Aslam, H. A., Graur, S., . . . Phillips, M. L. (2017). Mediation by anxiety of the relationship between amygdala activity during emotion processing and poor quality of life in young adults.*7*, e1178.

307. Greer, T. L., Trombello, J. M., Rethorst, C. D., Carmody, T. J., Jha, M. K., Liao, A., . . . Trivedi, M. H. (2016). Improvements in psychosocial functioning and health-related quality of life following exercise augmentation in patients with treatment response but nonremitted major depressive disorder: Results from the tread study.*33*, 870-881.

308. Grendas, L., Rodante, D., Rojas, S., Puppo, S., Vidjen, P., Lado, G., . . . Daray, F. M. (2017). Determinants of mental and physical health-related quality of life among patients hospitalized for suicidal behavior.*257*, 56-60.

309. Griffin, M. L., Bennett, H. E., Fitzmaurice, G. M., Hill, K. P., Provost, S. E., & Weiss, R. D. (2015). Health-related quality of life among prescription opioid-dependent patients: Results from a multi-site study.*24*, 308-314.

310. Grochtdreis, T., Brettschneider, C., Schierz, K., Hoyer, J., & KÃ¶nig, H. H. (2016). Mapping the beck depression inventory to the EQ-5D-3L in patients with depressive disorders.*19*, 79-89.

311. Grocke, D., Bloch, S., Castle, D., Thompson, G., Newton, R., Stewart, S., & Gold, C. (2014). Group music therapy for severe mental illness: A randomized embedded-experimental mixed methods study.*130*, 144-153.

312. Guan, L., Xiang, Y., Ma, X., Weng, Y., & Liang, W. (2016). Qualities of life of patients with psychotic disorders and their family caregivers: Comparison between hospitalised and community-based treatment in beijing, china.*11*

313. Guilera, G., Gomez-Benito, J., Pino, O., Rojo, J. E., Cuesta, M. J., Martinez-Aran, A., . . . Rejas, J. (2012). Utility of the world health organization disability assessment schedule II in schizophrenia.*138*, 240-247.

314. Guligowska, A., PigÅ‚owska, M., Fife, E., Kostka, J., SoÅ‚tysik, B. K., Kroc, Å., & Kostka, T. (2016). Inappropriate nutrients intake is associated with lower functional status and inferior quality of life in older adults with depression.*11*, 1505-1517.

315. Guo, X., Zhang, Z., Zhai, J., Fang, M., Hu, M., Wu, R., . . . Zhao, J. (2012). Effects of antipsychotic medications on quality of life and psychosocial functioning in patients with early-stage schizophrenia: 1-year follow-up naturalistic study.*53*, 1006-1012.

316. Guo, X., Zhang, Z., Zhai, J., Wu, R., Liu, F., & Zhao, J. (2013). The relationship between obesity and health-related quality of life in chinese patients with schizophrenia.*17*, 16-20.

317. Gupta, K., & Mamidi, P. (2015). Efficacy of saraswata choorna on quality of life and manasika pariksha bhava's in generalized anxiety disorder: Ancillary findings.*6*, 216-220.

318. Hachul, H., Garcia, T. K. P., MacIel, A. L., Yagihara, F., Tufik, S., & Bittencourt, L. (2013). Acupuncture improves sleep in postmenopause in a randomized, double-blind, placebo-controlled study.*16*, 36-40.

319. Hacioglu Yildirim, M., Alantar, Z., & Yildirim, E. A. (2014). The relationship between working status and symptoms, quality of life and self-esteem in patients with schizophrenia in turkey.*60*, 646-655.

320. Hamaideh, S., Al-Magaireh, D., Abu-Farsakh, B., & Al-Omari, H. (2014). Quality of life, social support, and severity of psychiatric symptoms in jordanian patients with schizophrenia.*21*, 455-465.

321. Hamatani, S., Tomotake, M., Takeda, T., Kameoka, N., Kawabata, M., Kubo, H., . . . Ohmori, T. (2017). Influence of cognitive function on quality of life in anorexia nervosa patients.*71*, 328-335.

322. Happell, B., Stanton, R., Hodgetts, D., & Scott, D. (2016). Quality of life outcomes in community-based mental health consumers: Comparisons with population norms and changes over time.*37*, 146-152.

323. Harvey, C., Hawthorne, G., Favilla, A., Graham, C., & Herrman, H. (2012). Antipsychotic medicines in australian community practice: Effectiveness, adverse effects and quality of life for people with schizophrenia.*4*, 48-58.

324. Hasson-Ohayon, I., Avidan-Msika, M., Mashiach-Eizenberg, M., Kravetz, S., Rozencwaig, S., Shalev, H., & Lysaker, P. H. (2015). Metacognitive and social cognition approaches to understanding the impact of schizophrenia on social quality of life.*161*, 386-391.

325. Hasson-Ohayon, I., Mashiach-Eizenberg, M., Arnon-Ribenfeld, N., Kravetz, S., & Roe, D. (2017). Neuro-cognition and social cognition elements of social functioning and social quality of life.

326. Hawthorne, G., Korn, S., & Richardson, J. (2013). Population norms for the AQoL derived from the 2007 australian national survey of mental health and wellbeing.*37*, 7-16.

327. Hayhurst, K. P., Drake, R. J., Massie, J. A., Dunn, G., Barnes, T. R. E., Jones, P. B., & Lewis, S. W. (2014). Improved quality of life over one year is associated with improved adherence in patients with schizophrenia.*29*, 191-196.

328. Hayhurst, K. P., Massie, J. A., Dunn, G., Lewis, S. W., & Drake, R. J. (2014). Validity of subjective versus objective quality of life assessment in people with schizophrenia.*14*

329. He, Y., Li, N., Liu, D., & Zhao, L. (2016). Quality of life and negative moods of females enrolled in compulsory detoxification in china.*9*, 16981-16991.

330. Heidari, M., & Ghodusi, M. (2016). Relationship of assess self-esteem and locus of control with quality of life during treatment stages in patients referring to drug addiction rehabilitation centers.*28*, 263-267.

331. Helles, A., Gillberg, I. C., Gillberg, C., & Billstedt, E. (2017). Asperger syndrome in males over two decades: Quality of life in relation to diagnostic stability and psychiatric comorbidity.*21*, 458-469.

332. Helvik, A. S., BjÃ¸rklÃ¸f, G. H., Corazzini, K., SelbÃ□k, G., Laks, J., Ã˜stbye, T., & Engedal, K. (2016). Are coping strategies and locus of control orientation associated with health-related quality of life in older adults with and without depression?*64*, 130-137.

333.Helvik, A. S., Corazzini, K., SelbÃ□k, G., BjÃ¸rklÃ¸f, G. H., Laks, J., Å altytÄ— Benth, J., . . . Engedal, K. (2016). Health-related quality of life in older depressed psychogeriatric patients: One year follow-up.*16*, 131.

334.Henwood, B. F., Matejkowski, J., Stefancic, A., & Lukens, J. M. (2014). Quality of life after housing first for adults with serious mental illness who have experienced chronic homelessness.*220*, 549-555.

335.Herring, M. P., Johnson, K. E., & O'Connor, P. J. (2016). Exercise training and health-related quality of life in generalized anxiety disorder.*27*, 138-141.

336.Hertenstein, E., Thiel, N., Herbst, N., Freyer, T., Nissen, C., KÃ□lz, A. K., & Voderholzer, U. (2013). Quality of life changes following inpatient and outpatient treatment in obsessive-compulsive disorder: A study with 12 months follow-up.*12*

337.Hiranyatheb, T., Nakawiro, D., Wongpakaran, T., Wongpakaran, N., Bookkamana, P., Pinyopornpanish, M., . . . Tanchakvaranont, S. (2016). The impact of residual symptoms on relapse and quality of life among thai depressive patients.*12*, 3175-3181.

338.Hjorth, P., Medici, C. R., Juel, A., Madsen, N. J., Vandborg, K., & Munk-Jorgensen, P. (2017). Improving quality of life and physical health in patients with schizophrenia: A 30-month program carried out in a real-life setting.*63*, 287-296.

339.Hofer, A., Mizuno, Y., Wartelsteiner, F., Wolfgang Fleischhacker, W., Frajo-Apor, B., Kemmler, G., . . . Uchida, H. (2017). Quality of life in schizophrenia and bipolar disorder: The impact of symptomatic remission and resilience.*46*, 42-47.

340.Holliday, R., Williams, R., Bird, J., Mullen, K., & SurÃs, A. (2015). The role of cognitive processing therapy in improving psychosocial functioning, health, and quality of life in veterans with military sexual trauma-related posttraumatic stress disorder.*12*, 428-434.

341.Holubova, M., Prasko, J., Hruby, R., Kamaradova, D., Ociskova, M., Latalova, K., & Grambal, A. (2015). Coping strategies and quality of life in schizophrenia: Cross-sectional study.*11*, 3041-3048.

342.Holubova, M., Prasko, J., Latalova, K., Ociskova, M., Grambal, A., Kamaradova, D., . . . Hruby, R. (2016). Are self-stigma, quality of life, and clinical data interrelated in schizophrenia spectrum patients? A cross-sectional outpatient study.*10*, 265-274.

343. Holubova, M., Prasko, J., Matousek, S., Latalova, K., Marackova, M., Vrbova, K., . . . Zatkova, M. (2016). Comparison of self-stigma and quality of life in patients with depressive disorders and schizophrenia spectrum disorders - A cross-sectional study.*12*, 3021-3030.

344. Holubova, M., Prasko, J., Ociskova, M., Marackova, M., Grambal, A., & Slepecky, M. (2016). Self-stigma and quality of life in patients with depressive disorder: A cross-sectional study.*12*, 2677-2686.

345. Hong, J., Bishop-Fitzpatrick, L., Smith, L. E., Greenberg, J. S., & Mailick, M. R. (2016). Factors associated with subjective quality of life of adults with autism spectrum disorder: Self-report versus maternal reports.*46*, 1368-1378.

346. Hong, J., Novick, D., Montgomery, W., Moneta, M. V., DueÃ±as, H., Peng, X., & Haro, J. M. (2015). Health-related quality of life in patients with depression treated with duloxetine or a selective serotonin reuptake inhibitor in a naturalistic outpatient setting.*9*, 1481-1490.

347. Horn, E. K., Verheul, R., Thunnissen, M., Delimon, J., Goorden, M., Hakkaart-van Roijen, . . . Busschbach, J. J. (2016). Cost-effectiveness of short-term inpatient psychotherapy based on transactional analysis in patients with personality disorder.*30*, 483-501.

348. Horn, E. K., Verheul, R., Thunnissen, M., Delimon, J., Soons, M., Meerman, A. M., . . . Busschbach, J. J. (2015). Effectiveness of short-term inpatient psychotherapy based on transactional analysis with patients with personality disorders: A matched control study using propensity score.*29*, 663-683.

349. Hosakova, J., & Jarosova, D. (2015). Quality of life and needs of hospitalized schizophrenic patients in the czech republic.*36*, 288-293.

350. Hosseinzadeh Asl, N. R., & Hosseinalipour, F. (2014). Effectiveness of mindfulness-based stress reduction intervention for health-related quality of life in drug-dependent males.*16*

351. Hou, C. L., Cai, M. Y., Ma, X. R., Zang, Y., Jia, F. J., Lin, Y. Q., . . . Xiang, Y. T. (2015). Clozapine prescription and quality of life in chinese patients with schizophrenia treated in primary care.*48*, 200-204.

352. Hou, C. L., Li, Y., Cai, M. Y., Ma, X. R., Zang, Y., Jia, F. J., . . . Xiang, Y. T. (2017). Prevalence of insomnia and clinical and quality of life correlates in chinese patients with schizophrenia treated in primary care.*53*, 80-86.

353. Hou, C. L., Ma, X. R., Cai, M. Y., Li, Y., Zang, Y., Jia, F. J., . . . Xiang, Y. T. (2016). Comorbid moderate-severe depressive symptoms and their association with quality of life in chinese patients with schizophrenia treated in primary care.*52*, 921-926.

354. Hou, C. L., Ma, X. R., Zang, Y., Jia, F. J., Lin, Y. Q., Chiu, H. F. K., . . . Xiang, Y. T. (2016). Antipsychotic polypharmacy and quality of life in patients with schizophrenia treated in primary care in china.*54*, 36-42.

355. Hou, C. L., Zang, Y., Ma, X. R., Cai, M. Y., Li, Y., Jia, F. J., . . . Xiang, Y. T. (2016). The relationship between sleep patterns, quality of life, and social and clinical characteristics in chinese patients with schizophrenia.

356. Hou, C. L., Zang, Y., Rosen, R. C., Cai, M. Y., Li, Y., Jia, F. J., . . . Xiang, Y. T. (2016). Sexual dysfunction and its impact on quality of life in chinese patients with schizophrenia treated in primary care.*65*, 116-121.

357. Houghton, D. C., Maas, J., Twohig, M. P., Saunders, S. M., Compton, S. N., Neal-Barnett, A. M., . . . Woods, D. W. (2016). Comorbidity and quality of life in adults with hair pulling disorder.*239*, 12-19.

358. Houtveen, J. H., van Broeckhuysen-Kloth, S., Lintmeijer, L. L., Buhring, M. E. F., & Geenen, R. (2015). Intensive multidisciplinary treatment of severe somatoform disorder: A prospective evaluation.*203*, 141-148.

359. Hoy-Ellis, C. P., Shiu, C., Sullivan, K. M., Kim, H., Sturges, A. M., & Fredriksen-Goldsen, K. I. (2017). Prior military service, identity stigma, and mental health among transgender older adults.*57*, S63-S71.

360. Hsiao, C. Y., Hsieh, M. H., Tseng, C. J., Chien, S. H., & Chang, C. C. (2012). Quality of life of individuals with schizophrenia living in the community: Relationship to socio-demographic, clinical and psychosocial characteristics.*21*, 2367-2376.

361. Hsiao, C. Y., Lu, H. L., & Tsai, Y. F. (2017). Effect of family sense of coherence on internalized stigma and health-related quality of life among individuals with schizophrenia.

362. Hsiao, Y. Y., Shih, C. L., Yu, W. H., Hsieh, C. H., & Hsieh, C. L. (2015). Examining unidimensionality and improving reliability for the eight subscales of the SF-36 in opioid-dependent patients using rasch analysis.*24*, 279-285.

363. Huang, C. J., Huang, Y. H., & Lin, C. H. (2017). Factors related to the changes in quality of life for patients with depression after an acute course of electroconvulsive therapy.*33*, 126-133.

364. Huang, C. Y., Hsu, M. C., & Chen, T. J. (2012). An exploratory study of religious involvement as a moderator between anxiety, depressive symptoms and quality of life outcomes of older adults.*21*, 609-619.

365. Huang, R. R., Chen, Y. S., Chen, C. C., Chou, F. H. C., Su, S. F., Chen, M. C., . . . Chang, L. H. (2012). Quality of life and its associated factors among patients with two common types of chronic mental illness living in kaohsiung city.*66*, 482-490.

366. Huang, Y. S., Yeh, C. B., Tang, C. S., Chen, C. K., Chou, W. J., Chou, M. C., . . . Wang, L. J. (2013). Effectiveness of aripiprazole in adolescents and young adults with schizophrenia spectrum disorders: Comparison of first-episode to recurrent psychosis.*7*, 89-93.

367. Hughes, T., Cardno, A., West, R., Marino-Francis, F., Featherstone, I., Rolling, K., . . . House, A. (2016). Unrecognised bipolar disorder among UK primary care patients prescribed antidepressants: An observational study.*66*, e71-77.

368. Hui, C., Morcillo, C., Russo, D. A., Stochl, J., Shelley, G. F., Painter, M., . . . Perez, J. (2013). Psychiatric morbidity, functioning and quality of life in young people at clinical high risk for psychosis.*148*, 175-180.

369. Huijts, I., Kleijn, W. C., van Emmerik, A. A., Noordhof, A., & Smith, A. J. (2012). Dealing with man-made trauma: The relationship between coping style, posttraumatic stress, and quality of life in resettled, traumatized refugees in the netherlands.*25*, 71-78.

370. Hung, C. I., Liu, C. Y., & Wang, S. J. (2013). Migraine predicts physical and pain symptoms among psychiatric outpatients.*14*, 19.

371. Hung, C. I., Liu, C. Y., Yang, C. H., & Wang, S. J. (2012). Negative impact of migraine on quality of life after 4 weeks of treatment in patients with major depressive disorder.*66*, 8-16.

372. Hwang, S. S. H., Kim, E. Y., Kim, S. H., Kim, Y. S., & Ahn, Y. M. (2013). Intercultural differences in factor structure of the SWN-20 in patients with schizophrenia.*54*, 970-973.

373. Iglesias-GarcÃa, C., & Prieto, R. (2012). Quality of life in depressed women over 40 years old.*40*, 221-227.

374. Imam, M. A., Salam, M. A., Algin, S., & Ali, M. (2013). Quality of life in patients with major depressive disorder.*22*, 222-228.

375. Ishak, W. W., Balayan, K., Bresee, C., Greenberg, J. M., Fakhry, H., Christensen, S., & Rapaport, M. H. (2013). A descriptive analysis of quality of life using patient-reported measures in major depressive disorder in a naturalistic outpatient setting.*22*, 585-596.

376. IsHak, W. W., Christensen, S., Sayer, G., Ha, K., Li, N., Miller, J., . . . Cohen, R. M. (2013). Sexual satisfaction and quality of life in major depressive disorder before and after treatment with citalopram in the STAR*D study.*74*, 256-261.

377. IsHak, W. W., Greenberg, J. M., & Cohen, R. M. (2013). Predicting relapse in major depressive disorder using patient-reported outcomes of depressive symptom severity, functioning, and quality of life in the individual burden of illness index for depression (IBI-D).*151*, 59-65.

378. IsHak, W. W., Mirocha, J., Christensen, S., Wu, F., Kwock, R., Behjat, J., . . . Elashoff, D. (2014). Patient-reported outcomes of quality of life, functioning, and depressive symptom severity in major depressive disorder comorbid with panic disorder before and after ssri treatment in the STAR*D trial.*31*, 707-716.

379. Ishak, W. W., Mirocha, J., James, D., Tobia, G., Vilhauer, J., Fakhry, H., . . . Cohen, R. M. (2015). Quality of life in major depressive disorder before/after multiple steps of treatment and one-year follow-up.*131*, 51-60.

380. IsHak, W. W., Mirocha, J., Pi, S., Tobia, G., Becker, B., Peselow, E. D., & Cohen, R. M. (2014). Patient-reported outcomes before and after treatment of major depressive disorder.*16*, 171-183.

381. Ising, H. K., Lokkerbol, J., Rietdijk, J., Dragt, S., Klaassen, R. M., Kraan, T., . . . van der Gaag, M. (2017). Four-year cost-effectiveness of cognitive behavior therapy for preventing first-episode psychosis: The dutch early detection intervention evaluation (EDIE-NL) trial.*43*, 365-374.

382. Isitt, J. J., Nadipelli, V. R., Kouassi, A., Fava, M., & Heidbreder, C. (2016). Health-related quality of life in acute schizophrenia patients treated with RBP-7000 once monthly risperidone: An 8-week, randomized, double-blind, placebo-controlled, multicenter phase 3 study.*174*, 126-131.

383. Isjanovski, V., Naumovska, A., Bonevski, D., & Novotni, A. (2016). Validation of the schizophrenia quality of life scale revision 4 (SQLS-R4) among patients with schizophrenia.*4*, 65-69.

384. Iskandar, S., Van Crevel, R., Hidayat, T., Siregar, I. M. P., Achmad, T. H., Van Der Ven, A. J., & De Jong, C. A. (2013). Severity of psychiatric and physical problems is associated with lower quality of life in methadone patients in indonesia.*22*, 425-431.

385. Isralowitz, R., Reznik, A., & Pruginin, I. (2016). Quality of life among former soviet union and israeli origin methadone users.*15*, 425-433.

386. Ito, S., Nemoto, T., Tsujino, N., Ohmuro, N., Matsumoto, K., Matsuoka, H., . . . Mizuno, M. (2015). Differential impacts of duration of untreated psychosis (DUP) on cognitive function in first-episode schizophrenia according to mode of onset.*30*, 995-1001.

387. Jacoby, R. J., Leonard, R. C., Riemann, B. C., & Abramowitz, J. S. (2014). Predictors of quality of life and functional impairment in obsessive-compulsive disorder.*55*, 1195-1202.

388. Jalenques, I., Galland, F., Malet, L., Morand, D., Legrand, G., Auclair, C., . . . Durif, F. (2012). Quality of life in adults with gilles de la tourette syndrome.*12*

389. Janicak, P. G., Dunner, D. L., Aaronson, S. T., Carpenter, L. L., Boyadjis, T. A., Brock, D. G., . . . Demitrack, M. A. (2013). Transcranial magnetic stimulation (TMS) for major depression: A multisite, naturalistic, observational study of quality of life outcome measures in clinical practice.*18*, 322-332.

390. Jansen, K., Campos Mondin, T., Azevedo Cardoso, T. D., Costa Ores, L. D., De Mattos Souza, L. D., Tavares Pinheiro, R., . . . Da Silva, R. A. (2013). Quality of life and mood disorder episodes: Community sample.*147*, 123-127.

391. Jaracz, K., Gorna, K., Kiejda, J., Grabowska-Fudala, B., Jaracz, J., Suwalska, A., & Rybakowski, J. K. (2015). Psychosocial functioning in relation to symptomatic remission: A longitudinal study of first episode schizophrenia.*30*, 907-913.

392. Javadpour, A., Hedayati, A., Dehbozorgi, G. R., & Azizi, A. (2013). The impact of a simple individual psycho-education program on quality of life, rate of relapse and medication adherence in bipolar disorder patients.*6*, 208-213.

393. Jenkins, P. E., Rienecke, R. D., Conley, C. S., Meyer, C., & Blissett, J. M. (2015). The relation between eating disorder symptoms and impairment.*203*, 452-458.

394. Jeon, G. S., Gang, M., & Oh, K. (2017). The effectiveness of the nanta-program on psychiatric symptoms, interpersonal relationships, and quality of life in forensic inpatients with schizophrenia.*31*, 93-98.

395. Jeon, H. J., Walker, R. S., Inamori, A., Hong, J. P., Cho, M. J., Baer, L., . . . Mischoulon, D. (2014). Differences in depressive symptoms between korean and american outpatients with major depressive disorder.*29*, 150-156.

396.Jha, M. K., Greer, T. L., Grannemann, B. D., Carmody, T., Rush, A. J., & Trivedi, M. H. (2016). Early normalization of quality of life predicts later remission in depression: Findings from the CO-MED trial.*206*, 17-22.

397.Jha, M. K., Minhajuddin, A., Thase, M. E., & Jarrett, R. B. (2014). Improvement in self-reported quality of life with cognitive therapy for recurrent major depressive disorder.*167*, 37-43.

398.Johanson, S., & Bejerholm, U. (2017). The role of empowerment and quality of life in depression severity among unemployed people with affective disorders receiving mental healthcare.*39*, 1807-1813.

399.Johansson, R., Carlbring, P., Heedman, A., Paxling, B., & Andersson, G. (2013). Depression, anxiety and their comorbidity in the swedish general population: Point prevalence and the effect on health-related quality of life.*2013*

400.Johnson, S. L., Tharp, J. A., Peckham, A. D., & McMaster, K. J. (2016). Emotion in bipolar I disorder: Implications for functional and symptom outcomes.*125*, 40-52.

401.Juarascio, A. S., Schumacher, L. M., Shaw, J., Forman, E. M., & Herbert, J. D. (2015). Acceptance-based treatment and quality of life among patients with an eating disorder.*4*, 42-47.

402.Juel, A., Kristiansen, C. B., Madsen, N. J., Munk-JÃ¸rgensen, P., & Hjorth, P. (2017). Interventions to improve lifestyle and quality-of-life in patients with concurrent mental illness and substance use.*71*, 197-204.

403.Jung, M., & Han, K. (2017). Effectiveness of gratitude disposition promotion program on depression and quality of life of chronic schizophrenic patients.*59*, 189-195.

404.Jung, S. H., & Kim, H. J. (2012). Perceived stigma and quality of life of individuals diagnosed with schizophrenia and receiving psychiatric rehabilitation services: A comparison between the clubhouse model and a rehabilitation skills training model in south korea.*35*, 460-465.

405.Jung, Y. E., Seo, H. J., Song, H. R., Woo, Y. S., Yim, H. W., Sung, H. M., . . . Jun, T. Y. (2012). Factors associated with subjective quality of life in korean patients with depressive disorders: The CRESCEND study.*21*, 967-974.

406.Juretic, T. G., RuÅ□ic, K., Letica-Crepulja, M., Petric, D., Dadic-Hero, E., & FranciÅ¡kovic, T. (2016). Effects of psychosocial day care programme on quality of life in patients affected with schizophrenia-a prospective study.*28*, 111-117.

407. Jutkowitz, E., Pizzi, L., Hess, E., Suh, D. C., & Gitlin, L. N. (2013). Comparison of three societally derived health-state classification values among older african americans with depressive symptoms.*22*, 1491-1498.

408. KÃ¶hler, S., Unger, T., Hoffmann, S., Mackert, A., Ross, B., & Fydrich, T. (2015). The relationship of health-related quality of life and treatment outcome during inpatient treatment of depression.*24*, 641-649.

409. Kako, Y., Ito, K., Hashimoto, N., Toyoshima, K., Shimizu, Y., Mitsui, N., . . . Kusumi, I. (2014). The relationship between insight and subjective experience in schizophrenia.*10*

410. Kallestad, H., Hansen, B., Langsrud, K., Ruud, T., Morken, G., Stiles, T. C., & Grawe, R. W. (2012). Impact of sleep disturbance on patients in treatment for mental disorders.*12*, 179.

411. Kaltsatou, A., Kouidi, E., Fountoulakis, K., Sipka, C., Theochari, V., Kandylis, D., & Deligiannis, A. (2015). Effects of exercise training with traditional dancing on functional capacity and quality of life in patients with schizophrenia: A randomized controlled study.*29*, 882-891.

412. Kamal, R. M., Dijkstra, B. A. G., de Weert-van Oene, G. H., van Duren, J. A. M., & de Jong, C. A. J. (2017). Psychiatric comorbidity, psychological distress, and quality of life in gamma-hydroxybutyrate-dependent patients.*36*, 72-79.

413. Kamaradova, D., Latalova, K., Prasko, J., Grambal, A., Sigmundova, Z., Kasalova, P., & Cakirpaloglu, S. (2016). Minimal role of comorbid personality disorder on the quality of life in patients with anxiety spectrum disorders.*37*, 559-566.

414. Kamio, Y., Inada, N., & Koyama, T. (2013). A nationwide survey on quality of life and associated factors of adults with high-functioning autism spectrum disorders.*17*, 15-26.

415. Kane, J. M., Robinson, D. G., Schooler, N. R., Mueser, K. T., Penn, D. L., Rosenheck, R. A., . . . Heinssen, R. K. (2016). Comprehensive versus usual community care for first-episode psychosis: 2-year outcomes from the NIMH RAISE early treatment program.*173*, 362-372.

416. Kang, E., Choe, A. Y., Kim, B., Lee, J. Y., Choi, T. K., Na, H. R., & Lee, S. H. (2016). Serotonin transporter and COMT polymorphisms as independent predictors of health-related quality of life in patients with panic disorder.*31*, 757-763.

417. Kang, E. H., Kim, B., Choe, A. Y., Lee, J. Y., Choi, T. K., & Lee, S. H. (2015). Panic disorder and health-related quality of life: The predictive roles of anxiety sensitivity and trait anxiety.*225*, 157-163.

418.Kang, N. R., Kim, M. D., Lee, C. I., Kwak, Y. S., Choi, K. M., Im, H. J., & Park, J. H. (2012). The influence of subcortical ischemic lesions on cognitive function and quality of life in late life depression.*136*, 485-490.

419.Kao, C. C., & Huang, H. M. (2014). A comparison of the quality of life of patients with schizophrenia in daycare and homecare settings.*22*, 126-135.

420.Kao, Y. C., Liu, Y. P., Cheng, T. H., & Chou, M. K. (2012). Subjective quality of life and suicidal behavior among taiwanese schizophrenia patients.*47*, 523-532.

421.Karayagiz, S., & Basturk, M. (2016). Alexithymia levels in patients with unipolar and bipolar depression and the effect of alexithymia on both severity of depression symptoms and quality of life.*17*, 362-368.

422.Katz, N., Gilad Izhaky, S., Ziv, O., & Revach, A. (2013). "Coffee stands": A vocational rehabilitation project in the community for people coping with mental disorders.*44*, 481-490.

423.Kaviani, H., Hatami, N., & Javaheri, F. (2012). The impact of mindfulness-based cognitive therapy (MBCT) on mental health and quality of life in a sub-clinically depressed population., 21-28.

424.Kavitha, C., Rangan, U., & Nirmalan, P. K. (2014). Quality of life and marital adjustment after cognitive behavioural therapy and behavioural marital therapy in couples with anxiety disorders.*8*, WC01-WC04.

425.Kearney, D. J., McDermott, K., Malte, C., Martinez, M., & Simpson, T. L. (2012). Association of participation in a mindfulness program with measures of PTSD, depression and quality of life in a veteran sample.*68*, 101-116.

426.Kelly, P. J., Robinson, L. D., Baker, A. L., Deane, F. P., McKetin, R., Hudson, S., & Keane, C. (2017). Polysubstance use in treatment seekers who inject amphetamine: Drug use profiles, injecting practices and quality of life.*71*, 25-30.

427.Kerling, A., Tegtbur, U., Ziegenbein, M., Grams, L., Heinze, D. R., & Sieberer, M. (2013). Exercise capacity and quality of life in patients with schizophrenia.*84*, 417-427.

428.Kerridge, B. T., Pickering, R., Chou, P., Saha, T. D., & Hasin, D. S. (2018). DSM-5 cannabis use disorder in the national epidemiologic survey on alcohol and related conditions-III: Gender-specific profiles.*76*, 52-60.

429.Keshavarzi, S., Ahmadi, S. M., & Lankarani, K. B. (2015). The impact of depression and malnutrition on health-related quality of life among the elderly iranians.*7*, 161-170.

430. Khajedaluee, M., Assadi, R., & Dadgar Moghadam, M. (2013). Health-related quality of life of young addict women in mashhad, IR iran.*2*, 87-91.

431. Khanna, R., Jariwala, K., & West-Strum, D. (2015). Validity and reliability of the medical outcomes study short-form health survey version 2 (SF-12v2) among adults with autism.*43-44*, 51-60.

432. Khanna, R., Jariwala-Parikh, K., West-Strum, D., & Mahabaleshwarkar, R. (2014). Health-related quality of life and its determinants among adults with autism.*8*, 157-167.

433. Killaspy, H., Priebe, S., Bremner, S., McCrone, P., Dowling, S., Harrison, I., . . . King, M. (2016). Quality of life, autonomy, satisfaction, and costs associated with mental health supported accommodation services in england: A national survey.*3*, 1129-1137.

434. Killaspy, H., White, S., Lalvani, N., Berg, R., Thachil, A., Kallumpuram, S., . . . Mezey, G. (2014). The impact of psychosis on social inclusion and associated factors.*60*, 148-154.

435. Kim, J. E., Song, I. H., & Lee, S. H. (2017). Gender differences of stressful life events, coping style, symptom severity, and health-related quality of life in patients with panic disorder.

436. Kim, J. H., Lee, S., Han, A. Y., Kim, K., & Lee, J. (2015). Relationship between cognitive insight and subjective quality of life in outpatients with schizophrenia.*11*, 2041-2048.

437. Kim, J. M., Chalem, Y., di Nicola, S., Hong, J. P., Won, S. H., & Milea, D. (2016). A cross-sectional study of functional disabilities and perceived cognitive dysfunction in patients with major depressive disorder in south korea: The PERFORM-K study.*239*, 353-361.

438. Kim, W. H., Jung, D. Y., Lee, J. Y., Chang, S. M., Jeon, H. J., Lee, J. Y., . . . Hahm, B. J. (2017). Lifetime prevalence of psychiatric morbidities, suicidality, and quality of life in a community population with the bimodal chronotype: A nationwide epidemiologic study.*34*, 732-739.

439. Kim, Y. S., Cha, B., Lee, D., Kim, S. M., Moon, E., Park, C. S., . . . Lee, S. (2013). The relationship between impulsivity and quality of life in euthymic patients with bipolar disorder.*10*, 246-252.

440. Kirouac, M., Stein, E. R., Pearson, M. R., & Witkiewitz, K. (2017). Viability of the world health organization quality of life measure to assess changes in quality of life following treatment for alcohol use disorder., 1-11.

441. Knaevelsrud, C., Bottche, M., Pietrzak, R. H., Freyberger, H. J., Renneberg, B., & Kuwert, P. (2014). Integrative testimonial therapy: An internet-based, therapist-assisted therapy for german elderly survivors of the world war II with posttraumatic stress symptoms.*202*, 651-658.

442. Koc, I., & Kesebir, S. (2014). The subgroup effect of adult attention deficit hyperactivity on disability and quality of life among bipolar patients.*24*, 49-54.

443. Kohigashi, M., Kitabayashi, Y., Okamura, A., Nakamura, M., Hoshiyama, A., Kunizawa, M., . . . Fukui, K. (2013). Relationship between patients' quality of life and coercion in psychiatric acute wards.*208*, 88-90.

444. KokaÃ§ya, M. H., Virit, O., Ã‡Ã¶poÄŸlu Ãœ, S., SavaÅŸ, H., Ari, M., & BahÃ§eci, B. (2016). Symptomatic remission determines functional improvement and quality of life in schizophrenia.*53*, 328-333.

445. Kong, C. L., Lee, C. C., Ip, Y. C., Chow, L. P., Leung, C. H., & Lam, Y. C. (2016). Validation of the hong kong cantonese version of world health organization five well-being index for people with severe mental illness.*26*, 18-21.

446. Korte, J., Bohlmeijer, E., Cappeliez, P., Smit, F., & Westerhof, G. (2012). Life review therapy for older adults with moderate depressive symptomatology: A pragmatic randomized controlled trial.*42*, 1163-1173.

447. Kortrijk, H. E., Mulder, C. L., Van Der Gaag, M., & Wiersma, D. (2012). Symptomatic and functional remission and its associations with quality of life in patients with psychotic disorder in assertive community treatment teams.*53*, 1174-1180.

448. Krebs, E., Kerr, T., Wood, E., & Nosyk, B. (2016). Characterizing long-term health related quality of life trajectories of individuals with opioid use disorder.*67*, 30-37.

449. Kuga, A., Tsuji, T., Hayashi, S., Fujikoshi, S., Tokuoka, H., Yoshikawa, A., . . . Azekawa, T. (2017). An observational study of duloxetine versus SSRI monotherapy in japanese patients with major depressive disorder: Subgroup analyses of treatment effectiveness for pain, depressive symptoms, and quality of life.*13*, 2115-2124.

450. Kugler, B. B., Lewin, A. B., Phares, V., Geffken, G. R., Murphy, T. K., & Storch, E. A. (2013). Quality of life in obsessive-compulsive disorder: The role of mediating variables.*206*, 43-49.

451.Kugler, B. B., Phares, V., Salloum, A., & Storch, E. A. (2016). The role of anxiety sensitivity in the relationship between posttraumatic stress symptoms and negative outcomes in trauma-exposed adults.*29*, 187-201.

452.Kukla, M., Lysaker, P. H., & Roe, D. (2014). Strong subjective recovery as a protective factor against the effects of positive symptoms on quality of life outcomes in schizophrenia.*55*, 1363-1368.

453.Kumar, A., Sharma, M. P., Kandavel, T., & Janardhan Reddy, Y. C. (2012). Cognitive appraisals and quality of life in patients with obsessive compulsive disorder.*1*, 301-305.

454.Kumazaki, H., Kobayashi, H., Niimura, H., Kobayashi, Y., Ito, S., Nemoto, T., . . . Mizuno, M. (2012). Lower subjective quality of life and the development of social anxiety symptoms after the discharge of elderly patients with remitted schizophrenia: A 5-year longitudinal study.*53*, 946-951.

455.Kuokkanen, R., Aho-Mustonen, K., Muotka, J., Lappalainen, R., & Tiihonen, J. (2015). A pilot study of group administered metacognitive training (MCT) for schizophrenia patients in a high-security forensic setting: Subjective training success and health-related quality of life.*15*, 344-362.

456.Kurtz, M. M., Bronfeld, M., & Rose, J. (2012). Cognitive and social cognitive predictors of change in objective versus subjective quality-of-life in rehabilitation for schizophrenia.*200*, 102-107.

457.Kusumi, I., Honda, M., Uemura, K., Sugawara, Y., Kohsaka, M., Tochigi, A., & Koyama, T. (2012). Effect of olanzapine orally disintegrating tablet versus oral standard tablet on body weight in patients with schizophrenia: A randomized open-label trial.*36*, 313-317.

458.Kwong, V. W. Y., Chang, W. C., Chan, G. H. K., Jim, O. T. T., Lau, E. S. K., Hui, C. L. M., . . . Chen, E. Y. H. (2017). Clinical and treatment-related determinants of subjective quality of life in patients with first-episode psychosis.*249*, 39-45.

459.LÃ□pez, E., Steiner, A. J., Manier, K., Shapiro, B. B., Vanle, B., Parisi, T., . . . William IsHak, W. (2018). Quality of life and functioning of hispanic patients with major depressive disorder before and after treatment.*225*, 117-122.

460.LÃ□pez-Navarro, E., Del Canto, C., Belber, M., Mayol, A., FernÃ¡ndez-Alonso, O., Lluis, J., . . . Chadwick, P. (2015). Mindfulness improves psychological quality of life in community-based patients with severe mental health problems: A pilot randomized clinical trial.*168*, 530-536.

461.LÃ□pez-Navarro, E., del Canto, C., Mayol, A., FernÃ¡ndez-Alonso, O., & Munar, E. (2017). Psychotic symptoms and quality of life: A mediation analysis of daily-life coping.

462.Laaksonen, E., Vuoristo-Myllys, S., Koski-Jannes, A., & Alho, H. (2013). Combining medical treatment and CBT in treating alcohol-dependent patients: Effects on life quality and general well-being.*48*, 687-693.

463.Lai, Y. C., Huang, M. C., Chen, H. C., Lu, M. K., Chiu, Y. H., Shen, W. W., . . . Kuo, P. H. (2014). Familiality and clinical outcomes of sleep disturbances in major depressive and bipolar disorders.*76*, 61-67.

464.Lambri, M., Chakraborty, A., Leavey, G., & King, M. (2012). Quality of life and unmet need in people with psychosis in the london borough of haringey, UK.*2012*

465.Landolt, K., RÃ¶ssler, W., Burns, T., Ajdacic-Gross, V., Galderisi, S., Libiger, J., . . . Fleischhacker, W. W. (2012). The interrelation of needs and quality of life in first-episode schizophrenia.*262*, 207-216.

466.Landolt, K., RÃ¶ssler, W., Burns, T., Ajdacic-Gross, V., Galderisi, S., Libiger, J., . . . Group, E. S. (2012). Unmet needs in patients with first-episode schizophrenia: A longitudinal perspective.*42*, 1461-1473.

467.Lanfredi, M., Candini, V., Buizza, C., Ferrari, C., Boero, M. E., Giobbio, G. M., . . . Zorzella, L. (2014). The effect of service satisfaction and spiritual well-being on the quality of life of patients with schizophrenia.*216*, 185-191.

468.Langle, G., Steinert, T., Weiser, P., Schepp, W., Jaeger, S., Pfiffner, C., . . . Kilian, R. (2012). Effects of polypharmacy on outcome in patients with schizophrenia in routine psychiatric treatment.*125*, 372-381.

469.Lao, C. K., Chan, Y. M., Tong, H. H. Y., & Chan, A. (2016). Underdiagnosis of depression in an economically deprived population in macao, china.*8*, 70-79.

470.Lariviere, N., Denis, C., Payeur, A., Ferron, A., Levesque, S., & Rivard, G. (2016). Comparison of objective and subjective life balance between women with and without a personality disorder.*87*, 663-673.

471.Las Hayas, C., Padilla, P., Del Barrio, A. G., Beato-Fernandez, L., MuÃ±oz, P., & GÃ¡mez-Guadix, M. (2016). Individualised versus standardised assessment of quality of life in eating disorders.*24*, 147-156.

472.Lasebikan, V. O., & Owoaje, E. T. (2015). Quality of life in psychosis: Prevalence and associated factors in a nigerian clinical population.*51*, 491-496.

473.Latner, J. D., Mond, J. M., Vallance, J. K., Gleaves, D. H., & Buckett, G. (2013). Quality of life impairment and the attitudinal and behavioral features of eating disorders.*201*, 592-597.

474. Laurenssen, E. M. P., Eeren, H. V., Kikkert, M. J., Peen, J., Westra, D., Dekker, J. J. M., & Busschbach, J. J. V. (2016). The burden of disease in patients eligible for mentalization-based treatment (MBT): Quality of life and costs.*14*

475. Laxhman, N., Greenberg, L., & Priebe, S. (2017). Satisfaction with sex life among patients with schizophrenia.

476. Le, Q. A., Doctor, J. N., Zoellner, L. A., & Feeny, N. C. (2014). Cost-effectiveness of prolonged exposure therapy versus pharmacotherapy and treatment choice in posttraumatic stress disorder (the optimizing PTSD treatment trial): A doubly randomized preference trial.*75*, 222-230.

477. Lee, D., Cha, B., Park, C. S., Kim, B. J., Lee, C. S., Lee, S. J., . . . Choi, J. W. (2017). Effects of resilience on quality of life in patients with bipolar disorder.*207*, 434-441.

478. Lee, E. H. M., Hui, C. L. M., Lin, J. J. X., Ching, E. Y. N., Chang, W. C., Chan, S. K. W., & Chen, E. Y. H. (2016). Quality of life and functioning in first-episode psychosis chinese patients with different antipsychotic medications.*10*, 535-539.

479. Lee, Y. T., Liu, S. I., Huang, H. C., Sun, F. J., Huang, C. R., & Yeung, A. (2014). Validity and reliability of the chinese version of the short form of quality of life enjoyment and satisfaction questionnaire (Q-LES-Q-SF).*23*, 907-916.

480. Lehenbauer-Baum, M., Klaps, A., Kovacovsky, Z., Witzmann, K., Zahlbruckner, R., & Stetina, B. U. (2015). Addiction and engagement: An explorative study toward classification criteria for internet gaming disorder.*18*, 343-349.

481. Lenox-Smith, A., Macdonald, M. T. B., Reed, C., Tylee, A., Peveler, R., Quail, D., & Wildgust, H. J. (2013). Quality of life in depressed patients in UK primary care: The FINDER study.*2*, 25-42.

482. Leung, S. F., Ma, J. L., & Russell, J. (2013). Enhancing quality of life in people with disordered eating using an online self-help programme.*1*, 9.

483. Lev-Ran, S., Imtiaz, S., Taylor, B. J., Shield, K. D., Rehm, J., & Le Foll, B. (2012). Gender differences in health-related quality of life among cannabis users: Results from the national epidemiologic survey on alcohol and related conditions.*123*, 190-200.

484. Lev-Ran, S., Le Foll, B., McKenzie, K., & Rehm, J. (2012). Cannabis use and mental health-related quality of life among individuals with anxiety disorders.*26*, 799-810.

485.Lewis, J. E., Tiozzo, E., Melillo, A. B., Leonard, S., Chen, L., Mendez, A., . . . Konefal, J. (2013). The effect of methylated vitamin B complex on depressive and anxiety symptoms and quality of life in adults with depression.*2013*, 621453.

486.Li, I. C., Kuo, H. T., Lin, K. C., & Wu, Y. C. (2014). The effects of depressive symptoms on quality of life among institutionalized older adults in taiwan.*50*, 58-64.

487.Li, J., Mao, J., Du, Y., Morris, J. L., Gong, G., & Xiong, X. (2012). Health-related quality of life among pregnant women with and without depression in hubei, china.*16*, 1355-1363.

488.Li, Q., Xiang, Y. T., Su, Y. A., Shu, L., Yu, X., Chiu, H. F. K., . . . Si, T. M. (2015). Antipsychotic polypharmacy in schizophrenia patients in china and its association with treatment satisfaction and quality of life: Findings of the third national survey on use of psychotropic medications in china.*49*, 129-136.

489.Li, Q., Xiang, Y. T., Su, Y. A., Shu, L., Yu, X., Correll, C. U., . . . Kane, J. M. (2015). Clozapine in schizophrenia and its association with treatment satisfaction and quality of life: Findings of the three national surveys on use of psychotropic medications in china (2002-2012).*168*, 523-529.

490.Li, X. H., An, F. R., Ungvari, G. S., Ng, C. H., Chiu, H. F. K., Wu, P. P., . . . Xiang, Y. T. (2017). Prevalence of smoking in patients with bipolar disorder, major depressive disorder and schizophrenia and their relationships with quality of life.*7*, 8430.

491.Li, Y., Hou, C. L., Ma, X. R., Zang, Y., Jia, F. J., Zhong, B. L., . . . Xiang, Y. T. (2016). Smoking and its associations with sociodemographic and clinical characteristics and quality of life in patients with schizophrenia treated in primary care in china.*38*, 79-83.

492.Li, Y., Hou, C. L., Ma, X. R., Zhong, B. L., Zang, Y., Jia, F. J., . . . Xiang, Y. T. (2017). Quality of life in chinese patients with schizophrenia treated in primary care.*254*, 80-84.

493.Liao, Y. T., Chen, C. Y., Ng, M. H., Huang, K. Y., Shao, W. C., Lin, T. Y., . . . Gossop, M. (2017). Depression and severity of substance dependence among heroin dependent patients with ADHD symptoms.*26*, 26-33.

494.Lichtenstein, M. B., Christiansen, E., Elklit, A., Bilenberg, N., & StÃ¸ving, R. K. (2014). Exercise addiction: A study of eating disorder symptoms, quality of life, personality traits and attachment styles.*215*, 410-416.

495.Lien, Y. J., Chang, H. A., Kao, Y. C., Tzeng, N. S., Lu, C. W., & Loh, C. H. (2016). Insight, self-stigma and psychosocial outcomes in schizophrenia: A structural equation modelling approach., 1-10.

496.Lien, Y. J., Chang, H. A., Kao, Y. C., Tzeng, N. S., Lu, C. W., & Loh, C. H. (2017). The impact of cognitive insight, self-stigma, and medication compliance on the quality of life in patients with schizophrenia., 1-12.

497.Lim, J. A., Lee, J. Y., Jung, H. Y., Sohn, B. K., Choi, S. W., Kim, Y. J., . . . Choi, J. S. (2016). Changes of quality of life and cognitive function in individuals with internet gaming disorder: A 6-month follow-up.*95*, e5695.

498.Lim, L., Jin, A. Z., & Ng, T. P. (2012). Anxiety and depression, chronic physical conditions, and quality of life in an urban population sample study.*47*, 1047-1053.

499.Lim, M. H., Gleeson, J. F., Jackson, H. J., & Fernandez, K. C. (2014). Social relationships and quality of life moderate distress associated with delusional ideation.*49*, 97-107.

500.Lin, C. H., Yen, Y. C., Chen, M. C., & Chen, C. C. (2013). Relief of depression and pain improves daily functioning and quality of life in patients with major depressive disorder.*47*, 93-98.

501.Lin, C. H., Yen, Y. C., Chen, M. C., & Chen, C. C. (2014). Depression and pain impair daily functioning and quality of life in patients with major depressive disorder.*166*, 173-178.

502.Lin, C. Y., Chang, K. C., Wang, J. D., & Lee, L. J. H. (2016). Quality of life and its determinants for heroin addicts receiving a methadone maintenance program: Comparison with matched referents from the general population.*115*, 714-727.

503.Lin, C., Chang, C., Wu, T., & Wang, J. (2016). Dynamic changes of self-stigma, quality of life, somatic complaints, and depression among people with schizophrenia: A pilot study applying kernel smoothers.*1*, 29-43.

504.Lin, L. Y. (2014). Quality of life of taiwanese adults with autism spectrum disorder.*9*

505.Lin, L. A. (2012). Comparing antipsychotic treatments for schizophrenia: A health state approach.*73*, 863.

506.Lin, S. H., Chen, K. C., Lee, S. Y., Hsiao, C. Y., Lee, I. H., Yeh, T. L., . . . Yang, Y. K. (2013). The economic cost of heroin dependency and quality of life among heroin users in taiwan.*209*, 512-517.

507.Lin, S. P., Liu, C. Y., & Yang, C. Y. (2017). Relationship between lifestyles that promote health and quality of life in patients with chronic schizophrenia: A cross-sectional study.

508.Lin, Y. J., Lo, K. W., Yang, L. K., & Gau, S. S. F. (2015). Validation of DSM-5 age-of-onset criterion of attention deficit/hyperactivity disorder (ADHD) in adults: Comparison of life quality, functional impairment, and family function.*47*, 48-60.

509.Lindkvist, M., & Feldman, I. (2016). *Assessing outcomes for cost-utility analysis in mental health interventions: Mapping mental health specific outcome measure GHQ-12 onto EQ-5D-3L*

510.Lindner, P., Andersson, G., Ã–st, L. G., & Carlbring, P. (2013). Validation of the internet-administered quality of life inventory (QOLI) in different psychiatric conditions.*42*, 315-327.

511.Livingston, J. (2012). Self-stigma and quality of life among people with mental illness who receive compulsory community treatment services.*40*, 699-714.

512.Llorca, P. M., Blanc, O., Samalin, L., Bosia, M., Cavallaro, R., & Initiative, E. (2012). Factors involved in the level of functioning of patients with schizophrenia according to latent variable modeling.*27*, 396-400.

513.Locklear, J. C., SvedsÃ□ter, H., Datto, C., & Endicott, J. (2013). Effects of once-daily extended release quetiapine fumarate (quetiapine XR) on quality of life and sleep in elderly patients with major depressive disorder.*149*, 189-195.

514.Loh, S. Y., Abdullah, A., Abu Bakar, A. K., Thambu, M., & Nik Jaafar, N. R. (2016). Structured walking and chronic institutionalized schizophrenia inmates: A pilot RCT study on quality of life.*8*, 238-248.

515.Lokkerbol, J., Adema, D., de Graaf, R., ten Have, M., Cuijpers, P., Beekman, A., & Smit, F. (2013). Non-fatal burden of disease due to mental disorders in the netherlands.*48*, 1591-1599.

516.Lozano Ã“, M., Rojas, A. J., & FernÃ¡ndez CalderÃ□n, F. (2017). Psychiatric comorbidity and severity of dependence on substance users: How it impacts on their health-related quality of life?*26*, 119-126.

517.Lua, P. L., & Talib, N. S. (2012). A 12-month evaluation of health-related quality of life outcomes of methadone maintenance program in a rural malaysian sample.*47*, 1100-1105.

518.Lua, P. L., & Talib, N. S. (2013). Auricular acupuncture for drug dependence: An open-label randomized investigation on clinical outcomes, health-related quality of life, and patient acceptability.*19*, 28-42.

519.Lucchetti, G., Lucchetti, A. L. G., de Bernardin Goncalves, J. P., & Vallada, H. P. (2015). Validation of the portuguese version of the functional assessment of chronic illness therapy-spiritual well-being scale (FACIT-sp 12) among brazilian psychiatric inpatients.*54*, 112-121.

520.Lugoboni, F., Mirijello, A., Faccini, M., Casari, R., Cossari, A., Musi, G., . . . Addolorato, G. (2014). Quality of life in a cohort of high-dose benzodiazepine dependent patients.*142*, 105-109.

521.Luo, Y. L., Heeramun-Aubeeluck, A., Huang, X., Ye, G., Wu, H., Sun, L., . . . Zhang, M. Y. (2014). Factors influencing quality of life in chinese patients with persistent somatoform pain disorder.*19*, 744-752.

522.Luquiens, A., Whalley, D., LaramÃ©e, P., Falissard, B., Kostogianni, N., Rehm, J., . . . Aubin, H. J. (2016). Validation of a new patient-reported outcome instrument of health-related quality of life specific to patients with alcohol use disorder: The alcohol quality of life scale (AQoLS).*25*, 1549-1560.

523.MacAulay, R., & Cohen, A. (2014). Self-conscious emotions' role in functional outcomes within clinical populations.*216*, 17-23.

524.Macbeth, A., Gumley, A., Schwannauer, M., & Fisher, R. (2015). Self-reported quality of life in a scottish first-episode psychosis cohort: Associations with symptomatology and premorbid adjustment.*9*, 53-60.

525.MacInnes, D., Kinane, C., Parrott, J., Mansfield, J., Craig, T., Eldridge, S., . . . Priebe, S. (2016). A pilot cluster randomised trial to assess the effect of a structured communication approach on quality of life in secure mental health settings: The comquol study.*16*

526.Mack, S., Jacobi, F., Beesdo-Baum, K., Gerschler, A., Strehle, J., HÃ¶fler, M., . . . Wittchen, H. U. (2015). Functional disability and quality of life decrements in mental disorders: Results from the mental health module of the german health interview and examination survey for adults (DEGS1-MH).*30*, 793-800.

527.Mackala, S. A., Torres, I. J., Kozicky, J., Michalak, E. E., & Yatham, L. N. (2014). Cognitive performance and quality of life early in the course of bipolar disorder.*168*, 119-124.

528.Magalhaes, P. V., Manzolli, P., Walz, J. C., & Kapczinski, F. (2012). A bidimensional solution for outcomes in bipolar disorder.*200*, 180-182.

529.Maisto, S. A., Roos, C. R., Hallgren, K. A., Moskal, D., Wilson, A. D., & Witkiewitz, K. (2016). Do alcohol relapse episodes during treatment predict long-term outcomes? investigating the validity of existing definitions of alcohol use disorder relapse.*40*, 2180-2189.

530.Makara-Studzinska, M., Wolyniak, M., & Krys, K. (2012). Influence of anxiety and depression on quality of life of people with schizophrenia in the eastern region of poland.*2012*, 839324.

531. Malhotra, N., Kulhara, P., Chakrabarti, S., & Grover, S. (2016). Lifestyle related factors & impact of metabolic syndrome on quality of life, level of functioning & self-esteem in patients with bipolar disorder & schizophrenia.*143*, 434-442.

532. Mangweth-Matzek, B., Hoek, H. W., Rupp, C. I., Lackner-Seifert, K., Frey, N., Whitworth, A. B., . . . Kinzl, J. (2014). Prevalence of eating disorders in middle-aged women.*47*, 320-324.

533. Manning, V., Gomez, B., Guo, S., Low, Y. D., Koh, P. K., & Wong, K. E. (2012). An exploration of quality of life and its predictors in patients with addictive disorders: Gambling, alcohol and drugs.*10*, 551-562.

534. Marceau, E. M., Berry, J., Lunn, J., Kelly, P. J., & Solowij, N. (2017). Cognitive remediation improves executive functions, self-regulation and quality of life in residents of a substance use disorder therapeutic community.*178*, 150-158.

535. Marcheschi, E., Laike, T., Brunt, D., Hansson, L., & Johansson, M. (2015). Quality of life and place attachment among people with severe mental illness.*41*, 145-154.

536. Marcon, S. R., Rubira, E. A., Espinosa, M. M., & Barbosa, D. A. (2012). Quality of life and depressive symptoms among caregivers and drug dependent people.*20*, 167-174.

537. Margariti, M., Ploumpidis, D., Economou, M., Christodoulou, G. N., & Papadimitriou, G. N. (2015). Quality of life in schizophrenia spectrum disorders: Associations with insight and psychopathology.*225*, 695-701.

538. Marini, M., Schnornberger, T. M., Brandalise, G. B., Bergozza, M., & Heldt, E. (2013). *Quality of life determinants in patients of a psychosocial care center for alcohol and other drug users*

539. MarinkoviÄ‡, M., DjordjeviÄ‡-JovanoviÄ‡, L., MiljkoviÄ‡, S., MilojkoviÄ‡, B., & JanjiÄ‡, V. (2017). Quality of life of treated opiate addicts in the methadone maintenance program and those treated with buprenorphine.*74*, 435-444.

540. Marino, L., Nossel, I., Choi, J. C., Nuechterlein, K., Wang, Y., Essock, S., . . . Dixon, L. (2015). The RAISE connection program for early psychosis: Secondary outcomes and mediators and moderators of improvement.*203*, 365-371.

541. Maripuu, M., Wikgren, M., Karling, P., Adolfsson, R., & Norrback, K. F. (2014). Relative hypo- and hypercortisolism are both associated with depression and lower quality of life in bipolar disorder: A cross-sectional study.*9*

542. Marques, L., Bui, E., LeBlanc, N., Porter, E., Robinaugh, D., Dryman, M. T., . . . Simon, N. (2013). Complicated grief symptoms in anxiety disorders: Prevalence and associated impairment.*30*, 1211-1216.

543. Marques, T. C., Sarracini, K. L., Cortellazzi, K. L., Mialhe, F. L., de Castro Meneghim, M., Pereira, A. C., & Ambrosano, G. M. (2015). The impact of oral health conditions, socioeconomic status and use of specific substances on quality of life of addicted persons.*15*, 38.

544. MartÃn, J., Padierna, A., LoroÃ±o, A., MuÃ±oz, P., & Quintana, J. M. (2017). Predictors of quality of life in patients with eating disorders.*45*, 182-189.

545. MartÃn-Subero, M., Berk, L., Dodd, S., Kamalesh, V., Maes, M., Kulkarni, J., . . . Berk, M. (2014). Quality of life in bipolar and schizoaffective disorder - A naturalistic approach.*55*, 1540-1545.

546. Martin, J., Padierna, A., Unzurrunzaga, A., Gonzalez, N., Berjano, B., & Quintana, J. M. (2016). Predictors of change in psychosocial impairment secondary to an eating disorder.*243*, 161-167.

547. Marzabadi, E. A., & Zadeh, S. M. H. (2014). The effectiveness of mindfulness training in improving the quality of life of the war victims with post traumatic stress disorder (PTSD).*9*, 228-236.

548. Masaeli, N., Omranifard, V., Maracy, M. R., Kheirabadi, G. R., & Khedri, A. (2016). Validity, reliability and factor analysis of persian version of schizophrenia quality of life scale.*5*, 10.

549. Masa-Font, R., Fernandez-San-Martin, M. I., Martin Lopez, L. M., Alba Munoz, A. M., Oller Canet, S., Martin Royo, J., . . . Salvador Barbarroja, T. (2015). The effectiveness of a program of physical activity and diet to modify cardiovascular risk factors in patients with severe mental illness after 3-month follow-up: CAPiCOR randomized clinical trial.*30*, 1028-1036.

550. Mashiach-Eizenberg, M., Hasson-Ohayon, I., Yanos, P. T., Lysaker, P. H., & Roe, D. (2013). Internalized stigma and quality of life among persons with severe mental illness: The mediating roles of self-esteem and hope.*208*, 15-20.

551. Matejkowski, J., Lee, S., Henwood, B., Lukens, J., & Weinstein, L. C. (2013). Perceptions of health intervene in the relationship between psychiatric symptoms and quality of life for individuals in supportive housing.*40*, 469-475.

552. Matin, B. K., Jalilian, F., Alavije, M. M., Ashtarian, H., Mahboubi, M., & Afsar, A. (2014). Using the PRECEDE model in understanding determinants of quality of life among iranian male addicts.*6*, 19-27.

553. Matovic, S., & Jankovic, S. (2015). Quality of life among patients with depression.*16*, 151-156.

554. Matthies, S., Sadohara-Bannwarth, C., Lehnhart, S., Schulte-Maeter, J., & Philipsen, A. (2016). The impact of depressive symptoms and traumatic experiences on quality of life in adults with ADHD.

555. Mattos, P., LouzÃ£, M. R., Palmini, A. L. F., de Oliveira, I. R., & Rocha, F. L. (2013). A multicenter, open-label trial to evaluate the quality of life in adults with ADHD treated with long-acting methylphenidate (OROS MPH): Concerta quality of life (CONQoL) study.*17*, 444-448.

556. MauriÃ±o, J., Cordero, L., & Ballesteros, J. (2012). The subjective well-being under neuroleptic scale - short version (SWN-K) and the SF-36 health survey as quality of life measures in patients with schizophrenia.*6*, 83-85.

557. Mazza, M., Mandelli, L., Zaninotto, L., Nicola, M. D., Martinotti, G., Harnic, D., . . . Janiri, L. (2012). Bipolar disorder: "pure" versus mixed depression over a 1-year follow-up.*16*, 113-120.

558. McCall, W. V., Lisanby, S. H., Rosenquist, P. B., Dooley, M., Husain, M. M., Knapp, R. G., . . . Kellner, C. H. (2017). Effects of a right unilateral ultrabrief pulse electroconvulsive therapy course on health related quality of life in elderly depressed patients.*209*, 39-45.

559. McCall, W. V., Reboussin, D., Prudic, J., Haskett, R. F., Isenberg, K., Olfson, M., . . . Sackeim, H. A. (2013). Poor health-related quality of life prior to ECT in depressed patients normalizes with sustained remission after ECT.*147*, 107-111.

560. McIntyre, R. S., Soczynska, J. Z., Woldeyohannes, H. O., Alsuwaidan, M. T., Cha, D. S., Carvalho, A. F., . . . Kennedy, S. H. (2015). The impact of cognitive impairment on perceived workforce performance: Results from the international mood disorders collaborative project.*56*, 279-282.

561. McIntyre-Smith, A., St Cyr, K., & King, L. (2015). Sexual functioning among a cohort of treatment-seeking canadian military personnel and veterans with psychiatric conditions.*180*, 817-824.

562. McKenzie, D. P., Sim, M. R., Clarke, D. M., Forbes, A. B., Ikin, J. F., & Kelsall, H. L. (2015). Developing a brief depression screen and identifying associations with comorbid physical and psychological illness in australian gulf war veterans.*79*, 566-573.

563. McMillan, K. A., Asmundson, G. J. G., & Sareen, J. (2017). Comorbid PTSD and social anxiety disorder: Associations with quality of life and suicide attempts.

564. Medeiros, L. G., da Silva, R. A., Souza, L. D. M., da Silva, G. G., Pinheiro, R. T., & Jansen, K. (2015). Posttraumatic stress disorder: Prevalences, comorbidities and quality of life in a community sample of young adults.*64*, 1-7.

565. Medeiros-Ferreira, L., Navarro-Pastor, J. B., ZÃºÃ±iga-Lagares, A., RomanÃ, R., Muray, E., & Obiols, J. E. (2016). Perceived needs and health-related quality of life in people with schizophrenia and metabolic syndrome: A 'real-world' study.*16*

566. Medeiros-Ferreira, L., Obiols, J. E., Navarro-Pastor, J. B., & ZÃºÃ±iga-Lagares, A. (2013). Metabolic syndrome and health-related quality of life in patients with schizophrenia.*41*, 17-26.

567. Medici, C. R., Vestergaard, C. H., Hjorth, P., Hansen, M. V., Shanmuganathan, J. W., Viuff, A. G., & Munk-JÃ¸rgensen, P. (2016). Quality of life and clinical characteristics in a nonselected sample of patients with schizophrenia.*62*, 12-20.

568. Meesters, P. D., Comijs, H. C., de Haan, L., Smit, J. H., Eikelenboom, P., Beekman, A. T. F., & Stek, M. L. (2013). Subjective quality of life and its determinants in a catchment area based population of elderly schizophrenia patients.*147*, 275-280.

569. Metz, V. E., Comer, S. D., Wuerzl, J., Pribasnig, A., & Fischer, G. (2014). Characteristics and quality of life of opioid-dependent pregnant women in austria.*17*, 529-539.

570. Mezey, G., White, S., Thachil, A., Berg, R., Kallumparam, S., Nasiruddin, O., . . . Killaspy, H. (2013). Development and preliminary validation of a measure of social inclusion for use in people with mental health problems: The SInQUE.*59*, 501-507.

571. Miasso, A. I., Telles Filho, P. C., Borges, T. L., Pereira Junior, A. D., Vedana, K. G., Shasanmi, R. O., & Gimenes, F. R. (2017). Quality of life in brazil's family health strategy: Common mental disorders, use of psychotropic drugs and sociodemographic factors.*31*, 68-72.

572. Michalak, E. E., Guiraud-Diawara, A., & Sapin, C. (2014). Asenapine treatment and health-related quality of life in patients experiencing bipolar i disorder with mixed episodes: Post-hoc analyses of pivotal trials.*30*, 711-718.

573. Michalak, E. E., Torres, I. J., Bond, D. J., Lam, R. W., & Yatham, L. N. (2013). The relationship between clinical outcomes and quality of life in first-episode mania: A longitudinal analysis.*15*, 188-198.

574. Michel, P., Auquier, P., Baumstarck, K., Loundou, A., Ghattas, B., LanÃ§on, C., & Boyer, L. (2015). How to interpret multidimensional quality of life questionnaires for patients with schizophrenia?*24*, 2483-2492.

575. Michel, P., Baumstarck, K., Lancon, C., Ghattas, B., Loundou, A., Auquier, P., & Boyer, L. (2017). Modernizing quality of life assessment: Development of a multidimensional computerized adaptive questionnaire for patients with schizophrenia., 1-14.

576. Micoulaud-Franchi, J. A., Faugere, M., Boyer, L., Cermolacce, M., Fond, G., Richieri, R., . . . Lancon, C. (2016). Sensory gating deficits and impaired quality of life in patients with schizophrenia: A preliminary study.*28*, 225-233.

577. Mihalopoulos, C., Chen, G., Iezzi, A., Khan, M. A., & Richardson, J. (2014). Assessing outcomes for cost-utility analysis in depression: Comparison of five multi-attribute utility instruments with two depression-specific outcome measures.*205*, 390-397.

578. Mihalopoulos, C., Magnus, A., Lal, A., Dell, L., Forbes, D., & Phelps, A. (2015). Is implementation of the 2013 australian treatment guidelines for posttraumatic stress disorder cost-effective compared to current practice? A cost-utility analysis using QALYs and DALYs.*49*, 360-376.

579. MihanoviÄ‡, M., Restek-PetroviÄ‡, B., BogoviÄ‡, A., IveziÄ‡, E., Bodor, D., & PoÅ□gain, I. (2015). Quality of life of patients with schizophrenia treated in foster home care and in outpatient treatment.*11*, 585-595.

580. Miller, C. J., Abraham, K. M., Bajor, L. A., Lai, Z., Kim, H. M., Nord, K. M., . . . Kilbourne, A. M. (2013). Quality of life among patients with bipolar disorder in primary care versus community mental health settings.*146*, 100-105.

581. Mishra, A., Sai Krishna, G., Sravani, A., Kurian, T. D., Kurian, J., Ramesh, M., & Kishor, M. (2017). Impact of pharmacist-led collaborative patient education on medication adherence and quality of life of schizophrenia patients in a tertiary care setting.

582. Miskowiak, K., Petersen, J., Ott, C., Knorr, U., Kessing, L., Gallagher, P., & Robinson, L. (2016). Predictors of the discrepancy between objective and subjective cognition in bipolar disorder: A novel methodology.*134*, 511-521.

583. Misri, S., & Swift, E. (2015). Generalized anxiety disorder and major depressive disorder in pregnant and postpartum women: Maternal quality of life and treatment outcomes.*37*, 798-803.

584. Mitchison, D., Dawson, L., Hand, L., Mond, J., & Hay, P. (2016). Quality of life as a vulnerability and recovery factor in eating disorders: A community-based study.*16*

585. Mitchison, D., Hay, P., Engel, S., Crosby, R., Grange, D. L., Lacey, H., . . . Touyz, S. (2013). Assessment of quality of life in people with severe and enduring anorexia nervosa: A comparison of generic and specific instruments.*13*

586. Mitchison, D., Hay, P., Mond, J., & Slewa-Younan, S. (2013). Self-reported history of anorexia nervosa and current quality of life: Findings from a community-based study.*22*, 273-281.

587. Mitchison, D., Hay, P., Slewa-Younan, S., & Mond, J. (2012). Time trends in population prevalence of eating disorder behaviors and their relationship to quality of life.*7*

588. Mitchison, D., Morin, A., Mond, J., Slewa-Younan, S., & Hay, P. (2015). The bidirectional relationship between quality of life and eating disorder symptoms: A 9-year community-based study of australian women.*10*

589. Modabbernia, A., Yaghoubidoust, M., Lin, C. Y., Fridlund, B., Michalak, E. E., Murray, G., & Pakpour, A. H. (2016). Quality of life in iranian patients with bipolar disorder: A psychometric study of the persian brief quality of life in bipolar disorder (QoL.BD).*25*, 1835-1844.

590. Mond, J., Hay, P., Rodgers, B., & Owen, C. (2012). Quality of life impairment in a community sample of women with eating disorders.*46*, 561-568.

591. Montemagni, C., Castagna, F., Crivelli, B., De Marzi, G., Frieri, T., MacrÃ¬, A., & Rocca, P. (2014). Relative contributions of negative symptoms, insight, and coping strategies to quality of life in stable schizophrenia.*220*, 102-111.

592. Montgomery, W., Kadziola, Z., Ye, W., Xue, H. B., Liu, L., & Treuer, T. (2015). Correlation between changes in quality of life and symptomatic improvement in chinese patients switched from typical antipsychotics to olanzapine.*11*, 177-183.

593. Monti, F., Agostini, F., Paterlini, M., Andrei, F., De Pascalis, L., Palomba, S., & La Sala, G. B. (2015). Effects of assisted reproductive technology and of women's quality of life on depressive symptoms in the early postpartum period: A prospective case-control study.*31*, 374-378.

594. Montoya, A., Lebrec, J., Keane, K. M., Fregenal, I., Ciudad, A., Morinigo, A., . . . Zimmerman, M. (2016). Broader conceptualization of remission assessed by the remission from depression questionnaire and its association with symptomatic remission: A prospective, multicenter, observational study.*16*, 352.

595. Moore, L., Carr, A., Hodgins, S., Duffy, D., & Rooney, B. (2017). What works best for reducing symptoms and improving quality of life? A 6-months follow-up study on the effectiveness of group cognitive behaviour therapy and group information and support for adults suffering from depression., 1-11.

596. Moran, K., & Priebe, S. (2016). Better quality of life in patients offered financial incentives for taking anti-psychotic medication: Linked to improved adherence or more money?*25*, 1897-1902.

597. Moreira, E. G., Correia, D. G., BonifÃ¡cio, K. L., Moraes, J. B. D., Cavicchioli, F. L., Nunes, C. S., . . . Maes, M. (2017). Lowered PON1 activities are strongly associated with depression and bipolar disorder, recurrence of (hypo)mania and depression, increased disability and lowered quality of life., 1-13.

598. Moreno, F. A., Chong, J., Dumbauld, J., Humke, M., & Byreddy, S. (2012). Use of standard webcam and internet equipment for telepsychiatry treatment of depression among underserved hispanics.*63*, 1213-1217.

599. Morenz, R., Woolverton, C., Frost, R. B., Kiewel, N. A., & Breitborde, N. J. K. (2015). Clinical correlates of distorted auditory perception in first-episode psychosis.*9*, 248-251.

600. Morrison, S. C., Brown, L. A., & Cohen, A. S. (2013). A multidimensional assessment of social cognition in psychometrically defined schizotypy.*210*, 1014-1019.

601. Morton, E., Murray, G., Michalak, E. E., Lam, R. W., Beaulieu, S., Sharma, V., . . . Yatham, L. N. (2017). Quality of life in bipolar disorder: Towards a dynamic understanding., 1-10.

602. Mosanya, T. J., Adelufosi, A. O., Adebowale, O. T., Ogunwale, A., & Adebayo, O. K. (2014). Self-stigma, quality of life and schizophrenia: An outpatient clinic survey in nigeria.*60*, 377-386.

603. Moslehi, S., Atefimanesh, P., & Asgharnejad Farid, A. (2015). The relationship between problem-focused coping strategies and quality of life in schizophrenic patients.*29*, 288.

604. Mosqueiro, B. P., Da Rocha, N. S., & Fleck, M. P. D. A. (2015). Intrinsic religiosity, resilience, quality of life, and suicide risk in depressed inpatients.*179*, 128-133.

605. Moss, P., Mandy, W., & Howlin, P. (2017). Child and adult factors related to quality of life in adults with autism., 1-8.

606. Mourady, D., Richa, S., Karam, R., Papazian, T., Moussa, F. H., El-Osta, N., . . . Khabbaz, L. R. (2017). Associations between quality of life, physical activity, worry, depression and insomnia: A cross-sectional designed study in healthy pregnant women.*12*

607. MuÃ±oz, P., Quintana, J. M., Las Hayas, C., Padierna, A., Aguirre, U., & GonzÃ¡lez-Torres, M. A. (2012). Quality of life and motivation to change in eating disorders. perception patient-psychiatrist.*13*, 131-134.

608. Munikanan, T., Midin, M., Daud, T. I. M., Rahim, R. A., Bakar, A. K. A., Jaafar, N. R. N., . . . Baharuddin, N. (2017). Association of social support and quality of life among people with schizophrenia receiving community psychiatric service: A cross-sectional study.*75*, 94-102.

609. Murphy, K. M., McGuire, A. P., Erickson, T. M., & Mezulis, A. H. (2017). Somatic symptoms mediate the relationship between health anxiety and health-related quality of life over eight weeks.*33*, 244-252.

610. Naber, D., Baker, R. A., Eramo, A., Forray, C., Hansen, K., Sapin, C., . . . Potkin, S. G. (2017). Long-term effectiveness of aripiprazole once-monthly for schizophrenia is maintained in the QUALIFY extension study.

611. Naber, D., Peuskens, J., Schwarzmann, N., Goltz, M., Kruger, H., Lambert, M., & Haro, J. M. (2013). Subjective well-being in schizophrenia: A randomised controlled open-label 12-month non-inferiority study comparing quetiapine XR with risperidone (RECOVER).*23*, 1257-1269.

612. Nachar, N., Guay, S., Beaulieu-Prevost, D., & Marchand, A. (2013). Assessment of the psychosocial predictors of health-related quality of life in a PTSD clinical sample.*19*, 20-27.

613. Nakagawa, S., & Hayashi, N. (2013). Clinical correlates of objective and subjective quality of life among middle-aged and elderly female inpatients with chronic schizophrenia.*6*, 389-393.

614. Nakamura, H., Watanabe, N., & Matsushima, E. (2014). Structural equation model of factors related to quality of life for community-dwelling schizophrenic patients in japan.*8*

615. Nejtek, V. A., Allison, N., & Hilburn, C. (2012). Race- and gender-related differences in clinical characteristics and quality of life among outpatients with psychotic disorders.*18*, 329-337.

616. Ng, L. C., Petruzzi, L. J., Greene, M. C., Mueser, K. T., Borba, C. P. C., & Henderson, D. C. (2016). Posttraumatic stress disorder symptoms and social and occupational functioning of people with schizophrenia.*204*, 590-598.

617.Ng, P., Pan, J. Y., Lam, P., & Leung, A. (2014). Quality of life in people with mental illness in non-residential community mental health services in hong kong.*24*, 43-50.

618.Ng, T. H., & Johnson, S. L. (2013). Rejection sensitivity is associated with quality of life, psychosocial outcome, and the course of depression in euthymic patients with bipolar i disorder.*37*, 1169-1178.

619.Ngai, F. W., Wong, P. W. C., Chung, K. F., & Leung, K. Y. (2017). The effect of a telephone-based cognitive behavioral therapy on quality of life: A randomized controlled trial.*20*, 421-426.

620.Niles, A. N., Sherbourne, C. D., Roy-Byrne, P. P., Stein, M. B., Sullivan, G., Bystritsky, A., & Craske, M. G. (2013). Anxiety treatment improves physical functioning with oblique scoring of the SF-12 short form health survey.*35*, 291-296.

621.Niolu, C., Bianciardi, E., Di Lorenzo, G., Marchetta, C., Barone, Y., Sterbini, N., . . . Siracusano, A. (2015). Enhancing adherence, subjective well-being and quality of life in patients with schizophrenia: Which role for long-acting risperidone?*5*, 278-288.

622.Nogueira, J. M., & RodrÃguez-MÃguez, E. (2015). Using the SF-6D to measure the impact of alcohol dependence on health-related quality of life.*16*, 347-356.

623.Nolan, J. A., McEvoy, J. P., Koenig, H. G., Hooten, E. G., Whetten, K., & Pieper, C. F. (2012). Religious coping and quality of life among individuals living with schizophrenia.*63*, 1051-1054.

624.Nooripour, R., de Velasco, B. P., ZadeMohammadi, A., Ventegod, S., Bayles, C., Blossom, P., & Apsche, J. (2014). Effectiveness of quality of life therapy on sexual self-efficacy and quality of life in addicted couples.*9*, 43-45.

625.Nosyk, B., Bray, J. W., Wittenberg, E., Aden, B., Eggman, A. A., Weiss, R. D., . . . Schackman, B. R. (2015). Short term health-related quality of life improvement during opioid agonist treatment.*157*, 121-128.

626.Novick, D., Montgomery, W., Kadziola, Z., Moneta, V., Peng, X., Brugnoli, R., & Haro, J. M. (2013). Do concomitant pain symptoms in patients with major depression affect quality of life even when taking into account baseline depression severity?*7*, 463-470.

627.Novick, D., Montgomery, W., Moneta, M. V., Peng, X., Brugnoli, R., & Haro, J. M. (2015). Chinese patients with major depression: Do concomitant pain symptoms affect quality of life independently of severity of depression?*19*, 174-181.

628. Novick, D., Perrin, E., Bertsch, J., & Knapp, M. (2014). Symptomatic remission and patient quality of life in an observational study of schizophrenia: Is there a relationship?
629. Novick, D., Montgomery, W., Aguado, J., Peng, X., & Haro, J. M. (2017). Factors associated with and impact of pain persistence in asian patients with depression: A 3-month, prospective observational study.*21*, 29-35.
630. Nuernberg, G. L., Baeza, F. L., Fleck, M. P., & Rocha, N. S. (2016). Outcomes of inpatients with severe mental illness: A naturalistic descriptive study.*38*, 141-147.
631. Nyer, M., Farabaugh, A., Fehling, K., Soskin, D., Holt, D., Papakostas, G. I., . . . Mischoulon, D. (2013). Relationship between sleep disturbance and depression, anxiety, and functioning in college students.*30*, 873-880.
632. Oâ€™Keeffe, D., Hickey, D., Lane, A., McCormack, M., Lawlor, E., Kinsella, A., . . . Clarke, M. (2016). Mental illness self-management: A randomised controlled trial of the wellness recovery action planning intervention for inpatients and outpatients with psychiatric illness.*33*, 81-92.
633. Odlaug, B., Gual, A., DeCourcy, J., Perry, R., Pike, J., Heron, L., & Rehm, J. (2016). Alcohol dependence, co-occurring conditions and attributable burden.*51*, 201-209.
634. Oei, T. P., & McAlinden, N. M. (2014). Changes in quality of life following group CBT for anxiety and depression in a psychiatric outpatient clinic.*220*, 1012-1018.
635. Oei, T. P. S., McAlinden, N. M., & Cruwys, T. (2014). Exploring mechanisms of change: The relationships between cognitions, symptoms, and quality of life over the course of group cognitive-behaviour therapy.*168*, 72-77.
636. Ofir-Eyal, S., Hasson-Ohayon, I., & Kravetz, S. (2014). Affective and cognitive empathy and social quality of life in schizophrenia: A comparison between a parallel process model and an integrative meditation model.*220*, 51-57.
637. Ofir-Eyal, S., Hasson-Ohayon, I., Bar-Kalifa, E., Kravetz, S., & Lysaker, P. H. (2017). Agreement between clients with schizophrenia and mental health workers on clients' social quality of life: The role of social cognition and symptoms.*90*, 125-137.

638. Ogawa, S., Kondo, M., Okazaki, J., Imai, R., Ino, K., Furukawa, T. A., & Akechi, T. (2017). The relationships between symptoms and quality of life over the course of cognitive-behavioral therapy for panic disorder in japan.*9*

639. Ogunnubi, O. P., Olagunju, A. T., Aina, O. F., & Okubadejo, N. U. (2017). Medication adherence among nigerians with schizophrenia: Correlation between clinico-demographic factors and quality of life.*9*, 6889.

640. Ohmuro, N., Matsumoto, K., Ishii, Y., Katsura, M., Obara, C., Kikuchi, T., . . . Matsuoka, H. (2017). The associations between quality of life and clinical symptoms in individuals with an at-risk mental state and first-episode psychosis.*254*, 54-59.

641. Ojeda, N., SÃ¡nchez, P., PeÃ±a, J., ElizagÃ¡rate, E., Yoller, A. B., GutiÃ©rrez-Fraile, M., . . . Napal, O. (2012). An explanatory model of quality of life in schizophrenia: The role of processing speed and negative symptoms.*40*, 10-18.

642. Oldis, M., Murray, G., Macneil, C. A., Hasty, M. K., Daglas, R., Berk, M., . . . Cotton, S. M. (2016). Trajectory and predictors of quality of life in first episode psychotic mania.*195*, 148-155.

643. Oliveira, S. E. H., Carvalho, H., & Esteves, F. (2016). Internalized stigma and quality of life domains among people with mental illness: The mediating role of self-esteem.*25*, 55-61.

644. Oliveira, S. E. H., Carvalho, H., & Esteves, F. (2016). Toward an understanding of the quality of life construct: Validity and reliability of the WHOQOL-bref in a psychiatric sample.*244*, 37-44.

645. Olose, E. O., Edet, J., Igwe, M. N., Chukwujekwu, D. C., Aguocha, M. C., & Uwakwe, R. (2017). Dyslipidaemia and medical outcome (health related quality of life) in patients with schizophrenia taking antipsychotics in enugu, nigeria.*2017*, 9410575.

646. Omranifard, V., Rajabi, F., Mohammadian-Sichani, M., & Maracy, M. (2015). The effect of add-on memantine on global function and quality of life in schizophrenia: A randomized, double-blind, controlled, clinical trial.*4*, 211.

647. Omranifard, V., Yari, A., Kheirabadi, G. R., Rafizadeh, M., Maracy, M. R., & Sadri, S. (2014). Effect of needs-assessment-based psychoeducation for families of patients with schizophrenia on quality of life of patients and their families: A controlled study.*3*, 125.

648. Ong, C., Sagayadevan, V., Lee, S. P., Ong, R., Chong, S. A., Frost, R. O., & Subramaniam, M. (2016). Hoarding among outpatients seeking treatment at a psychiatric hospital in singapore.*8*, 56-63.

649. Ooms, P., Mantione, M., Figee, M., Schuurman, P. R., Van Den Munckhof, P., & Denys, D. (2014). Deep brain stimulation for obsessive-compulsive disorders: Long-term analysis of quality of life.*85*, 153-158.

650. Opaas, M., & Varvin, S. (2015). Relationships of childhood adverse experiences with mental health and quality of life at treatment start for adult refugees traumatized by pre-flight experiences of war and human rights violations.*203*, 684-695.

651. Oppezzo, M. A., Michalek, A. K., Delucchi, K., Baiocchi, M. T. M., Barnett, P. G., & Prochaska, J. J. (2016). Health-related quality of life among veterans in addictions treatment: Identifying behavioral targets for future intervention.*25*, 1949-1957.

652. Ostman, M. (2014). Low satisfaction with sex life among people with severe mental illness living in a community.*216*, 340-345.

653. Othman, Z., & Ghazali, M. (2017). Validation of the quality of life scale (QLS) malay version among patients with schizophrenia.*24*, 24-26.

654. Othman, Z., Ghazali, M., Razak, A. A., & Husain, M. (2013). Severity of tardive dyskinesia and negative symptoms are associated with poor quality of life in schizophrenia patients.*20*, 677-680.

655. Ow, C. Y., & Lee, B. O. (2015). Relationships between perceived stigma, coping orientations, self-esteem, and quality of life in patients with schizophrenia.*27*, NP1932-NP1941.

656. Oyanadel, C., & Buela-Casal, G. (2014). Time perception and psychopathology: Influence of time perspective on quality of life of severe mental illness.*42*, 99-107.

657. Ozdemir, O., Boysan, M., Guzel Ozdemir, P., & Yilmaz, E. (2015). Relationships between posttraumatic stress disorder (PTSD), dissociation, quality of life, hopelessness, and suicidal ideation among earthquake survivors.*228*, 598-605.

658. Oznur, T., Akarsu, S., Erdem, M., Durusu, M., Toygar, M., Poyrazoglu, Y., . . . Ozmenler, K. N. (2015). Psychiatric symptoms and quality of life in military personnel deployed abroad.*52*, 60-64.

659. Pagotto, L. F., Mendlowicz, M. V., Coutinho, E. S. F., Figueira, I., Luz, M. P., Araujo, A. X., & Berger, W. (2015). The impact of posttraumatic symptoms and comorbid mental disorders on the health-related quality of life in treatment-seeking PTSD patients.*58*, 68-73.

660. Painter, J. M., Gray, K., McGinn, M. M., Mostoufi, S., & Hoerster, K. D. (2016). The relationships of posttraumatic stress disorder and depression symptoms with health-related quality of life and the role of social support among veterans.*25*, 2657-2667.

661. Paiva, C. B., Ferreira, I. B., Bosa, V. L., & Narvaez, J. C. M. (2017). Depression, anxiety, hopelessness and quality of life in users of cocaine/crack in outpatient treatment.*39*, 34-42.

662. Palijan, T. á., Kovaevi, D., Vlastelica, M., Dadi-Hero, E., & Sarilar, M. (2017). Quality of life of persons suffering from schizophrenia, psoriasis and physical disabilities.*29*, 60-65.

663. Pan, A. W., Chen, Y. L., Chung, L. I., Wang, J. D., Chen, T. J., & Hsiung, P. C. (2012). A longitudinal study of the predictors of quality of life in patients with major depressive disorder utilizing a linear mixed effect model.*198*, 412-419.

664. Panayiotou, G., & Karekla, M. (2013). Perceived social support helps, but does not buffer the negative impact of anxiety disorders on quality of life and perceived stress.*48*, 283-294.

665. Park, J. E., Suk, H. W., Seong, S. J., Sohn, J. H., Hahm, B., Lee, D., & Cho, M. J. (2016). Association between personality traits and mental health outcomes in older adults with lifetime trauma exposure: A nationwide community sample.*28*, 1533-1543.

666. Park, S., Cho, M. J., Seong, S., Shin, S. Y., Sohn, J., Hahm, B. J., & Hong, J. P. (2012). Psychiatric morbidities, sleep disturbances, suicidality, and quality-of-life in a community population with medically unexplained pain in korea.*198*, 509-515.

667. Park, S. C., Sakong, J. K., Koo, B. H., Kim, J. M., Jun, T. Y., Lee, M. S., . . . Park, Y. C. (2016). Potential relationship between season of birth and clinical characteristics in major depressive disorder in koreans: Results from the CRESCEND study.*57*, 784-789.

668. Park, S. G., Bennett, M. E., Couture, S. M., & Blanchard, J. J. (2013). Internalized stigma in schizophrenia: Relations with dysfunctional attitudes, symptoms, and quality of life.*205*, 43-47.

669. Parker, G., & Fletcher, K. (2013). The 'real world' utility of a web-based bipolar disorder screening measure: A replication study.*150*, 276-283.

670. Patra, B. N., Sarkar, S., Basu, D., & Mattoo, S. K. (2016). Quality of life of opioid- and alcohol-dependent treatment seeking men in north india.*21*, 317-322.

671. Patra, S., & Mishra, A. (2012). Association of psychopathology with quality of life in acute phase of schizophrenia; an experience from east india.*21*, 104-108.

672. Pattanayak, R. D., Sagar, R., & Mehta, M. (2012). Neuropsychological performance in euthymic indian patients with bipolar disorder type I: Correlation between quality of life and global functioning.*66*, 553-563.

673. Patterson, M., Moniruzzaman, A., Palepu, A., Zabkiewicz, D., Frankish, C. J., Krausz, M., & Somers, J. M. (2013). Housing first improves subjective quality of life among homeless adults with mental illness: 12-month findings from a randomized controlled trial in vancouver, british columbia.*48*, 1245-1259.

674. Pawaskar, M., Agh, T., Radewonuk, J., Voko, Z., McElroy, S. L., Herman, B. K., & Gasior, M. (2015). Longitudinal modeling of the relationship between lisdexamfetamine dimesylate and health-related quality of life in adults with moderate to severe binge eating disorder.*18*, A407.

675. Pawlak, I. E., WoliÅ„ska, W., & Mroczek, B. (2016). Impact of climacteric and depressive symptoms on the quality of life of postmenopausal women.*18*, 325-331.

676. Pearlman-Avnion, S., Cohen, N., & Eldan, A. (2017). Sexual well-being and quality of life among high-FunctioningÂ Adults with autism.*35*, 279-293.

677. Pejuskovic, B., Lecic-Tosevski, D., & Toskovic, O. (2017). Longitudinal study of posttraumatic stress disorder in the community: Risk and recovery factors.*205*, 77-82.

678. Peluso, M. J., Lewis, S. W., Barnes, T. R. E., & Jones, P. B. (2013). Non-neurological and metabolic side effects in the cost utility of the latest antipsychotics in schizophrenia randomised controlled trial (CUtLASS-1).*144*, 80-86.

679. Penner-Goeke, K., Henriksen, C. A., Chateau, D., Latimer, E., Sareen, J., & Katz, L. Y. (2015). Reductions in quality of life associated with common mental disorders: Results from a nationally representative sample.*76*, 1506-1512.

680. Peraica, T., VidoviÄ‡, A., PetroviÄ‡, Z. K., & KozariÄ‡-KovaÄ□iÄ‡, D. (2014). Quality of life of croatian veterans' wives and veterans with posttraumatic stress disorder.*12*

681. Pereira, M., Pedras, S., & Lopes, C. (2012). Posttraumatic stress, psychological morbidity, pychopathology, family functioning, and quality of life in portuguese war veterans.*18*, 49-58.

682. Perna, G., Cavedini, P., Harvey, P. D., Di Chiaro, N. V., Dacco, S., & Caldirola, D. (2016). Does neuropsychological performance impact on real-life functional achievements in obsessive-compulsive disorder? A preliminary study.*20*, 224-231.

683. PetroviÄ‡-KitiÄ‡, A., & JankoviÄ‡, S. (2017). Translation, cultural adjustment & evaluation of reliability & validity of "quality of life enjoyment & satisfaction questionnaire - short form" for patients with schizophrenia.*34*, 35-42.

684. Phalen, P. L., Viswanadhan, K., Lysaker, P. H., & Warman, D. M. (2015). The relationship between cognitive insight and quality of life in schizophrenia spectrum disorders: Symptom severity as potential moderator.*230*, 839-s845.

685. Pham, T., & SaloppÃ©, X. (2013). Influence of psychopathy on self-perceived quality of life in forensic patients: A cohort study in belgium.*24*, 31-47.

686. Picco, L., Pang, S., Lau, Y. W., Jeyagurunathan, A., Satghare, P., Abdin, E., . . . Subramaniam, M. (2016). Internalized stigma among psychiatric outpatients: Associations with quality of life, functioning, hope and self-esteem.*246*, 500-506.

687. Pietrini, F., Spadafora, M., Talamba, G. A., Godini, L., Lelli, L., Arcabasso, S., . . . Ballerini, A. (2015). The effects of switching from oral to LAI antipsychotic treatment on subjective experience of schizophrenic and schizoaffective patients: Preliminary results.*19*, 106-113.

688. Pietrzak, R. H., el-Gabalawy, R., Tsai, J., Sareen, J., Neumeister, A., & Southwick, S. M. (2014). Typologies of posttraumatic stress disorder in the U.S. adult population.*162*, 102-106.

689. Pinho, L. G. d., Pereira, A., & Chaves, C. (2017). Influence of sociodemographic and clinical characteristics on the quality of life of patients with schizophrenia.*51*, e03244.

690. Pini, S. (2014). The relationships among separation anxiety disorder, adult attachment style and agoraphobia in patients with panic disorder.*28*, 741-746.

691. Pinna, F., Deriu, L., Lepori, T., Maccioni, R., Milia, P., Sarritzu, E., . . . Cagliari Recovery Study, G. (2013). Is it true remission? A study of remitted patients affected by schizophrenia and schizoaffective disorders.*210*, 739-744.

692. PitkÃ□nen, A., VÃ□limÃ□ki, M., Kuosmanen, L., Katajisto, J., Koivunen, M., HÃ□tÃ¶nen, H., . . . Knapp, M. (2012). Patient education methods to support quality of life and functional ability among patients with schizophrenia: A randomised clinical trial.*21*, 247-256.

693. Pitknen, A., Vlimki, M., Endicott, J., Katajisto, J., Luukkaala, T., Koivunen, M., . . . HtÃ¶nen, H. (2012). Assessing quality of life in patients with schizophrenia in an acute psychiatric setting: Reliability, validity and feasibility of the EQ-5D and the Q-LES-Q.*66*, 19-25.

694. Pittman, J. O., Goldsmith, A. A., Lemmer, J. A., Kilmer, M. T., & Baker, D. G. (2012). Post-traumatic stress disorder, depression, and health-related quality of life in OEF/OIF veterans.*21*, 99-103.

695. Poc, O. G., Ruiz-Iriondo, M., & Bobowik, M. (2013). Multi-informant perception of quality of life and adaptation in chronic schizophrenia.*16*

696. Pohjolainen, V., Koponen, S., RÃ□sÃ□nen, P., Roine, R. P., Sintonen, H., & Karlsson, H. (2016). Long-term health-related quality of life in eating disorders.*25*, 2341-2346.

697. Pohjolainen, V., RÃ□sÃ□nen, P., Roine, R. P., Sintonen, H., Koponen, S., & Karlsson, H. (2017). Cost-effectiveness of anorexia nervosa in QALYs.*71*, 67-71.

698. Pohjolainen, V., Ryynanen, O. P., Rasanen, P., Roine, R. P., Koponen, S., & Karlsson, H. (2015). Bayesian prediction of treatment outcome in anorexia nervosa: A preliminary study.*69*, 210-215.

699. Pollack, L. O., McCune, A. M., Mandal, K., & Lundgren, J. D. (2015). Quantitative and qualitative analysis of the quality of life of individuals with eating disorders.*17*

700. Portugal, F. B., Campos, M. R., GonÃ§alves, D. A., Mari, J. J., Gask, L., Bower, P., . . . Fortes, S. (2014). Psychiatric morbidity and quality of life of primary care attenders in two cities in brazil.*63*, 23-32.

701. Pos, K., de Wit, I. E., van Dijk, F. A., Bartels-Velthuis, A. A., Bruggeman, R., Meijer, C. J., . . . van Winkel, R. (2017). An experience sampling study on the ecological validity of the SWN-20: Indication that subjective well-being is associated with momentary affective states above and beyond psychosis susceptibility.

702. Powell, V. B., de Oliveira, O. H., Seixas, C., Almeida, C., Grangeon, M. C., Caldas, M., . . . de-Oliveira, I. R. (2013). Changing core beliefs with trial-based cognitive therapy may improve quality of life in social phobia: A randomized study.*35*, 243-247.

703. Priebe, S., Matanov, A., Demi, N., Blagovcanin Simic, J., Jovanovic, S., Gajic, M., . . . Muijen, M. (2012). Community mental health centres initiated by the south-eastern europe stability pact: Evaluation in seven countries.*48*, 352-362.

704. Prigent, A., Auraaen, A., Kamendje-Tchokobou, B., Durand-Zaleski, I., & Chevreul, K. (2014). Health-related quality of life and utility scores in people with mental disorders: A comparison with the non-mentally ill general population.*11*, 2807-2817.

705. Prisciandaro, J. J., Desantis, S. M., & Bandyopadhyay, D. (2012). Simultaneous modeling of the impact of treatments on alcohol consumption and quality of life in the COMBINE study: A coupled hidden markov analysis.*36*, 2141-2149.

706. Pupo, M. C., Serafim, P. M., & de Mello, M. F. (2015). Health-related quality of life in posttraumatic stress disorder: 4 years follow-up study of individuals exposed to urban violence.*228*, 741-745.

707. Qiao, J., Qian, L. J., Zhao, H. F., Gong, G. H., & Geng, D. Q. (2017). The relationship between quality of life and clinical phenotype in patients with treatment resistant and non-treatment resistant depression.*21*, 2432-2436.

708. Qu, W., Gu, S., Luo, H., Tang, Q., & Guo, J. (2014). Effects of olanzapine-fluoxetine combination treatment of major depressive disorders on the quality of life during acute treatment period.*70*, 1799-1802.

709. Raab, P. A., Mackintosh, M. A., Gros, D. F., & Morland, L. A. (2015). Impact of comorbid depression on quality of life in male combat veterans with posttraumatic stress disorder.*52*, 563-576.

710. Rabinowitz, J., Berardo, C. G., Bugarski-Kirola, D., & Marder, S. (2013). Association of prominent positive and prominent negative symptoms and functional health, well-being, healthcare-related quality of life and family burden: A CATIE analysis.*150*, 339-342.

711. Rabinowitz, J., Levine, S. Z., Garibaldi, G., Bugarski-Kirola, D., Berardo, C. G., & Kapur, S. (2012). Negative symptoms have greater impact on functioning than positive symptoms in schizophrenia: Analysis of CATIE data.*137*, 147-150.

712. Radhakrishnan, M., McCrone, P., Lafortune, L., Everard, L., Fowler, D., Amos, T., . . . Birchwood, M. (2017). Cost-effectiveness of early intervention services for psychosis and fidelity to national policy implementation guidance.

713. Radhakrishnan, R., Menon, J., Kanigere, M., Ashok, M., Shobha, V., & Galgali, R. B. (2012). Domains and determinants of quality of life in schizophrenia and systemic lupus erythematosus.*34*, 49-55.

714. Raisch, D. W., Campbell, H. M., Garnand, D. A., Jones, M. A., Sather, M. R., Naik, R., & Ling, W. (2012). Health-related quality of life changes associated with buprenorphine treatment for opioid dependence.*21*, 1177-1183.

715. Rajagopalan, K., Bacci, E. D., Ng-Mak, D., Wyrwich, K., Pikalov, A., & Loebel, A. (2016). Effects on health-related quality of life in patients treated with lurasidone for bipolar depression: Results from two placebo controlled bipolar depression trials.*16*

716. Ralevski, E., Gianoli, M. O., McCarthy, E., & Petrakis, I. (2014). Quality of life in veterans with alcohol dependence and co-occurring mental illness.*39*, 386-391.

717. Ramaprasad, D., Rao, N. S., & Kalyanasundaram, S. (2015). Disability and quality of life among elderly persons with mental illness.*18*, 31-36.

718. Rao, R., Ambekar, A., Yadav, S., Sethi, H., & Dhawan, A. (2012). Slow-release oral morphine as a maintenance agent in opioid dependence syndrome: An exploratory study from india.*17*, 294-300.

719. Rayan, A., & Obiedate, K. (2017). The correlates of quality of life among jordanian patients with schizophrenia., 1078390317710498.

720. Reed, P., Giles, A., Gavin, M., Carter, N., & Osborne, L. A. (2016). Loneliness and social anxiety mediate the relationship between autism quotient and quality of life in university students.*28*, 723-733.

721. Reininghaus, U., McCabe, R., Burns, T., Croudace, T., & Priebe, S. (2012). The validity of subjective quality of life measures in psychotic patients with severe psychopathology and cognitive deficits: An item response model analysis.*21*, 237-246.

722. Remmerswaal, K. C. P., Batelaan, N. M., Smit, J. H., van Oppen, P., & van Balkom, A. J. L. M. (2016). Quality of life and relationship satisfaction of patients with obsessive compulsive disorder.*11*, 56-62.

723. Rentala, S., Fong, T. C., Nattala, P., Chan, C. L., & Konduru, R. (2015). Effectiveness of body-mind-spirit intervention on well-being, functional impairment and quality of life among depressive patients - a randomized controlled trial.*71*, 2153-2163.

724. Renwick, L., Drennan, J., Sheridan, A., Owens, L., Lyne, J., O'Donoghue, B., . . . Clarke, M. (2017). Subjective and objective quality of life at first presentation with psychosis.*11*, 401-410.

725.Renwick, L., Jackson, D., Foley, S., Owens, E., Ramperti, N., Behan, C., . . . O'Callaghan, E. (2012). Depression and quality of life in first-episode psychosis.*53*, 451-455.

726.Renwick, L., Lyne, J., Owens, E., B, O. D., Madigan, K., Turner, N., . . . E, O. C. (2013). Comparison of generic and disease-specific measures of quality of life in first-episode psychosis.*47*, 1403-1408.

727.Reynolds, K., Pietrzak, R. H., Mackenzie, C. S., Chou, K. L., & Sareen, J. (2016). Post-traumatic stress disorder across the adult lifespan: Findings from a nationally representative survey.*24*, 81-93.

728.Richardson, J., Sinha, K., Iezzi, A., & Khan, M. A. (2014). Modelling utility weights for the assessment of quality of life (AQoL)-8D.*23*, 2395-2404.

729.Ridgewell, C., Blackford, J. U., McHugo, M., & Heckers, S. (2017). Personality traits predicting quality of life and overall functioning in schizophrenia.*182*, 19-23.

730.RiihimÃ□ki, K., Sintonen, H., Vuorilehto, M., JylhÃ□, P., Saarni, S., & IsometsÃ□, E. (2016). Health-related quality of life of primary care patients with depressive disorders.*37*, 28-34.

731.Ritsner, M. S., Arbitman, M., Lisker, A., & Ponizovsky, A. M. (2012). Ten-year quality of life outcomes among patients with schizophrenia and schizoaffective disorder II. predictive value of psychosocial factors.*21*, 1075-1084.

732.Ritsner, M. S., & Grinshpoon, A. (2015). Ten-year quality-of-life outcomes of patients with schizophrenia and schizoaffective disorders: The relationship with unmet needs for care.*9*, 125-134A.

733.Ritsner, M. S., Lisker, A., & Arbitman, M. (2012). Ten-year quality of life outcomes among patients with schizophrenia and schizoaffective disorders: I. predictive value of disorder-related factors.*21*, 837-847.

734.Ritsner, M. S., Lisker, A., Arbitman, M., & Grinshpoon, A. (2012). Factor structure in the camberwell assessment of need-patient version: The correlations with dimensions of illness, personality and quality of life of schizophrenia patients.*66*, 499-507.

735.Ritsner, M. S., Lisker, A., & Grinshpoon, A. (2014). Predicting 10-year quality-of-life outcomes of patients with schizophrenia and schizoaffective disorders.*68*, 308-317.

736.Roberts, J., Lenton, P., Keetharuth, A. D., & Brazier, J. (2014). Quality of life impact of mental health conditions in england: Results from the adult psychiatric morbidity surveys.*12*

737.Robles-Martinez, M., Garcia-Carretero, M. A., Gibert, J., Palma-Alvarez, R. F., Abad, A. C., Sorribes, M., & Roncero, C. (2017). Differences between cravings and health-related quality of life in patients with alcohol dependence with or without dual pathology in outpatient treatment: A descriptive study.

738.Rocca, P., Montemagni, C., Mingrone, C., Crivelli, B., Sigaudo, M., & Bogetto, F. (2016). A cluster-analytical approach toward real-world outcome in outpatients with stable schizophrenia.*32*, 48-54.

739.Rodriguez, M., Spaniel, F., Konradova, L., Sedlakova, K., Dvorska, K., Prajsova, J., . . . Fajnerova, I. (2015). Comparison of visuospatial and verbal abilities in first psychotic episode of schizophrenia spectrum disorder: Impact on global functioning and quality of life.*9*, 322.

740.Rofail, D., Regnault, A., le Scouiller, S., Berardo, C. G., Umbricht, D., & Fitzpatrick, R. (2016). Health-related quality of life in patients with prominent negative symptoms: Results from a multicenter randomized phase II trial on bitopertin.*25*, 201-211.

741.Romm, K. L., Melle, I., Thoresen, C., Andreassen, O. A., & Rossberg, J. I. (2012). Severe social anxiety in early psychosis is associated with poor premorbid functioning, depression, and reduced quality of life.*53*, 434-440.

742.Roopesh Gopal, N., Sudarshan, C., & Kumar, S. G. (2014). Relationship of quality of life with disability grade in obsessive compulsive disorder and dysthymic disorder.*20*, 212-214.

743.Rubio, J. M., Olfson, M., PÃ©rez-Fuentes, G., Garcia-Toro, M., Wang, S., & Blanco, C. (2014). Effect of first episode axis I disorders on quality of life.*202*, 271-274.

744.Rubio, J. M., Olfson, M., Villegas, L., PeÅ•ez-Fuentes, G., Wang, S., & Blanco, C. (2013). Quality of life following remission of mental disorders: Findings from the national epidemiologic survey on alcohol and related conditions.*74*, e445-e450.

745.SÃ¡nchez, J., Rosenthal, D. A., Tansey, T. N., Frain, M. P., & Bezyak, J. L. (2016). Predicting quality of life in adults with severe mental illness: Extending the international classification of functioning, disability, and health.*61*, 19-31.

746.SÃ¸rensen, T., Giraldi, A., & Vinberg, M. (2017). Sexual distress and quality of life among women with bipolar disorder.*5*

747. Sabes-Figuera, R., Knapp, M., Bendeck, M., Mompart-Penina, A., & Salvador-Carulla, L. (2012). The local burden of emotional disorders. an analysis based on a large health survey in catalonia (spain).*26*, 24-29.

748. Sachs, G., Winklbaur, B., Jagsch, R., Lasser, I., Kryspin-Exner, I., Frommann, N., & Wolwer, W. (2012). Training of affect recognition (TAR) in schizophrenia-impact on functional outcome.*138*, 262-267.

749. Saengcharnchai, P., Likhitsathian, S., Yingwiwattanapong, J., Wittayanookulluk, A., Uttawichai, K., Boonchareon, H., & Srisurapanont, M. (2016). Correlates of health-related quality of life in thai patients with alcohol dependence.*15*, 210-220.

750. Said, A. M., Okasha, A., Okasha, T., Haroon, A., & Fikry, M. (2012). Quality of life and personality dimensions in egyptian substance dependence patients.*11*, 36-42.

751. Salmabadi, M., Faroogh Sadeghbojd, M., Farshad, M. R., & Zolfaghari, S. (2016). Comparing the spiritual health and quality of life in addicted and non-addicted patients in the city of birjand, iran.*5*, e23208.

752. Sarkar, S., Balhara, Y. P. S., Kumar, S., Saini, V., Kamran, A., Patil, V., . . . Gyawali, S. (2017). Internalized stigma among patients with substance use disorders at a tertiary care center in india., 1-14.

753. Sarkin, A. J., Groessl, E. J., Carlson, J. A., Tally, S. R., Kaplan, R. M., Sieber, W. J., & Ganiats, T. G. (2013). Development and validation of a mental health subscale from the quality of well-being self-administered.*22*, 1685-1696.

754. Sarma, S. I., & Byrne, G. J. (2014). Relationship between anxiety and quality of life in older mental health patients.*33*, 201-204.

755. Savill, M., Orfanos, S., Reininghaus, U., Wykes, T., Bentall, R., & Priebe, S. (2016). The relationship between experiential deficits of negative symptoms and subjective quality of life in schizophrenia.*176*, 387-391.

756. Schel, S. H., Bouman, Y. H., & Bulten, B. H. (2015). Quality of life in long-term forensic psychiatric care: Comparison of self-report and proxy assessments.*29*, 162-167.

757. Schel, S. H. H., Bouman, Y. H. A., Vorstenbosch, E. C. W., & Bulten, B. H. (2017). Development of the forensic inpatient quality of life questionnaire: Short version (FQL-SV).*26*, 1153-1161.

758. Schlegel, S., Hartmann, A., Fuchs, R., & Zeeck, A. (2015). The freiburg sport therapy program for eating disordered outpatients: A pilot study.*20*, 319-327.

759. Schlosser, D., Campellone, T., Kim, D., Truong, B., Vergani, S., Ward, C., & Vinogradov, S. (2016). Feasibility of PRIME: A cognitive neuroscience-informed mobile app intervention to enhance motivated behavior and improve quality of life in recent onset schizophrenia.*5*, e77.

760. Schmidt, S. J., Lange, M., SchÃ¶ttle, D., Karow, A., Schimmelmann, B. G., & Lambert, M. (2017). Negative symptoms, anxiety, and depression as mechanisms of change of a 12-month trial of assertive community treatment as part of integrated care in patients with first- and multi-episode schizophrenia spectrum disorders (ACCESS I trial)., 1-10.

761. Schnurr, P. P., & Lunney, C. A. (2012). Work-related outcomes among female veterans and service members after treatment of posttraumatic stress disorder.*63*, 1072-1079.

762. Schnurr, P. P., & Lunney, C. A. (2016). SYMPTOM BENCHMARKS of IMPROVED QUALITY of LIFE in PTSD.*33*, 247-255.

763. Schroeder, K., Huber, C. G., Jelinek, L., & Moritz, S. (2013). Subjective well-being, but not subjective mental functioning shows positive associations with neuropsychological performance in schizophrenia-spectrum disorders.*54*, 824-830.

764. Schuch, F. B., Vasconcelos-Moreno, M. P., Borowsky, C., Zimmermann, A. B., Rocha, N. S., & Fleck, M. P. (2015). Exercise and severe major depression: Effect on symptom severity and quality of life at discharge in an inpatient cohort.*61*, 25-32.

765. Schulte-van Maaren, Carlier, I. V. E., Zitman, F. G., van Hemert, A. M., de Waal, M. W. M., van Noorden, M. S., & Giltay, E. J. (2012). Reference values for generic instruments used in routine outcome monitoring: The leiden routine outcome monitoring study.*12*

766. Schwab, B., Danie, H. S., Lutkemeyer, C., Neves, J. A. L. L., Zilli, L. N., Guarnieri, R., . . . Michels, A. M. M. P. (2015). Variables associated with health-related quality of life in a brazilian sample of patients from a tertiary outpatient clinic for depression and anxiety disorders.*37*, 202-208.

767. Schwartzman, C. M., Boisseau, C. L., Sibrava, N. J., Mancebo, M. C., Eisen, J. L., & Rasmussen, S. A. (2017). Symptom subtype and quality of life in obsessive-compulsive disorder.*249*, 307-310.

768. Seligowski, A. V., Miron, L. R., & Orcutt, H. K. (2015). Relations among self-compassion, PTSD symptoms, and psychological health in a trauma-exposed sample.*6*, 1033-1041.

769. Senin, T., Franz, M., Deuschle, M., Bergemann, N., Kammerer-Ciernioch, J., Lautenschlager, M., & Meyer, T. (2017). QLiS-SF: Development of a short form of the quality of life in schizophrenia questionnaire.*17*

770. Serafini, G., Gonda, X., Pompili, M., Rihmer, Z., Amore, M., & Engel-Yeger, B. (2016). The relationship between sensory processing patterns, alexithymia, traumatic childhood experiences, and quality of life among patients with unipolar and bipolar disorders.*62*, 39-50.

771. Shabani, A., Ahmadzad-Asl, M., Zangeneh, K., Teimurinejad, S., Kokar, S., Taban, M., . . . Shariat, S. V. (2013). Quality of life in patients with bipolar i disorder: Is it related to disorder outcome?*51*, 386-393.

772. Shadmi, E., Gelkopf, M., Garber-Epstein, P., Baloush-Kleinman, V., Doudai, R., & Roe, D. (2017). Routine patient reported outcomes as predictors of psychiatric rehospitalization.

773. Shah, D., Vaidya, V., Patel, A., Borovicka, M., & Goodman, M. H. (2017). Assessment of health-related quality of life, mental health status and psychological distress based on the type of pharmacotherapy used among patients with depression.*26*, 969-980.

774. Shannon, S., Roche, E., Madigan, K., Renwick, L. J., Dolan, C., Devitt, P., . . . O'Donoghue, B. (2015). Quality of life and functioning one year after experiencing accumulated coercive events during psychiatric admission.*66*, 883-887.

775. Sharif, F., Nourian, K., Ashkani, H., & Zoladl, M. (2012). The effect of psycho-educational intervention on the life quality of major depressive patients referred to hospitals affiliated to shiraz university of medical sciences in shiraz-iran.*17*, 425-429.

776. Sharma, M. P., Salvi, D., & Sharma, M. K. (2012). Sattva, rajas and tamas factors and quality of life in patients with anxiety disorders: A preliminary investigation.*57*, 388-391.

777. Shimizu, Y., Kitagawa, N., Mitsui, N., Fujii, Y., Toyomaki, A., Hashimoto, N., . . . Kusumi, I. (2013). Neurocognitive impairments and quality of life in unemployed patients with remitted major depressive disorder.*210*, 913-918.

778. Shimodera, S., Watanabe, N., Furukawa, T. A., Katsuki, F., Fujita, H., Sasaki, M., & Perlis, M. L. (2014). Change in quality of life after brief behavioral therapy for insomnia in concurrent depression: Analysis of the effects of a randomized controlled trial.*10*, 433-439.

779. Shin, Y. J., Joo, Y. H., & Kim, J. H. (2016). Self-perceived cognitive deficits and their relationship with internalized stigma and quality of life in patients with schizophrenia.*12*, 1411-1417.
780. Shrestha, S., Stanley, M. A., Wilson, N. L., Cully, J. A., Kunik, M. E., Novy, D. M., . . . Oude Voshaar, R. C. (2015). Predictors of change in quality of life in older adults with generalized anxiety disorder.*27*, 1207-1215.
781. Siani, C., de Peretti, C., Millier, A., Boyer, L., & Toumi, M. (2016). Predictive models to estimate utility from clinical questionnaires in schizophrenia: Findings from EuroSC.*25*, 925-934.
782. Sibrava, N. J., Boisseau, C. L., Eisen, J. L., Mancebo, M. C., & Rasmussen, S. A. (2016). An empirical investigation of incompleteness in a large clinical sample of obsessive compulsive disorder.*42*, 45-51.
783. Siegrist, K., Millier, A., Amri, I., AballÃ©a, S., & Toumi, M. (2015). Association between social contact frequency and negative symptoms, psychosocial functioning and quality of life in patients with schizophrenia.*230*, 860-866.
784. Silverberg, N. D., Wojtowicz, M., Bui, E., Wershba, R., Zafonte, R., Laifer, L. M., . . . Iverson, G. L. (2017). Contribution of perceived cognitive functioning to quality of life in service members and veterans with posttraumatic stress disorder.*30*, 318-322.
785. Silverman, M. J. (2013). Effects of group songwriting on depression and quality of life in acute psychiatric inpatients: A randomized three group effectiveness study.*22*, 131-148.
786. Simon, J., Anand, P., Gray, A., Rugkasa, J., Yeeles, K., & Burns, T. (2013). Operationalising the capability approach for outcome measurement in mental health research.*98*, 187-196.
787. Simpson, A., Flood, C., Rowe, J., Quigley, J., Henry, S., Hall, C., . . . Bowers, L. (2014). Results of a pilot randomised controlled trial to measure the clinical and cost effectiveness of peer support in increasing hope and quality of life in mental health patients discharged from hospital in the UK.*14*
788. Singh, P. A., Ganachari Madiwalayya, S., Bheemsain, T., & Shashikala, W. (2017). The impact of clinical pharmacist lead collaborative care on quality of life of the patients with bipolar disorder: A unicenter prospective, randomization study.*51*, S129-S135.
789. Singh, S., Wetterneck, C. T., Williams, M. T., & Knott, L. E. (2016). The role of shame and symptom severity on quality of life in obsessive-compulsive and related disorders.*11*, 49-55.

790. Siqveland, J., Nygaard, E., Hussain, A., Tedeschi, R. G., & Heir, T. (2015). Posttraumatic growth, depression and posttraumatic stress in relation to quality of life in tsunami survivors: A longitudinal study.*13*

791. Siu, C. O., Harvey, P. D., Agid, O., Waye, M., Brambilla, C., Choi, W. K., & Remington, G. (2015). Insight and subjective measures of quality of life in chronic schizophrenia.*2*, 127-132.

792. Sofko, C. A., Currier, J. M., & Drescher, K. D. (2016). Prospective associations between changes in mental health symptoms and health-related quality of life in veterans seeking posttraumatic stress disorder residential treatment.*29*, 630-643.

793. Sohn, J. H., Ahn, S. H., Seong, S. J., Ryu, J. M., & Cho, M. J. (2013). Prevalence, work-loss days and quality of life of community dwelling subjects with depressive symptoms.*28*, 280-286.

794. Soininen, P., Putkonen, H., Joffe, G., Korkeila, J., Puukka, P., PitkÃ□nen, A., & VÃ□limÃ□ki, M. (2013). Does experienced seclusion or restraint affect psychiatric patients' subjective quality of life at discharge?*7*

795. Solvason, H. B., Husain, M., Fitzgerald, P. B., Rosenquist, P., McCall, W. V., Kimball, J., . . . Lisanby, S. H. (2014). Improvement in quality of life with left prefrontal transcranial magnetic stimulation in patients with pharmacoresistant major depression: Acute and six month outcomes.*7*, 219-225.

796. Sonntag, M., Konig, H. H., & Konnopka, A. (2015). The responsiveness of the EQ-5D and time trade-off scores in schizophrenia, affective disorders, and alcohol addiction.*13*, 114.

797. Sonntag, M., Konnopka, A., Leichsenring, F., Salzer, S., Beutel, M. E., Herpertz, S., . . . Konig, H. H. (2013). Reliability, validity and responsiveness of the EQ-5D in assessing and valuing health status in patients with social phobia.*11*, 215.

798. Sousa, T. V., Viveiros, V., Chai, M. V., Vicente, F. L., Jesus, G., Carnot, M. J., . . . Ferreira, P. L. (2015). Reliability and validity of the portuguese version of the generalized anxiety disorder (GAD-7) scale.*13*, 50.

799. Srisurapanont, M., Hong, J. P., Tian-Mei, S., Hatim, A., Liu, C. Y., Udomratn, P., . . . Md, R. A. N. (2013). Clinical features of depression in asia: Results of a large prospective, cross-sectional study.*5*, 259-267.

800. Srivastava, S., & Bhatia, M. S. (2013). Quality of life as an outcome measure in the treatment of alcohol dependence.*22*, 41-46.

801. Stallvik, M., & Clausen, T. (2017). HRQoL and its association with clinical severity and service needs among individuals with a substance use disorder.*22*, 524-530.

802. Stefanatou, P., Giannouli, E., Konstantakopoulos, G., Vitoratou, S., & Mavreas, V. (2014). Measuring the needs of mental health patients in greece: Reliability and validity of the greek version of the camberwell assessment of need.*60*, 662-671.

803. Steiner, A. J., Boulos, N., Mirocha, J., Wright, S. M., Collison, K. L., & IsHak, W. W. (2017). Quality of life and functioning in comorbid posttraumatic stress disorder and major depressive disorder after treatment with citalopram monotherapy.*40*, 16-23.

804. Steiner, A. J., Boulos, N., Wright, S. M., Mirocha, J., Smith, K., Lopez, E., . . . Ishak, W. W. (2017). Major depressive disorder in patients with doctoral degrees: Patient-reported depressive symptom severity, functioning, and quality of life before and after initial treatment in the STAR*D study.*23*, 328-341.

805. Steiner, A. J., Recacho, J., Vanle, B., Dang, J., Wright, S. M., Miller, J. S., . . . Ishak, W. W. (2017). Quality of life, functioning, and depressive symptom severity in older adults with major depressive disorder treated with citalopram in the STARâˆ—D study.*78*, 897-903.

806. Steiner, A. J., Wright, S. M., Kuhn, T., & IsHak, W. W. (2017). Comorbid social phobia and major depressive disorder: The influence of remission from depression on quality of life and functioning.*12*, 719-736.

807. Stewart, M. O., Raffa, S. D., Steele, J. L., Miller, S. A., Clougherty, K. F., Hinrichsen, G. A., & Karlin, B. E. (2014). National dissemination of interpersonal psychotherapy for depression in veterans: Therapist and patient-level outcomes.*82*, 1201-1206.

808. Stochl, J., Croudace, T., Perez, J., Birchwood, M., Lester, H., Marshall, M., . . . Jones, P. B. (2013). Usefulness of EQ-5D for evaluation of health-related quality of life in young adults with first-episode psychosis.*22*, 1055-1063.

809. Strassnig, M., Brar, J. S., & Ganguli, R. (2012). Health-related quality of life, adiposity, and sedentary behavior in patients with early schizophrenia: Preliminary study.*5*, 389-394.

810. □trkalj-Ivezi□, S., Vrdoljak, M., Mu□ini□ L. & Agius, M. (2013). The impact of a rehabilitation day centre program for persons suffering from schizophrenia on quality of life, social functioning and self-esteem.*25*, S194-S199.

811. Stroppa, A., & Moreira-Almeida, A. (2013). Religiosity, mood symptoms, and quality of life in bipolar disorder.*15*, 385-393.

812. Stubbs, B., Gardner-Sood, P., Smith, S., Ismail, K., Greenwood, K., Patel, A., . . . Gaughran, F. (2015). Pain is independently associated with reduced health related quality of life in people with psychosis.*230*, 585-591.

813. Studart, P., GalvÃ£o-de Almeida, A., Bezerra-Filho, S., CaribÃ©, A., Reis Afonso, N., Daltro, C., & Miranda-Scippa, A. (2016). Is history of suicidal behavior related to social support and quality of life in outpatients with bipolar I disorder?*246*, 796-802.

814. Stumbo, S. P., Yarborough, B. J., Paulson, R. I., & Green, C. A. (2015). The impact of adverse child and adult experiences on recovery from serious mental illness.*38*, 320-327.

815. Su, C. T., Ng, H. S., Yang, A. L., & Lin, C. Y. (2014). Psychometric evaluation of the short form 36 health survey (SF-36) and the world health organization quality of life scale brief version (WHOQOL-BREF) for patients with schizophrenia.*26*, 980-989.

816. Su, C. T., Yang, A. L., & Lin, C. Y. (2017). Comparing two schizophrenia-specific quality of life instruments in institutionalized people with schizophrenia.

817. Su, C., Yang, A., & Lin, C. (2017). The construct of the schizophrenia quality of life scale revision 4 for the population of taiwan.*2017*

818. Subero, M. M., Berk, L., Dodd, S., Kulkarni, J., De Castella, A., Fitzgerald, P. B., & Berk, M. (2013). To a broader concept of remission: Rating the health-related quality of life in bipolar disorder.*150*, 673-676.

819. Subramaniam, M., Abdin, E., Poon, L. Y., Vaingankar, J. A., Lee, H., Chong, S. A., & Verma, S. (2014). EQ-5D as a measure of programme outcome: Results from the singapore early psychosis intervention programme.*215*, 46-51.

820. Subramaniam, M., Abdin, E., Vaingankar, J. A., & Chong, S. A. (2012). Obsessive--compulsive disorder: Prevalence, correlates, help-seeking and quality of life in a multiracial asian population.*47*, 2035-2043.

821. Subramaniam, M., Abdin, E., Vaingankar, J. A., Nan, L., Heng, D., McCrone, P., & Chong, S. A. (2013). Impact of psychiatric disorders and chronic physical conditions on health-related quality of life: Singapore mental health study.*147*, 325-330.

822. Subramaniam, M., Abdin, E., Vaingankar, J. A., Picco, L., & Chong, S. A. (2014). Hoarding in an asian population: Prevalence,correlates,disability and quality of life.*43*, 535-543.

823. Subramaniam, M., Abdin, E., Vaingankar, J. A., Wong, K. E., & Chong, S. A. (2015). Comorbid physical and mental illnesses among pathological gamblers: Results from a population based study in singapore.*227*, 198-205.

824. Sudhir, P. M., Sharma, M. P., Mariamma, P., & Subbakrishna, D. K. (2012). Quality of life in anxiety disorders: Its relation to work and social functioning and dysfunctional cognitions: An exploratory study from india.*5*, 309-314.

825. Sugawara, N., Yasui-Furukori, N., Sato, Y., Saito, M., Furukori, H., Nakagami, T., . . . Kaneko, S. (2013). Body mass index and quality of life among outpatients with schizophrenia in japan.*13*

826. Sum, M. Y., Ho, N. F., & Sim, K. (2015). Cross diagnostic comparisons of quality of life deficits in remitted and unremitted patients with schizophrenia and bipolar disorder.*168*, 191-196.

827. Sung, S., Wisniewski, S., Balasubramani, G., Zisook, S., Kurian, B., Warden, D., . . . Rush, A. (2013). Does early-onset chronic or recurrent major depression impact outcomes with antidepressant medications? A CO-MED trial report.*43*, 945-960.

828. Sung, S. C., Porter, E., Robinaugh, D. J., Marks, E. H., Marques, L. M., Otto, M. W., . . . Simon, N. M. (2012). Mood regulation and quality of life in social anxiety disorder: An examination of generalized expectancies for negative mood regulation.*26*, 435-441.

829. Suttajit, S., & Pilakanta, S. (2015). Predictors of quality of life among individuals with schizophrenia.*11*, 1371-1379.

830. Swain, S. P., & Behura, S. S. (2016). A comparative study of quality of life and disability among schizophrenia and obsessive-compulsive disorder patients in remission.*25*, 210-215.

831. Sylvia, L. G., Friedman, E. S., Kocsis, J. H., Bernstein, E. E., Brody, B. D., Kinrys, G., . . . Nierenberg, A. A. (2013). Association of exercise with quality of life and mood symptoms in a comparative effectiveness study of bipolar disorder.*151*, 722-727.

832. Sylvia, L. G., Montana, R. E., Deckersbach, T., Thase, M. E., Tohen, M., Reilly-Harrington, N., . . . Nierenberg, A. A. (2017). Poor quality of life and functioning in bipolar disorder.*5*

833. Sylvia, L. G., Rabideau, D. J., Nierenberg, A. A., Bowden, C. L., Friedman, E. S., Iosifescu, D. V., . . . Reilly-Harrington, N. (2014). The effect of personalized guideline-concordant treatment on quality of life and functional impairment in bipolar disorder.*169*, 144-148.

834. Taha, N. A., Ibrahim, M. I., Rahman, A. F., Shafie, A. A., & Rahman, A. H. (2012). Validation of the schizophrenia quality of life scale revision 4 among chronic schizophrenia patients in malaysia.*1*, 82-86.

835. Taillefer, S. E., Liu, J. J. W., Ornstein, T. J., & Vickers, K. (2016). Indecisiveness as a predictor of quality of life in individuals with obsessive and compulsive traits.*10*, 91-98.

836. Takahashi, T., Higuchi, Y., Komori, Y., Nishiyama, S., Nakamura, M., Sasabayashi, D., . . . Suzuki, M. (2017). Quality of life in individuals with attenuated psychotic symptoms: Possible role of anxiety, depressive symptoms, and socio-cognitive impairments.*257*, 431-437.

837. Talbot, A., Hay, P., & Touyz, S. (2015). Exploring the relationship between cognitive style and daily functioning in patients with anorexia nervosa.*3*, 300-310.

838. Tamburin, S., Federico, A., Faccini, M., Casari, R., Morbioli, L., Sartore, V., . . . Lugoboni, F. (2017). Determinants of quality of life in high-dose benzodiazepine misusers.*14*

839. Tan, E. J., & Rossell, S. L. (2016). Comparing how co-morbid depression affects individual domains of functioning and life satisfaction in schizophrenia.*66*, 53-58.

840. Tan, E. J., Thomas, N., & Rossell, S. L. (2014). Speech disturbances and quality of life in schizophrenia: Differential impacts on functioning and life satisfaction.*55*, 693-698.

841. Tan, S. H., Tang, C., Ng, W. W. N., Ho, C. S. H., & Ho, R. C. M. (2015). Determining the quality of life of depressed patients in singapore through a multiple mediation framework.*18*, 22-30.

842. Tang, I. C., & Wu, H. C. (2012). Quality of life and self-stigma in individuals with schizophrenia.*83*, 497-507.

843. Tas, C., Danaci, A. E., Cubukcuoglu, Z., & Brune, M. (2012). Impact of family involvement on social cognition training in clinically stable outpatients with schizophrenia - A randomized pilot study.*195*, 32-38.

844. Taylor, D. J., Bramoweth, A. D., Grieser, E. A., Tatum, J. I., & Roane, B. M. (2013). Epidemiology of insomnia in college students: Relationship with mental health, quality of life, and substance use difficulties.*44*, 339-348.

845. Teodorescu, D. S., Siqveland, J., Heir, T., Hauff, E., Wentzel-Larsen, T., & Lien, L. (2012). Posttraumatic growth, depressive symptoms, posttraumatic stress symptoms, post-migration stressors and quality of life in multi-traumatized psychiatric outpatients with a refugee background in norway.*10*

846. Tessier, J. M., Erickson, Z. D., Meyer, H. B., Baker, M. R., Gelberg, H. A., Arnold, I. Y., . . . Ames, D. (2017). Therapeutic lifestyle changes: Impact on weight, quality of life, and psychiatric symptoms in veterans with mental illness.*182*, e1738-e1744.

847. Thakurta, R. G., Singh, O. P., Bhattacharya, A., Mallick, A. K., Ray, P., Sen, S., & Das, R. (2012). Nature of sexual dysfunctions in major depressive disorder and its impact on quality of life.*34*, 365-370.

848. Theodore, K., Johnson, S., Chalmers-Brown, A., Doherty, R., Harrop, C., & Ellett, L. (2012). Quality of life and illness beliefs in individuals with early psychosis.*47*, 545-551.

849. Thomas, K. A., Rickwood, D. J., & Brown, P. M. (2017). Symptoms, functioning and quality of life after treatment in a residential sub-acute mental health service in australia.*25*, 243-254.

850. Thomas, S. P., Nisha, A., & Varghese, P. J. (2016). Disability and quality of life of subjects with bipolar affective disorder in remission.*38*, 336-340.

851. Thwin, S. S., Hermes, E., Lew, R., Barnett, P., Liang, M., Valley, D., & Rosenheck, R. (2013). Assessment of the minimum clinically important difference in quality of life in schizophrenia measured by the quality of well-being scale and disease-specific measures.*209*, 291-296.

852. Tobe, M., Nemoto, T., Tsujino, N., Yamaguchi, T., Katagiri, N., Fujii, C., & Mizuno, M. (2016). Characteristics of motivation and their impacts on the functional outcomes in patients with schizophrenia.*65*, 103-109.

853. Touyz, S., Le Grange, D., Lacey, J., Hay, P., Smith, R., Maguire, S., . . . Crosby, R. D. (2016). Treating severe and enduring anorexia nervosa: A randomized controlled trial., 76-94.

854. Tracy, E. M., Laudet, A. B., Min, M. O., Kim, H., Brown, S., Jun, M. K., & Singer, L. (2012). Prospective patterns and correlates of quality of life among women in substance abuse treatment.*124*, 242-249.

855. Tran, B. X., Huong, L. T., Hinh, N. D., Nguyen, L. H., Le, B. N., Nong, V. M., . . . Ho, R. C. (2017). A study on the influence of internet addiction and online interpersonal influences on health-related quality of life in young vietnamese.*17*, 138.

856. Trillini, M. O., & Muller-Vahl, K. R. (2015). Narcissistic vulnerability is a common cause for depression in patients with gilles de la tourette syndrome.*230*, 695-703.

857. Tsai, J., Whealin, J. M., Scott, J. C., Harpaz-Rotem, I., & Pietrzak, R. H. (2012). Examining the relation between combat-related concussion, a novel 5-factor model of posttraumatic stress symptoms, and health-related quality of life in iraq and afghanistan veterans.*73*, 1110-1118.

858. Tsujii, N., Mikawa, W., Tsujimoto, E., Akashi, H., Adachi, T., Kirime, E., & Shirakawa, O. (2016). Relationship between prefrontal hemodynamic responses and quality of life differs between melancholia and non-melancholic depression.*253*, 26-35.

859. Tung, E. S., Tung, M. G., Altenburger, E. M., Pauls, D. L., & Keuthen, N. J. (2014). The relationship between hair pulling style and quality of life.*26*, 193-198.

860. Tungchama, F., Piwuna, C., Armiyaâ€™u, A., Maigari, Y., Davou, F., Goar, S., . . . Uwakwe, R. (2017). Independent socio-demographic and clinical correlates associated with the perception of quality of life of women with postpartum depressionin north-central, nigeria.*21*, 292-301.

861. Turkstra, E., Gamble, J., Creedy, D. K., Fenwick, J., Barclay, L., Buist, A., . . . Scuffham, P. A. (2013). PRIME: Impact of previous mental health problems on health-related quality of life in women with childbirth trauma.*16*, 561-564.

862. Turner, A., Realpe, A. X., Wallace, L. M., & Kosmala-Anderson, J. (2015). A co-produced self-management programme improves psychosocial outcomes for people living with depression.*20*, 242-255.

863. Twohig, M. P., Bluett, E. J., Cullum, J. L., Mitchell, P. R., Powers, P. S., Lensegrav-Benson, T., & Quakenbush-Roberts, B. (2016). Effectiveness and clinical response rates of a residential eating disorders facility.*24*, 224-239.

864. Ubukata, S., Miyata, J., Yoshizumi, M., Uwatoko, T., Hirao, K., Fujiwara, H., . . . Murai, T. (2013). Regional gray matter reduction correlates with subjective quality of life in schizophrenia.*47*, 548-554.

865. Uchimura, N., Kamijo, A., & Takase, T. (2012). Effects of eszopiclone on safety, subjective measures of efficacy, and quality of life in elderly and nonelderly japanese patients with chronic insomnia, both with and without comorbid psychiatric disorders: A 24-week, randomized, double-blind study.*11*

866. Uga, A., Kulkarni, S., Heeramun, V., & Bottum, K. (2017). Evaluation of a model of integrated care for patients with chronic medical and psychiatric illness.*58*, 437-440.

867. Unalan, D., Gocer, S., Basturk, M., Baydur, H., & Ozturk, A. (2015). Coincidence of low social support and high depressive score on quality of life in elderly.

868. Urbach, M., Brunet-Gouet, E., Bazin, N., Hardy-BaylÃ©, M. C., & Passerieux, C. (2013). Correlations of theory of mind deficits with clinical patterns and quality of life in schizophrenia.*4*

869. Uwatoko, T., Yoshizumi, M., Miyata, J., Ubukata, S., Fujiwara, H., Kawada, R., . . . Takahashi, H. (2015). Insular gray matter volume and objective quality of life in schizophrenia.*10*

870. van de Laar, M., Pevernagie, D., van Mierlo, P., & Overeem, S. (2017). Correlates of general quality of life are different in patients with primary insomnia as compared to patients with insomnia and psychiatric comorbidity.*22*, 172-183.

871. van der Plas, A. G., Hoek, H. W., van Hoeken, D., Valencia, E., & van Hemert, A. M. (2012). Perceptions of quality of life and disability in homeless persons with schizophrenia and persons with schizophrenia living in non-institutional housing.*58*, 629-634.

872. Van Der Voort, T. Y. G., Van Meijel, B., Hoogendoorn, A. W., Goossens, P. J. J., Beekman, A. T. F., & Kupka, R. W. (2015). Collaborative care for patients with bipolar disorder: Effects on functioning and quality of life.*179*, 14-22.

873. Vancampfort, D., Guelinckx, H., Probst, M., Stubbs, B., Rosenbaum, S., Ward, P. B., & De Hert, M. (2015). Health-related quality of life and aerobic fitness in people with schizophrenia.*24*, 394-402.

874. Vancampfort, D., Hagemann, N., Wyckaert, S., Rosenbaum, S., Stubbs, B., Firth, J., . . . Sienaert, P. (2017). Higher cardio-respiratory fitness is associated with increased mental and physical quality of life in people with bipolar disorder: A controlled pilot study.*256*, 219-224.

875. Vancampfort, D., Probst, M., Adriaens, A., Pieters, G., De Hert, M., Stubbs, B., . . . Vanderlinden, J. (2014). Changes in physical activity, physical fitness, self-perception and quality of life following a 6-month physical activity counseling and cognitive behavioral therapy program in outpatients with binge eating disorder.*219*, 361-366.

876. Vancampfort, D., Van Damme, T., Probst, M., Firth, J., Stubbs, B., Basangwa, D., & Mugisha, J. (2017). Physical activity is associated with the physical, psychological, social and environmental quality of life in people with mental health problems in a low resource setting.

877. Varghese, J., Edison, J. S., & Vijayaraghavan, R. (2017). Effectiveness of family focused intervention on perceived stress and quality of life among persons with alcohol dependence syndrome.*8*, 65-69.

878. Vasudev, R. G. N., Yallappa, S. C., & Saya, G. K. (2015). Assessment of quality of life (QOL) in obsessive compulsive disorder (OCD) and dysthymic disorder (DD): A comparative study.*9*, VC04-VC07.

879. Vederhus, J. K., Birkeland, B., & Clausen, T. (2016). Perceived quality of life, 6Â months after detoxification: Is abstinence a modifying factor?*25*, 2315-2322.

880. Velloso, P., Piccinato, C., Ferrao, Y., Aliende Perin, E., Cesar, R., Fontenelle, L., . . . do Rosario, M. (2016). The suicidality continuum in a large sample of obsessive-compulsive disorder (OCD) patients.*38*, 1-7.

881. Vemer, P., Bouwmans, C. A., Zijlstra-Vlasveld, M. C., van der Feltz-Cornelis, Christina M., & Hakkaart-van Roijen. (2013). Let's get back to work: Survival analysis on the return-to-work after depression.*9*

882. Vermaire, J. H., van Houtem, C. M. H. H., Ross, J. N., & Schuller, A. A. (2016). The burden of disease of dental anxiety: Generic and disease-specific quality of life in patients with and without extreme levels of dental anxiety.*124*, 454-458.

883. Vibha, P., Saddichha, S., Khan, N., & Akhtar, S. (2013). Quality of life and marital adjustment in remitted psychiatric illness: An exploratory study in a rural setting.*201*, 334-338.

884. Vilhauer, J. S., Cortes, J., Moali, N., Chung, S., Mirocha, J., & Ishak, W. W. (2013). Improving quality of life for patients with major depressive disorder by increasing hope and positive expectations with future directed therapy (FDT).*10*, 12-22.

885. Vilhauer, J. S., Young, S., Kealoha, C., Borrmann, J., Ishak, W. W., Rapaport, M. H., . . . Mirocha, J. (2012). Treating major depression by creating positive expectations for the future: A pilot study for the effectiveness of future-directed therapy (FDT) on symptom severity and quality of life.*18*, 102-109.

886. Voigt, K., Wollburg, E., Weinmann, N., Herzog, A., Meyer, B., Langs, G., & Lowe, B. (2013). Predictive validity and clinical utility of DSM-5 somatic symptom disorder: Prospective 1-year follow-up study.*75*, 358-361.

887. Vorstenbosch, E. C. W., Bouman, Y. H. A., Braun, P. C., & Bulten, E. B. H. (2014). Psychometric properties of the forensic inpatient quality of life questionnaire: Quality of life assessment for long-term forensic psychiatric care.*2*, 335-348.

888. Vrbova, K., Prasko, J., Ociskova, M., & Holubova, M. (2017). Comorbidity of schizophrenia and social phobia â€‘ impact on quality of life, hope, and personality traits: A cross sectional study.*13*, 2073-2083.

889. Vrbova, K., Prasko, J., Ociskova, M., Kamaradova, D., Marackova, M., Holubova, M., . . . Latalova, K. (2017). Quality of life, self-stigma, and hope in schizophrenia spectrum disorders: A cross-sectional study.*13*, 567-576.

890. WÃ□rdig, R. E., Foldemo, A., HultsjÃ¶, S., LindstrÃ¶m, T., & Bachrach-LindstrÃ¶m, M. (2016). An intervention with physical activity and lifestyle counseling improves health-related quality of life and shows small improvements in metabolic risks in persons with psychosis.*37*, 43-52.

891. Wagner, A. F., Stefano, E. C., Cicero, D. C., Latner, J. D., & Mond, J. M. (2016). Eating disorder features and quality of life: Does gender matter?*25*, 2603-2610.

892. Wang, F. Z., Luo, D., Kanb, W., & Wang, Y. (2015). Combined intervention with education and progressive muscle relaxation on quality of life, functional disability, and positive symptoms in patients with acute schizophrenia.*21*, 159-165.

893. Wang, J., & Zhao, X. (2012). Family functioning, social support, and quality of life for chinese empty nest older people with depression.*27*, 1204-1206.

894. Wang, L., Cao, C., Wang, R., Zhang, J., & Li, Z. (2012). The dimensionality of PTSD symptoms and their relationship to health-related quality of life in chinese earthquake survivors.*26*, 711-718.

895. Wang, M. Y., Liu, I. C., Chiu, C. H., & Tsai, P. S. (2016). Cultural adaptation and validation of the chinese version of the fatigue severity scale in patients with major depressive disorder and nondepressive people.*25*, 89-99.

896. Wang, P. W., Wu, H. C., Yen, C. N., Yeh, Y. C., Chung, K. S., Chang, H. C., & Yen, C. F. (2012). Change in quality of life and its predictors in heroin users receiving methadone maintenance treatment in taiwan: An 18-month follow-up dtudy.*38*, 213-219.

897. Wang, X. Q., Petrini, M., & Morisky, D. E. (2016). Comparison of the quality of life, perceived stigma and medication adherence of chinese with schizophrenia: A follow-up study.*30*, 41-46.

898. Wang, X. Q., Petrini, M. A., & Morisky, D. E. (2017). Predictors of quality of life among chinese people with schizophrenia.*19*, 142-148.

899. Wang, Y. X., Xiang, Y. T., Su, Y. A., Li, Q., Shu, L., Ng, C. H., . . . Si, T. M. (2015). Antipsychotic medications in major depression and the association with treatment satisfaction and quality of life: Findings of three national surveys on use of psychotropics in china between 2002 and 2012.*128*, 1847-1852.

900. Wang, Z., & Xu, J. (2016). The relationship between post-traumatic stress disorder and quality of life in infertile shidu parents from the 2008 sichuan earthquake: The moderating role of social support.*23*, 543-553.

901. Wariso, B. A., Guerrieri, G. M., Thompson, K., Koziol, D. E., Haq, N., Martinez, P. E., . . . Schmidt, P. J. (2017). Depression during the menopause transition: Impact on quality of life, social adjustment, and disability.*20*, 273-282.

902. Wartelsteiner, F., Mizuno, Y., Frajo-Apor, B., Kemmler, G., Pardeller, S., Sondermann, C., . . . Hofer, A. (2016). Quality of life in stabilized patients with schizophrenia is mainly associated with resilience and self-esteem.*134*, 360-367.

903. Weigel, A., KÃ¶nig, H. H., Gumz, A., LÃ¶we, B., & Brettschneider, C. (2016). Correlates of health related quality of life in anorexia nervosa.*49*, 630-634.

904. Weinberg, D., Shahar, G., Noyman, G., Davidson, L., McGlashan, T. H., & Fennig, S. (2012). Role of the self in schizophrenia: A multidimensional examination of short-term outcomes.*75*, 285-297.

905. Weisenbach, S. L., Marshall, D., Weldon, A. L., Ryan, K. A., Vederman, A. C., Kamali, M., . . . Langenecker, S. A. (2014). The double burden of age and disease on cognition and quality of life in bipolar disorder.*29*, 952-961.

906. Weisman de Mamani, A., Weintraub, M. J., Maura, J., Martinez de Andino, A., Brown, C. A., & Gurak, K. (2017). Acculturation styles and their associations with psychiatric symptoms and quality of life in ethnic minorities with schizophrenia.*255*, 418-423.

907. Wells, K. B., Jones, L., Chung, B., Dixon, E. L., Tang, L., Gilmore, J., . . . Miranda, J. (2013). Community-partnered cluster-randomized comparative effectiveness trial of community engagement and planning or resources for services to address depression disparities.*28*, 1268-1278.

908. Weltzin, T., Bean, P., Klosterman, E., Lee, H. J., & Welk-Richards, R. (2015). Sex differences in the effects of residential treatment on the quality of life of eating disorder patients.*20*, 301-310.

909. Wesner, A. C., Gomes, J. B., Detzel, T., GuimarÃ£es, L. S., & Heldt, E. (2015). Booster sessions after cognitive-behavioural group therapy for panic disorder: Impact on resilience, coping, and quality of life.*43*, 513-525.

910. Wheeler, A., Denson, L., Neil, C., Tucker, G., Kenny, M., Beltrame, J. F., . . . Proeve, M. (2014). Investigating the effect of mindfulness training on heart rate variability in mental health outpatients: A pilot study.*31*, 175-188.

911. Wheeler, A., McKenna, B., Madell, D., Harrison, J., Prebble, K., Larsson, E., . . . Nakarada-Kordic, I. (2015). Self-reported health-related quality of life of mental health service users with serious mental illness in new zealand.*7*, 117-123.

912. Williams, E., Sands, N., Elsom, S., & Prematunga, R. K. (2015). Mental health consumers' perceptions of quality of life and mental health care.*17*, 299-306.

913. Winter, Y., Epifanova-Bertschi, N., Sankowski, R., Zhukova, T. V., Oertel, W., Dodel, R., & Korchounov, A. (2012). Health-related quality of life and its determinants in the urban russian population with major depressive disorder: A cross-sectional study.*43*, 35-49.

914. Witthauer, C., Gloster, A. T., Meyer, A. H., Goodwin, R. D., & Lieb, R. (2014). Comorbidity of infectious diseases and anxiety disorders in adults and its association with quality of life: A community study.*2*, 80.

915. Wong, N., Sarver, D. E., & Beidel, D. C. (2012). Quality of life impairments among adults with social phobia: The impact of subtype.*26*, 50-57.

916. Woo, J. M., Jeon, H. J., Noh, E., Kim, H. J., Lee, S. W., Lee, K. K., . . . Hong, J. P. (2014). Importance of remission and residual somatic symptoms in health-related quality of life among outpatients with major depressive disorder: A cross-sectional study.*12*

917. Wrocklage, K. M., Schweinsburg, B. C., Krystal, J. H., Trejo, M., Roy, A., Weisser, V., . . . Scott, J. (2016). Neuropsychological functioning in veterans with posttraumatic stress disorder: Associations with performance validity, comorbidities, and functional outcomes.*22*, 399-411.

918. Wu, H. (2014). The health and the human rights-related quality of life of people with severe mental illness in taiwan.*44*, 1216-1232.

919. Xiang, Y. T., Hou, Y. Z., Yan, F., Dixon, L. B., Ungvari, G. S., Dickerson, F., . . . Chiu, H. F. K. (2012). Quality of life in community-dwelling patients with schizophrenia in china.*200*, 584-587.

920. Xiang, Y. T., Li, L. J., Zhou, J. J., Wang, C. Y., Dixon, L. B., Dickerson, F., . . . Chiu, H. F. (2014). Quality of life of patients with euthymic bipolar disorder and its associations with demographic and clinical characteristics, psychopathology, and cognitive deficits.*50*, 44-50.

921. Xiang, Y. T., Wang, Y., Wang, C. Y., Chiu, H. F. K., Chen, Q., Chan, S. S. M., . . . Ungvari, G. S. (2012). Association of insight with sociodemographic and clinical factors, quality of life, and cognition in chinese patients with schizophrenia.*53*, 140-144.

922. Xiao, L., Gao, Y., Zhang, L., Chen, P., & Sun, X. (2016). The relationship between cognitive function and quality of life in euthymic chinese patients with bipolar disorder.*246*, 421-437.

923. Xiao, L., Gao, Y., Zhang, L., Chen, P., Sun, X., & Tang, S. (2016). Adaptation and validation of the â€œtolerability and quality of lifeâ€□ (TOOL) questionnaire in chinese bipolar patients.*25*, 2825-2832.

924. Xiao, L., Gao, Y., Zhang, L., Chen, P., Sun, X., & Tang, S. (2016). Validity and reliability of the brief version of quality of life in bipolar disorder" (bref QoL.BD) among chinese bipolar patients.*193*, 66-72.

925. Xu, G., Cao, Z., Shariff, M., Gu, P., Nguyen, T., Zhou, T., . . . Rao, J. (2016). Effects of G.H.3. on mental symptoms and health-related quality of life among older adults: Results of a three-month follow-up study in shanghai, china.*15*

926. Yamada, K., & Kamagata, E. (2017). Reduction of quality-adjusted life years (QALYs) in patients with premenstrual dysphoric disorder (PMDD)., 1-5.

927. Yan, F., Xiang, Y. T., Hou, Y. Z., Ungvari, G. S., Dixon, L. B., Chan, S. S., . . . Chiu, H. F. (2013). Suicide attempt and suicidal ideation and their associations with demographic and clinical correlates and quality of life in chinese schizophrenia patients.*48*, 447-454.

928. Yang, H. N., Tai, Y. M., Yang, L. K., & Gau, S. S. F. (2013). Prediction of childhood ADHD symptoms to quality of life in young adults: Adult ADHD and anxiety/depression as mediators.*34*, 3168-3181.

929. Yang, W. C., Lin, C. H., Wang, F. C., & Lu, M. J. (2017). Factors related to the improvement in quality of life for depressed inpatients treated with fluoxetine.*17*

930. Yang, Y. J., Xu, Y. M., Chen, W. C., Zhu, J. H., Lu, J., & Zhong, B. L. (2017). Loneliness and its impact on quality of life in chinese heroindependent patients receiving methadone maintenance treatment.*8*, 79803-79808.

931. Ye, W., Fujikoshi, S., Nakahara, N., Takahashi, M., Ascher-Svanum, H., & Ohmori, T. (2012). Improved outcomes following a switch to olanzapine treatment from risperidone treatment in a 1-year naturalistic study of schizophrenia patients in japan.*66*, 313-321.

932. Yee, A., Danaee, M., Loh, H. S., Sulaiman, A. H., & Ng, C. G. (2016). Sexual dysfunction in heroin dependents: A comparison between methadone and buprenorphine maintenance treatment.*11*

933. Yeh, C., Huang, Y., Tang, C., Wang, L., Chou, W., Chou, M., & Chen, C. (2014). Neurocognitive effects of aripiprazole in adolescents and young adults with schizophrenia.*68*, 219-224.

934. Yen, Y. F., Chou, P., Lin, Y. S., & Deng, C. Y. (2015). Factors associated with health-related quality of life among injection drug users at methadone clinics in taipei, taiwan.*78*, 292-298.

935. Zahid, M. A., & Ohaeri, J. U. (2013). Clinical and psychosocial factors associated with needs for care: An arab experience with a sample of treated community-dwelling persons with schizophrenia.*48*, 313-323.

936. Zargar, F., Farid, A. A. A., Atef-Vahid, M. K., Afshar, H., & Omidi, A. (2013). Comparing the effectiveness of acceptance-based behavior therapy and applied relaxation on acceptance of internal experiences, engagement in valued actions and quality of life in generalized anxiety disorder.*18*, 118-122.

937. Zargar, F., Farid, A. A. A., Atef-Vahid, M., Afshar, H., Maroofi, M., & Omranifard, V. (2012). Effect of acceptance-based behavior therapy on severity of symptoms, worry and quality of life in women with generalized anxiety disorder.*6*, 23-32.

938. Zeng, Q., Xu, Y., & Wang, W. C. (2013). Quality of life in outpatients with depression in china.*150*, 513-521.

939. Zeng, Y., Zhou, Y., Lin, J., Zhou, Y., & Yu, J. (2015). Generic and disease-specific quality of life and its predictors among chinese inpatients with schizophrenia.*228*, 724-728.

940. Zhang, B., Ding, X., Lu, W., Zhao, J., Lv, Q., Yi, Z., . . . Chen, Y. (2016). Effect of group cognitive-behavioral therapy on the quality of life and social functioning of patients with mild depression.*28*, 18-27.

941. Zhao, C., Wu, Z., & Xu, J. (2013). The association between post-traumatic stress disorder symptoms and the quality of life among wenchuan earthquake survivors: The role of social support as a moderator.*22*, 733-743.

942. Zhao, N., Wang, X., Wu, W., Hu, Y., Niu, Y., Wang, X., . . . Wang, G. (2017). Gender differences in quality of life and functional disability for depression outpatients with or without residual symptoms after acute phase treatment in china.*219*, 141-148.

943.Zhou, K., Wang, D., Li, H., Wei, X., Yin, J., Liang, P., . . . Zhuang, G. (2017). Bidirectional relationships between retention and health-related quality of life in chinese mainland patients receiving methadone maintenance treatment.*12*

944.Zhou, K., Zhuang, G., Zhang, H., Liang, P., Yin, J., Kou, L., . . . You, L. (2013). Psychometrics of the short form 36 health survey version 2 (SF-36v2) and the quality of life scale for drug addicts (QOL-DAv2.0) in chinese mainland patients with methadone maintenance treatment. erratum appears in PLoS one. 2014;9(2):E89704.*8*, e79828.

945.Zhou, Y., Cao, Z., Yang, M., Xi, X., Guo, Y., Fang, M., . . . Du, Y. (2017). Comorbid generalized anxiety disorder and its association with quality of life in patients with major depressive disorder.*7*, 40511.

946.Zhou, Y., Zhou, R., Li, W., Lin, Y., Yao, J., Chen, J., & Shen, T. (2015). Controlled trial of the effectiveness of community rehabilitation for patients with schizophrenia in shanghai, china.*27*, 167-174.

947.Zhuang, S. M., An, S. H., & Zhao, Y. (2013). Yoga effects on mood and quality of life in chinese women undergoing heroin detoxification: A randomized controlled trial.*62*, 260-268.

948.Zilcha-Mano, S., Dinger, U., McCarthy, K. S., Barrett, M. S., & Barber, J. P. (2014). Changes in well-being and quality of life in a randomized trial comparing dynamic psychotherapy and pharmacotherapy for major depressive disorder.*152-154*, 538-542.

949.Zlotnick, C., Lawental, M., & Pud, D. (2017). Double whammy: Adverse childhood events and pain reflect symptomology and quality of life in women in substance abuse treatment.*56*, 189-201.
